# Supplementary material for: Vertical substitution strategy to enable cooperation between spin–orbit coupling and transition dipoles for organic phosphorescence
Source: Nat Commun. 2026 Mar 17;17:4098. doi: 10.1038/s41467-026-70371-w (PMC13144389; doi:10.1038/s41467-026-70371-w)
Supplement: Supplementary file 1 — Supplementary Information [file 41467_2026_70371_MOESM1_ESM.pdf]

Supplementary Information

Vertical substitution strategy to enable cooperation between spin–orbit coupling and transition dipoles for organic phosphorescence

Kikuya Hayashi[1], Riku Shimura[1], Ryo Miyashita[1], Shuzo Hirata[1*]

1. Department of Engineering Science, The University of Electro-Communications, 1-5-1 Chofugaoka, Chofu, Tokyo 182-8585, Japan

*Corresponding author.

E-mail: shuzohirata@uec.ac.jp

CONTENTS

Suppl. Note 1. Synthesis and characterization of chromophores (Supplementary Figs. 1–14)

Suppl. Note 2. T_1 optimized geometry of chromophores estimated by theoretical calculation (Supplementary Fig. 15)

Suppl. Note 3. Emission behavior of chromophores in amorphous *β*-estradiol under and soon after UV excitation (Supplementary Fig. 16)

Suppl. Note 4. Photophysical characteristics

Suppl. Note 4-1. Determination procedure for emission yield (Supplementary Figs. 17 and 18)

Suppl. Note 4-2. Procedure for making Fig. 2c and reproducibility of Φ_p (Supplementary Fig. 19)

Suppl. Note 4-3. Transient absorption measurements for determining Φ_{isc} (Supplementary Figs. 20–22 and Supplementary Table 1)

Suppl. Note 4-4. Summary of photophysical characteristics relating to RTP (Supplementary Table 2)

Suppl. Note 4-5. Optical properties in solution (Supplementary Fig. 23)

Suppl. Note 5. Theoretical calculations

Suppl. Note 5-1. Calculated values of k_r^T and $<T_1|\mathbf{H}_{SO}|S_0>^2$ (Supplementary Fig. 24)

Suppl. Note 5-2. Relationship between orbital orientation characteristics and spin flip (Supplementary Fig. 25)

Suppl. Note 5-3. Molecular orbitals relating to $<T_1|\mathbf{H}_{SO}|S_0>^2$ (Supplementary Fig. 26)

Suppl. Note 5-4. Calculated parameters relating to k_r^T (Supplementary Fig. 27)

Suppl. Note 5-5. Contribution of $E_{Sn\text{-}S0}$ and $E_{Tm\text{-}S0}$ to k_r^T (Supplementary Fig. 28)

Suppl. Note 5-6. Calculation results and photophysical properties of chromophore **R2** (Supplementary Figs. 29–31 and Supplementary Table 3)

Suppl. Note 5-7. Molecular orbitals relating to $<S_n|\mathbf{H}_{SO}|T_1>^2$ (Supplementary Fig. 32)

Suppl. Note 5-8. Calculation results and photophysical properties of chromophore **R1** (Supplementary Figs. 33–35 and Supplementary Table 4)

Suppl. Note 5-9. Molecular orbitals relating to $\langle T_m|\mathbf{H}_{SO}|S_0\rangle^2$ (Supplementary Fig. 36)

Suppl. Note 6. Microscopic demonstrations

Suppl. Note 6-1. Procedure for preparing water-dispersible nanocrystals

Suppl. Note 6-2. Biocompatibility of the water-dispersible nanocrystal (Supplementary Fig. 37)

Suppl. Note 6-3. High-resolution multi-label afterglow imaging (Supplementary Figs. 38 and 39)

Supplementary References

Suppl. Note 1. Synthesis and characterization of chromophores (Supplementary Figs 1–14)

Synthesized chromophores were characterized using proton nuclear magnetic resonance (^{1}H NMR) spectroscopy (ECA-500, JEOL, Tokyo, Japan), carbon nuclear magnetic resonance (^{13}C NMR) spectroscopy (ECA-500, JEOL), matrix-assisted laser-desorption ionization high-resolution mass spectroscopy (HRMS-MALDI) (JMS-S3000, JEOL), and elemental analysis (Series II CHNS/O 2400 analyzer, PerkinElmer, MA, USA). The detailed synthesis procedure and characterization information are as follows.

Dibenzo[*g*,*p*]chrysen-2-yl(phenyl)selane (2):

2-Bromodibenzo[*g*,*p*]chrysene was prepared by the same manner as a previous report[1]. 2-Bromodibenzo[*g*,*p*]chrysene (150 mg, 0.368 mmol), 1,2-diphenyldiselane (80.5 mg, 0.258 mmol), [1,1′-bis(diphenylphosphino)ferrocene] dichloropalladium(II) (13.5 mg, 0.0184 mmol), and Zinc (33.4 mg, 0.516 mmol) in anhydrous tetrahydrofuran (4.0 mL) was stirred under reflux conditions in a nitrogen atmosphere for 24 h. After cooling to room temperature, chloroform was added and washed with pure water three times. The organic layer was dried with sodium sulfate (Na_2SO_4) and evaporated to yield a crude product. The crude material was purified via column chromatography (silica gel; dichloromethane/hexane (12:88 v/v) as the eluent) to give **2** as a pale-yellow powder (54.9 mg, 30.8%). The powder was purified further by sublimation. ^{1}H NMR ($CDCl_3$, 500 MHz): δ = 8.82 (sd, *J* = 2.0 Hz, 1H), 8.71–8.68 (m, 4H), 8.63 (dd, *J* = 8.3, 1.5 Hz, 1H), 8.59–8.56 (m, 2H), 7.70–7.58 (m, 9H), 7.33–7.30 (m, 3H) (Supplementary Fig. 1a). ^{13}C NMR ($CDCl_3$, 125 MHz): δ = 133.09, 131.59, 131.17, 130.94, 130.90, 130.88, 130.16, 129.72, 129.55, 129.50, 128.45, 129.13, 129.02, 128.90, 128.86, 128.76, 128.30, 127.99, 127.73, 127.52, 127.21, 126.88, 126.72, 126.70, 126.69, 126.64, 123.63, 123.63, 123.60 (Supplementary Fig. 1b). HRMS-MALDI (*m*/*z*): $[M]^+$ calcd. for $C_{32}H_{20}Se$, 484.073; Found 484.073 (Supplementary Fig. 1c). Anal. Calcd. for $C_{32}H_{20}Se$: C, 79.50; H, 4.17; Se, 16.33. Found C, 79.76; H, 4.17.

2,7,10,15-Tetrakis(phenylselanyl)dibenzo[*g*,*p*]chrysene (3):

2,7,10,15-Tetrabromodibenzo[*g*,*p*]chrysene was prepared by a reported procedure with minor alterations as follows[2]. Bromine (456 mg, 2.85 mmol) diluted in anhydrous chlorobenzene (1.5 mL) was added dropwise to anhydrous chlorobenzene (10 mL) dissolving dibenzo[*g*,*p*]chrysene (180 mg, 0.548 mmol) at room temperature under a nitrogen atmosphere. The mixture solution was heated under reflux condition for 24 h. After completion of the reaction, chloroform was added to the reaction mixture and

washed with pure water three times. The organic phase was dried with Na_2SO_4 and evaporated under reduced pressure, then washed with hexane including small amount of chloroform to yield a crude mixture of 2,7,10,15-tetrabromodibenzo[*g*,*p*]chrysene as white powder (321 mg). HRMS-MALDI (*m*/*z*): $[M]^+$ calcd. for $C_{26}H_{12}Br_4$, 643.763; Found 643.762.

The portion of crude mixture of 2,7,10,15-tetrabromodibenzo[*g*,*p*]chrysene (300.0 mg), 1,2-diphenyldiselane (393 mg, 1.26 mmol), [1,1′-bis(diphenylphosphino)ferrocene] dichloropalladium(II) (51.1 mg, 0.0698 mmol), and Zinc (152 mg, 2.33 mmol) was added to anhydrous tetrahydrofuran (12 mL). The mixture solution was stirred at 70 °C under a nitrogen atmosphere for 27 h. After cooling to room temperature, chloroform was added. The undissolved solid was removed by filtration with suction. The organic phase was washed with pure water three times, dried over Na_2SO_4, and evaporated to yield crude material. The crude material was purified by column chromatography (silica gel; dichloromethane/hexane (22:78 v/v) as the eluent) to give **3** as a pale-yellow powder (15.5 mg). The yield after step reactions was 3.2% with consideration of a decrease in the amount of intermediate crude material during the step. The powder was purified further by sublimation. ^{1}H NMR ($CDCl_3$, 500 MHz): δ = 8.66 (sd, *J* = 1.5 Hz, 4H), 8.44 (d, *J* = 8.5 4H), 7.61 (dd, *J* = 8.5, 1.5 Hz, 4H), 7.65–7.54 (m, 8H), 7.32–7.29 (m, 12H) (Supplementary Fig. 2a). ^{13}C NMR ($CDCl_3$, 125 MHz): δ = 133.27, 131.21, 130.83, 130.78, 130.33, 129.52, 129.48, 128.13, 127.75, 127.68, 127.30 (Supplementary Fig. 2b). HRMS-MALDI (*m*/*z*): $[M]^+$ calcd. for $C_{50}H_{32}Se_4$, 949.919; Found 949.922 (Supplementary Fig. 2c). Anal. Calcd. for $C_{50}H_{32}Se_4$: C, 63.30; H, 3.40; Se, 33.30. Found C, 63.52; H, 3.23.

2-(8-Bromonaphthalen-1-yl)dibenzo[*g*,*p*]chrysene:

2-(Dibenzo[*g,p*]chrysen-2-yl)-4,4,5,5-tetramethyl-1,3,2-dioxaborolane was prepared using a previously reported method[3]. 2-(dibenzo[*g*,*p*]chrysen-2-yl)-4,4,5,5-tetramethyl-1,3,2-dioxaborolane (400 mg, 0.880 mmol), 1-bromo-8-iodonaphthalene (440 mg, 1.32 mmol), potassium carbonate (243 mg, 1.76 mmol), tetrakis(triphenylphosphine)palladium(0) (50.9 mg, 0.0440 mmol) were added to anhydrous *N*,*N*-dimethylformamide (24 mL). The mixture solution was heated at 110 °C for 31 h under a nitrogen atmosphere. After completion of the reaction, dichloromethane was added to the reaction solution and washed with pure water three times. The organic phase was dried with Na_2SO_4 and evaporated under reduced pressure. The residue was purified via column chromatography (silica gel; dichloromethane/hexane (18:82 v/v) as the eluent) to give 2-(8-bromonaphthalen-1-yl)dibenzo[*g*,*p*]chrysene as a white powder

(319 mg, 67.9%). ^{1}H NMR ($CDCl_3$, 500 MHz): δ = 8.81 (dd, J = 8.0, 1.5 Hz, 1H), 8.75–8.71 (m, 5H), 8.69–8.67 (m, 2H), 7.97–7.95 (m, 2H), 7.83 (dd, J = 7.5, 1.5 Hz, 1H), 7.72–7.57 (m, 9H), 7.35 (t, J = 7.8 Hz, 1H) (Supplementary Fig. 3a). ^{13}C NMR ($CDCl_3$, 125 MHz): δ = 141.08, 140.29, 136.19, 133.89, 131.59, 131.01, 130.88, 130.84, 130.12, 129.81, 129.46, 129.31, 129.30, 129.13, 129.05, 129.03, 129.01, 128.93, 128.91, 128.11, 127.82, 127.52, 127.51, 126.60, 126.58, 126.53, 126.22, 125.41, 124.93, 123.75, 123.62, 123.60, 120.32 (Supplementary Fig. 3b). HRMS-MALDI (*m*/*z*): $[M]^+$ calcd. for $C_{36}H_{21}Br$, 534.081; Found 534.081 (Supplementary Fig. 3c).

8-(Dibenzo[*g*,*p*]chrysen-2-yl)naphthalen-1-yl)(phenyl)selane (4):

2-(8-Bromonaphthalen-1-yl)dibenzo[*g*,*p*]chrysene (180 mg, 0.337 mmol), 1,2-diphenyldiselane (211 mg, 0.675 mmol), [1,1′-bis(diphenylphosphino)ferrocene]dichloropalladium(II) (24.7 mg, 0.0337 mmol), and Zinc (55.2 mg, 0.844 mmol) in anhydrous tetrahydrofuran (4.0 mL) was reacted under reflux condition at a nitrogen atmosphere for 90 h. Dichloromethane was added to the reaction solution and washed with water three times. The organic layer was dried over Na_2SO_4 and evaporated under vacuum to give a crude material. The crude material was purified by column chromatography (silica gel; dichloromethane/hexane (20:80 v/v) as the eluent) to yield **4** as a pale-yellow powder (79.3 mg, 38.6%). Furthermore, **4** was purified by sublimation. ^{1}H NMR (acetone-d_6, 500 MHz): δ = 8.91–8.77 (m, 8H), 8.08–8.06 (m, 1H), 7.95 (d, J = 10 Hz, 1H), 7.84 (d, J = 10 Hz, 1H), 7.8–7.73 (m, 6H), 7.70–7.67 (m, 2H), 7.37–7.33 (m, 2H), 7.28–7.20 (m, 5H) (Supplementary Fig. 4a). ^{13}C NMR ($CDCl_3$, 125 MHz): δ = 140.56, 140.51, 135.34, 135.22, 133.68, 133.00, 132.12, 131.84, 131.04, 130.93, 130.90, 130.45, 130.25, 129.53, 129.32, 129.23, 129.14, 128.97, 128.89, 128.72, 128.68, 128.54, 127.78, 127.73, 127.53, 127.43, 126.64, 126.62, 126.61, 126.59, 126.10, 125.42, 125.10, 123.96, 123.64, 123.59 (Supplementary Fig. 4b). HRMS-MALDI (*m*/*z*): $[M]^+$ calcd. for $C_{42}H_{26}Se$, 610.120; Found 610.117 (Supplementary Fig. 4c). Anal. Calcd. for $C_{42}H_{26}Se$: C, 82.75; H, 4.30; Se, 12.95. Found C, 82.71; H, 4.17.

2,7,10,15-Tetrakis(8-bromonaphthalen-1-yl)dibenzo[*g*,*p*]chrysene:

The crude mixture of 2,7,10,15-tetrabromodibenzo[*g*,*p*]chrysene was prepared by the same procedure as intermediate material of **3** as follows. Bromine (487 mg, 3.04 mmol) diluted in anhydrous chlorobenzene (1.6 mL) was added slowly to anhydrous chlorobenzene (11 mL) dissolving dibenzo[*g*,*p*]chrysene (200 mg, 0.609 mmol) at room temperature under a nitrogen atmosphere. The mixture solution was heated under reflux condition for 24 h. After completion of the reaction, chloroform was added to the reaction

mixture and washed with pure water three times. The organic phase was dried with Na_2SO_4 and evaporated under low pressure, then washed with hexane including small amount of chloroform to yield a crude mixture of 2,7,10,15-tetrabromodibenzo[*g*,*p*]chrysene (348 mg) as white powder.

Using the crude mixture of 2,7,10,15-tetrabromodibenzo[*g*,*p*]chrysene, 2,7,10,15-tetrakis(4,4,5,5-tetramethyl-1,3,2-dioxaborolan-2-yl)dibenzo[*g*,*p*]chrysene was prepared by a reported procedure with minor change as follows[4]. The portion of crude mixture of 2,7,10,15-tetrabromodibenzo[*g*,*p*]chrysene (200 mg), bis(pinacolato)diboron (394 mg, 1.55 mmol), [1,1'-bis(diphenylphosphino)ferrocene] palladium(II) dichloride dichloromethane adduct (25.4 mg, 0.0311 mmol), and potassium acetate (213 mg, 2.17 mmol) were dissolved in 1,4-dioxane (7.0 mL). The solution was stirred at 110°C for 18 h under a nitrogen atmosphere. After cooling to room temperature, water was added to the reaction solution and extracted with ethyl acetate. The organic phase was washed with water, dried with Na_2SO_4, and evaporated under reduced pressure. The residue was purified by passing a short column chromatography (silica gel; ethyl acetate/hexane (20:80 v/v) as the eluent) to give crude mixture of 2,7,10,15-tetrakis(4,4,5,5-tetramethyl-1,3,2-dioxaborolan-2-yl)dibenzo[*g*,*p*]chrysene (150 mg). HRMS-MALDI (*m*/*z*): $[M]^+$ calcd. for $C_{50}H_{60}B_4O_8$, 832.468; Found 832.465.

The crude mixture of 2,7,10,15-tetrakis(4,4,5,5-tetramethyl-1,3,2-dioxaborolan-2-yl)dibenzo[*g*,*p*]chrysene (150 mg), 1-bromo-8-iodonaphthalene (300 mg, 0.901 mmol), potassium carbonate (137 mg, 0.991 mmol), and tetrakis(triphenylphosphine) palladium(0) (16.7 mg, 0.0144 mmol) in anhydrous *N*,*N*-dimethylformamide (6.3 mL) was reacted at 110 °C for 12 h under a nitrogen atmosphere. After completion of the reaction, chloroform was added to the reaction solution and washed with water three times. The organic layer was dried by Na_2SO_4 and concentrated under reduced pressure to give a crude material. The crude material was purified through column chromatograph (silica gel; dichloromethane/hexane (20:80 v/v) as the eluent) to yield 2,7,10,15-tetrakis(8-bromonaphthalen-1-yl)dibenzo[*g*,*p*]chrysene as a pale-yellow powder (62.5 mg). The yield after three step reactions was 15.6% with consideration of a decrease in the amount of intermediate crude material during the steps. ^{1}H NMR ($CDCl_3$, 500 MHz): δ = 8.91–8.86 (m, 4H), 8.70–8.68 (m, 4H), 7.93–7.90 (m, 8H), 7.83–7.78 (m, 4H), 7.69 (d, J = 8.5 Hz, 4H), 7.66–7.60 (m, 4H), 7.57–7.53 (m, 4H), 7.33–7.29 (m, 4H) (Supplementary Fig. 5a). ^{13}C NMR ($CDCl_3$, 125 MHz): δ = 141.10, 140.35, 136.15, 133.85, 131.69, 130.39, 129.80, 129.08, 128.97, 128.61, 128.03, 127.68, 126.14, 125.39, 125.01, 120.46, 120.32 (Supplementary Fig. 5b). HRMS-MALDI (*m*/*z*): $[M]^+$ calcd. for $C_{66}H_{36}Br_4$, 1147.952; Found 1147.949 (Supplementary Fig. 5c).

2,7,10,15-Tetrakis(8-(phenylselanyl)naphthalen-1-yl)dibenzo[*g*,*p*]chrysene (5):
2,7,10,15-Tetrakis(8-bromonaphthalen-1-yl)dibenzo[*g*,*p*]chrysene (55.0 mg, 0.0479 mmol), 1,2-diphenyldiselane (44.8 mg, 0.143 mmol), [1,1′-bis(diphenylphosphino)ferrocene]dichloropalladium(II) (3.50 mg, 0.00479 mmol), and Zinc (11.0 mg, 0.168 mmol) were added to anhydrous tetrahydrofuran (2.2 mL). The mixture solution was reacted at a reflux temperature for 18 h under a nitrogen atmosphere. Dichloromethane was added to the reaction solution and washed with water three times. The organic phase was dried with Na_2SO_4, evaporated under reduced pressure, purified via column chromatography (silica gel; dichloromethane/hexane (35:65 v/v) as the eluent) to obtain **5** as pale-yellow powder (37.5 mg, 53.9%). ^{1}H NMR (acetone-d_6, 500 MHz): δ = 9.04–8.98 (m, 4H), 8,94–8.90 (m, 2H), 8.79–8.75 (m, 2H), 8.04–8.00 (m, 4H), 7.93–7.86 (m, 8H), 7.71–7.60 (m, 8H), 7.33–7.11 (m, 28H) (Supplementary Fig. 6a). ^{13}C NMR ($CDCl_3$, 125 MHz): δ = 140.51, 135.76, 135.28, 135.11, 133.74, 133.38, 132.82, 132.08, 131.86, 131.21, 130.68, 130.29, 129.18, 129.09, 128.82, 127.70, 127.42, 127.13, 125.99, 125.77, 125.05 (Supplementary Fig. 6b). HRMS-MALDI (*m*/*z*): $[M]^+$ calcd. for $C_{90}H_{56}Se_4$, 1454.109; Found 1454.113 (Supplementary Fig. 6c). Anal. Calcd. for $C_{90}H_{56}Se_4$: C, 74.38; H, 3.88; Se, 21.74. Found C, 74.15; H, 3.58.

Naphthalene-*d*$_6$-1,8-diamine:
Naphthalene-1,8-diamine (1.00 g, 6.32 mmol), 10 wt% platinum on active carbon (250 mg), and D_2O (25 mL) in a 50 ml Teflon-lined autoclave was heated at 150 °C for 12 h. After cooling to room temperature, ethyl acetate was added, and undissolved solid was removed by filtration. After extraction with ethyl acetate, organic phase was washed with water three times, dried over Na_2SO_4, and concentrated under reduced pressure. The residue was purified through a short column (silica gel; ethyl acetate/hexane (22:78 v/v) as the eluent) to obtain brown solid (837 mg). By using the brown solid as starting material, the same procedure was performed to give a naphthalene-*d*$_6$-1,8-diamine as brown solid (439 mg, 59.5%). The deuteration yield was determined to be 80.8% by comparing the ^{1}H NMR signal intensities of naphthalene-*d*$_6$-1,8-diamine and that of naphthalene-1,8-diamine (Supplementary Fig. 7a). ^{13}C NMR (acetone-d_6, 125 MHz): δ = 146.84, 146.78, 146.72, 138.04, 126.76, 126.66, 126.58, 118.93, 118.74, 118.54, 117.72, 111.35 (Supplementary Fig. 7b). HRMS-MALDI (*m*/*z*): $[M]^+$ calcd. for $C_{10}H_4D_6N_2$, 164.122; Found 163.117 (Supplementary Fig. 7c).

1,8-Dibromonaphthalene-*d*$_6$:

Sodium nitrite (423 mg, 6.13 mmol) was slowly added to the concentrated sulfuric acid (4.2 mL) at 0 °C under stirring. A solution of acetic acid (4.2 mL) dissolving naphthalene-d_6-1,8-diamine (438 mg, 2.67 mmol) was added dropwise over a period of 30 min at 0 °C and then stirred for 1 h. Urea saturated water (0.14 mL) was added to decompose free HNO_2. A solution of cupper(I)bromide (1.05 g, 7.33 mmol) in aqueous hydrogen bromide (48%) (16 mL) was added to the reaction solution at 0 °C and stirred at room temperature for 20 h. After toluene was added, the reaction solution was filtered with suction and washed with dichloromethane. After adding water to the filtrate, organic layer was extracted with dichloromethane, washed with pure water three times, and dried over Na_2SO_4. The organic phase was concentrated by reduced pressure and purified by column chromatography (silica gel; dichloromethane/hexane (10:90 v/v) as the eluent) to give a 1,8-dibromonaphthalene-d_6 as white crystalline powder (229 mg, 29.4%). The deuteration yield was determined to be 76.9% by comparing the ^{1}H NMR signal intensities of 1,8-dibromonaphthalene-d_6 and that of 1,8-dibromonaphthalene (Supplementary Fig. 8a). ^{13}C NMR ($CDCl_3$, 125 MHz): δ = 137.06, 135.40, 135.32, 135.20, 129.48, 129.36, 129.28, 128.98, 126.34, 126.23, 125.95, 119.60, 119.49 (Supplementary Fig. 8b).

1-Bromo-8-iodonaphthalene-*d*$_6$:

1,8-Dibromonaphthalene-d_6 (200 mg, 0.685 mmol) was dissolved in anhydrous tetrahydrofuran (15 mL) under a nitrogen atmosphere and cooled down at –78 °C. A solution of *n*-BuLi (1.6 M in hexane) (0.43 mL, 0.69 mmol) was added dropwise to the mixture solution under stirring. After 5 min, a solution of anhydrous tetrahydrofuran (0.8 mL) containing iodine (174 mg, 0.685 mmol) was added slowly to the reaction solution. The reaction solution gradually warmed to room temperature and stirred for 22 h. The reaction was quenched by adding an aqueous solution of thiosulfate (1.6 M) (10 mL). The mixture was extracted with dichloromethane and washed with water three times. Organic phase was dried over Na_2SO_4, concentrated under reduced pressure to give a crude material. The crude material was purified through column chromatography (silica gel; dichloromethane/hexane (7:93 v/v) as the eluent) to obtain 1-bromo-8-iodonaphthalene-d_6 as a pale-yellow crystalline solid (164 mg, 70.4%). The deuteration yield was determined to be 77.6% by comparing the ^{1}H NMR signal intensities of 1-bromo-8-iodonaphthalene-d_6 and that of 1-bromo-8-iodonaphthalene (Supplementary Fig. 9a). ^{13}C NMR ($CDCl_3$, 125 MHz): δ = 144.36, 144.28, 136.31, 135.04, 134.95, 130.36, 130.26, 129.88, 126.97, 126.85, 126.14, 126.03, 121.55, 121.45, 91.03, 90.88 (Supplementary Fig. 9b).

2,7,10,15-Tetrakis(8-bromonaphthalen-1-yl)dibenzo[*g*,*p*]chrysene-d_{36}

Dibenzo[*g*.*p*]chrysene-d_{16} was prepared by previously reported procedure[3]. Dibenzo[*g*.*p*]chrysene-d_{16} (70.0 mg, 0.203 mmol) was dissolved in anhydrous chlorobenzene (3.7 mL). Bromine (169 mg, 1.06 mmol) diluted in anhydrous chlorobenzene (0.54 mL) was added slowly to the mixture and stirred for 16 h at 110 °C under a nitrogen atmosphere. After completion of the reaction, chloroform was added to the reaction mixture and washed with pure water three times. The organic phase was dried over Na_2SO_4, evaporated under low pressure, and washed with hexane including small amount of chloroform with suction to yield a crude mixture of 2,7,10,15-tetrabromodibenzo[*g*,*p*]chrysene-d_{12} as a white powder (132 mg). HRMS-MALDI (*m*/*z*): $[M]^+$ calcd. for $C_{26}D_{12}Br_4$, 655.838; Found 655.836.

The portion of crude mixture of 2,7,10,15-tetrabromodibenzo[*g*,*p*]chrysene-d_{12} (125 mg), [1,1'-bis(diphenylphosphino)ferrocene]palladium(II) dichloride dichloromethane adduct (15.6 mg, 0.0191 mmol), and potassium acetate (131 mg, 1.33 mmol) were dissolved in 1,4-dioxane (4.3 mL). The mixture was heated at 110°C for 20 h under a nitrogen atmosphere. After completion of the reaction, ethyl acetate was added and washed with water three times. The organic layer was dried by Na_2SO_4, evaporated under vacuum. The residue was purified by passing a short column chromatography (silica gel; ethyl acetate/hexane (20:80 v/v) as the eluent) to give crude mixture of 2,7,10,15-tetrakis(4,4,5,5-tetramethyl-1,3,2-dioxaborolan-2-yl)dibenzo[*g*,*p*]chrysene-d_{12} as pale-yellow powder (92.0 mg). HRMS-MALDI (*m*/*z*): $[M]^+$ calcd. for $C_{50}H_{48}D_{12}B_4O_8$, 844.544; Found 844.552.

The portion of crude mixture of 2,7,10,15-tetrakis(4,4,5,5-tetramethyl-1,3,2-dioxaborolan-2-yl)dibenzo[*g*,*p*]chrysene-d_{12} (88.0 mg), 1-bromo-8-iodonaphthalene (160 mg, 0.469 mmol), potassium carbonate (79.2 mg, 0.573 mmol), and tetrakis(triphenylphosphine)palladium(0) (9.63 mg, 0.00834 mmol) in anhydrous *N*,*N*-dimethylformamide (2.5 mL) was stirred at 110 °C for 27 h under a nitrogen atmosphere. After cooling to room temperature, water was added and extracted with dichloromethane. The organic phase was washed with water three times, dried over Na_2SO_4, and evaporated under reduced pressure to give a crude material. The crude material was purified via column chromatograph (silica gel; dichloromethane/hexane (20:80 v/v) as the eluent) to produce 2,7,10,15-tetrakis(8-bromonaphthalen-1-yl)dibenzo[*g*,*p*]chrysene-d_{36} as a pale-yellow powder (49.6 mg). The yield after three step reactions was 22.7% with consideration of a decrease in the amount of intermediate crude material during the steps. The deuteration yield was determined to be 85.8% by comparing the ^1H NMR signal

intensities of 2,7,10,15-tetrakis(8-bromonaphthalen-1-yl)dibenzo[*g*,*p*]chrysene-d_{36} and that of 2,7,10,15-tetrakis(8-bromonaphthalen-1-yl)dibenzo[*g*,*p*]chrysene (Supplementary Fig. 10a). ^{13}C NMR ($CDCl_3$, 125 MHz): δ = 141.01, 140.89, 140.20, 140.15, 136.04, 135.97, 133.74, 133.74, 131.58, 131.55, 130.28, 129.71, 129.69, 128.96, 128.80, 128.80, 128.70, 128.50, 128.50, 127.64, 127.48, 126.03, 125.88, 125.28, 125.16, 124.70, 120.35, 120.27, 120.21, 120.16 (Supplementary Fig. 10b). HRMS-MALDI (*m*/*z*): $[M]^+$ calcd. for $C_{66}D_{36}Br_4$, 1184.178; Found 1179.151 (Supplementary Fig. 10c).

2,7,10,15-Tetrakis(8-(phenylselanyl)naphthalen-1-yl)dibenzo[*g*,*p*]chrysene-d_{36} (5d)
2,7,10,15-Tetrakis(8-bromonaphthalen-1-yl)dibenzo[*g*,*p*]chrysene-d_{36} (40.0 mg, 0.0338 mmol), 1,2-diphenyldiselane (42.2 mg, 0.135 mmol), [1,1′-bis(diphenylphosphino) ferrocene]dichloropalladium(II) (3.70 mg, 0.00506 mmol), and Zinc (11.0 mg, 0.168 mmol) were added to anhydrous tetrahydrofuran (1.6 mL). The mixture solution was stirred at a reflux condition for 26 h under a nitrogen atmosphere. After completion of the reaction, dichloromethane was added to the reaction solution and washed with water three times. The organic phase was dried over Na_2SO_4 and evaporated under reduced pressure to give a crude material. The crude material was purified by column chromatography (silica gel; dichloromethane/hexane (33:67 v/v) as the eluent) to produce **5d** as pale-yellow powder (22.8 mg, 45.3%). The deuteration yield was determined to be 86.1% by comparing the ^{1}H NMR signal intensities of **5d** and that of **5** (Supplementary Fig. 11a). ^{13}C NMR ($CDCl_3$, 125 MHz): δ = 140.39, 135.78, 135.31, 135.12, 133.79, 133.38, 132.76, 132.02, 131.78, 131.49, 130.57, 130.20, 129.21, 129.15, 128.95, 127.71, 125.86, 125.77, 124.80 (Supplementary Fig. 11b). HRMS-MALDI (*m*/*z*): $[M]^+$ calcd. for $C_{90}H_{20}D_{36}Se_4$, 1490.335; Found 1484.343 (Supplementary Fig. 11c).

(*S*)-2,2'-bis(diphenylphosphaneyl)-1,1'-binaphthalene-d_{32} ((*S*)-BINAP-d_{32})
(*S*)-[1,1'-Binaphthalene]-2,2'-diylbis[1,1-diphenyl-1,1'-phosphine oxide] (123 mg, 0.188 mmol) was treated with 10 % Pd on active carbon (125 mg) and D_2O (25 mL) in a 50 mL Teflon-lined autoclave at 250 °C for 12 h. The internal pressure reached 4–5 MPa. After cooling to room temperature, ethyl acetate was added. The organic phase was washed with water three times and dried over Na_2SO_4. After filtration, the organic phase was concentrated under reduced pressure to give a crude material. The crude material was purified through a short column chromatography (silica gel; ethyl acetate) to give white solid (37.3 mg). The white solid (29.3 mg) were dissolved in 1,1,3,3-tetramethyldisiloxane (TMDS) (0.24 mL, 1.4 mmol), tetraisopropyl orthotitanate (0.01 mL, 0.04 mmol) and toluene (0.25 mL). The mixture was heated at 80 °C for 25 h under

a nitrogen atmosphere. Additional TMDS (0.2 mL) was injected, and the reaction was continued for 18 h. The organic phase was washed with water three times and dried over Na_2SO_4 and then filtered. The filtrate was concentrated under reduced pressure. The residue was purified by column chromatography (silica gel; dichloromethane/hexane (20:80 v/v) as the eluent) to give (*S*)-BINAP-d_{32} (19.5 mg). The yield after step reactions was 20.2% with consideration of a decrease in the amount of intermediate material during the step. The deuteration yield was determined to be 91.4% by comparing the ^{1}H NMR signal intensities of (*S*)-BINAP and that of (*S*)-BINAP-d_{32} (Supplementary Fig. 12a). ^{13}C NMR ($CDCl_3$, 125 MHz): δ = 145.44–145.11 (m), 137.83–137.72 (m), 137.34–137.23 (m), 135.48 (d, $J_{C–P}$ = 7.5 Hz), 134.19–134.02 (m), 133.43 (t, $J_{C–P}$ = 5.0 Hz), 133.11, 132.86–132.68 (m), 130.55–130.42 (m), 128.05–127.05 (m), 125.99 (d, $J_{C–P}$ = 5.0 Hz, 77.5 Hz) (Supplementary Fig. 12b). HRMS-MALDI (*m*/*z*): $[M]^+$ calcd. for $C_{44}D_{32}P_2$, 654.398; Found 653.394 (Supplementary Fig. 12c).

2,7,10,15-tetrakis(8-(phenylthio)naphthalen-1-yl)dibenzo[*g,p*]chrysene (R1):

2,7,10,15-Tetrakis(8-bromonaphthalen-1-yl)dibenzo[*g,p*]chrysene (139.1 mg, 0.121 mmol), diphenyl disulfide (117.6 mg, 0.539 mmol), [1,1′-bis(diphenylphosphino)ferrocene]dichloropalladium(II) (12.3 mg, 0.0168 mmol), and Zinc (41.2 mg, 0.630 mmol) in anhydrous tetrahydrofuran (4.0 mL) was stirred at a reflux temperature for 24 h under a nitrogen atmosphere. After completion of the reaction, dichloromethane was added to the reaction solution and washed with brine three times. The organic phase was dried over anhydrous Na_2SO_4, evaporated under a reduced pressure, purified via column chromatography (silica gel; dichloromethane/hexane (35:65 v/v) as the eluent) to obtain **R1** as pale-yellow powder (79.6 mg, 51.9%). ^{1}H NMR (acetone-d_6, 500 MHz): δ = 8.83–8.71 (m, 4H), 8.66 (s, 1H), 8.38–8.33 (m, 2H), 8.09–7.96 (m, 8H), 7.70–7.54 (m, 14H), 7.50–7.39 (m, 7H), 7.10–6.82 (m, 15H), 6.74–6.67 (m, 5H) (Supplementary Fig. 13a). ^{13}C NMR ($CDCl_3$, 125 MHz): δ = 141.43, 141.35, 140.55, 140.48, 140.42, 140.34, 140.29, 138.73, 138.48, 138.14, 137.91, 137.83, 137.68, 135.53, 135.27, 134.78, 134.37, 134.15, 133.96, 133.86, 133.58, 133.41, 133.18, 133.12, 133.02, 132.61, 132.49, 132.06,131.83, 131.68, 131.53, 131.38, 131.30, 131.22, 131.17, 131.12, 131.09, 131.06, 130.55, 130.50, 130.46, 130.39, 130.28, 129.96, 129.84, 129.58, 129.49, 129.14, 129.09, 128.93, 128.90, 128.86, 128.76, 128.64, 128.55, 128.51, 128.39, 128.32, 128.09, 127.94, 127.84, 127.76, 127.62, 127.53, 127.48, 126.69, 126.54, 126.34, 125.95, 125.85, 125.83, 125.78, 125.16, 124.71, 124.66, 124.59 (Supplementary Fig. 13b). HRMS-MALDI (*m*/*z*): $[M]^+$ calcd. for $C_{90}H_{56}S_4$, 1264.325; Found 1264.326 (Supplementary Fig. 13c).

Dibenzo[*g,p*]chrysene-3-yl(phenyl)selane (R2):

9-Fluorenone (1.35 g, 7.50 mmol), 2-bromo-9-fluorenone (648 mg, 2.50 mmol), zinc (4.21 g, 64.4 mmol), and zinc chloride (2.73 g, 20.0 mmol) in tetrahydrofuran (25 ml) and pure water (25 ml) was stirred at room temperature for 4 h. After completion of the reaction, dichloromethane was added to the reaction solution and filtrated to remove the undissolved solid. Pure water was added to the solution and extracted with dichloromethane. The organic layer was dried over anhydrous Na_2SO_4 and evaporated under reduced pressure to give a white powder (2.09 g). The powder (2.09 g) in acetic acid (35.5 ml) and sulfuric acid (1.8 ml) was heated at 85 °C for 4 h. After cooling to room temperature, dichloromethane was added to the reaction mixture and washed with pure water and extracted with dichloromethane. The organic layer was dried over anhydrous Na_2SO_4 and concentrated under reduced pressure. The residue was purified by column chromatography (silica gel; dichloromethane: hexane = 3: 7) to give $C_{26}H_{16}BrO$ (430 mg) as a white powder. Although the products of $C_{26}H_{16}BrO$ had potentially three kinds of isomers, the products were directly used for the next reaction without further purification. $C_{26}H_{16}BrO$ (430 mg, 1.02 mmol), sodium borohydride (67.3 mg, 1.78 mmol) in anhydrous tetrahydrofuran (15 ml) and methanol (15 ml) was stirred at 60 °C for 3 h under a nitrogen atmosphere. After completion of the rection, dichloromethane was added to the solution and washed with pure water. The organic layer was dried over anhydrous Na_2SO_4 and concentrated under reduced pressure to give a white powder (391 mg). The powder (391 mg) in acetic acid (28 ml) and sulfuric acid (0.70 ml) was heated at 85 °C for 4 h. After cooling to room temperature, pure water was added to the reaction solution and extracted with dichloromethane. The extracted organic layer was dried over anhydrous Na_2SO_4 and evaporated under reduced pressure. The residue was purified by column chromatography (silica gel; dichloromethane: hexane = 1: 9) to yield 3-bromodibenzo[*g,p*]chrysene (212 mg) as a white powder. The reaction yield of 3-bromodibenzo[*g,p*]chrysene from 2-bromo-9-fluorenone was 20.8%. 3-Bromodibenzo[*g,p*]chrysene (142 mg, 0.349 mmol), diphenyl diselenide (172 mg. 0.552 mmol), [1,1'-bis(diphenylphosphino)ferrocene]palladium(II) dichloride (40.4 mg, 0.0552 mmol), and zinc (72.2 mg, 1.10 mmol) in anhydrous tetrahydrofuran (11 ml) was stirred at reflux tem,perature for 16 h under a nitrogen atmosphere. After completion of the reaction, chloroform was added to the reaction solution. The organic phase was washed with pure water three times, dried over anhydrous Na_2SO_4, and concentrated under the reduced pressure to give a crude product. The crude product was purified by column chromatography (silica gel; dichloromethane: hexane = 12: 88) to give **R2** as a pale-

yellow powder (73.7 mg, 43.7%). Chromophore **R2** was purified further by sublimation. ^{1}H NMR ($CDCl_3$, 500 MHz): δ = 8.71 (sd, *J*= 1.5 Hz, 1H), 8.68–8.63 (m, 5H), 8.58 (d, *J* = 8.5 Hz, 1H), 8.36 (dd, *J* = 8.0, 1.0 Hz, 1H), 7.75 (dd, *J* = 8.5, 1.5 Hz, 1H), 7.69−7.57 (m, 7H), 7.45 (ddd, *J* = 7.5, 6.9, 1.4 Hz, 1H), 7.33−7.29 (m, 3H) (Supplementary Fig. 14a). ^{13}C NMR ($CDCl_3$, 125 MHz): δ = 133.70, 132.69, 131.01, 130.97, 130.78, 130.54, 130.36, 130.15, 129.99, 129.78, 129.51, 129.22, 129.06, 128.98, 128.91, 128.89, 128.47, 128.06, 127.65, 126.76, 126.75, 126.71, 126.69, 126.58, 126.56. 124.38, 123.61, 123.52, 123.45 (Supplementary Fig. 14b). HRMS-MALDI (*m*/*z*): $[M]^+$ calcd. for $C_{32}H_{20}Se$, 484.0730; found 484.0729 (Supplementary Fig. 14c). Anal. Caled. for $C_{32}H_{20}Se$: C, 79.50; H, 4.17; Se, 16.33. Found C, 79.62; H, 4.26.

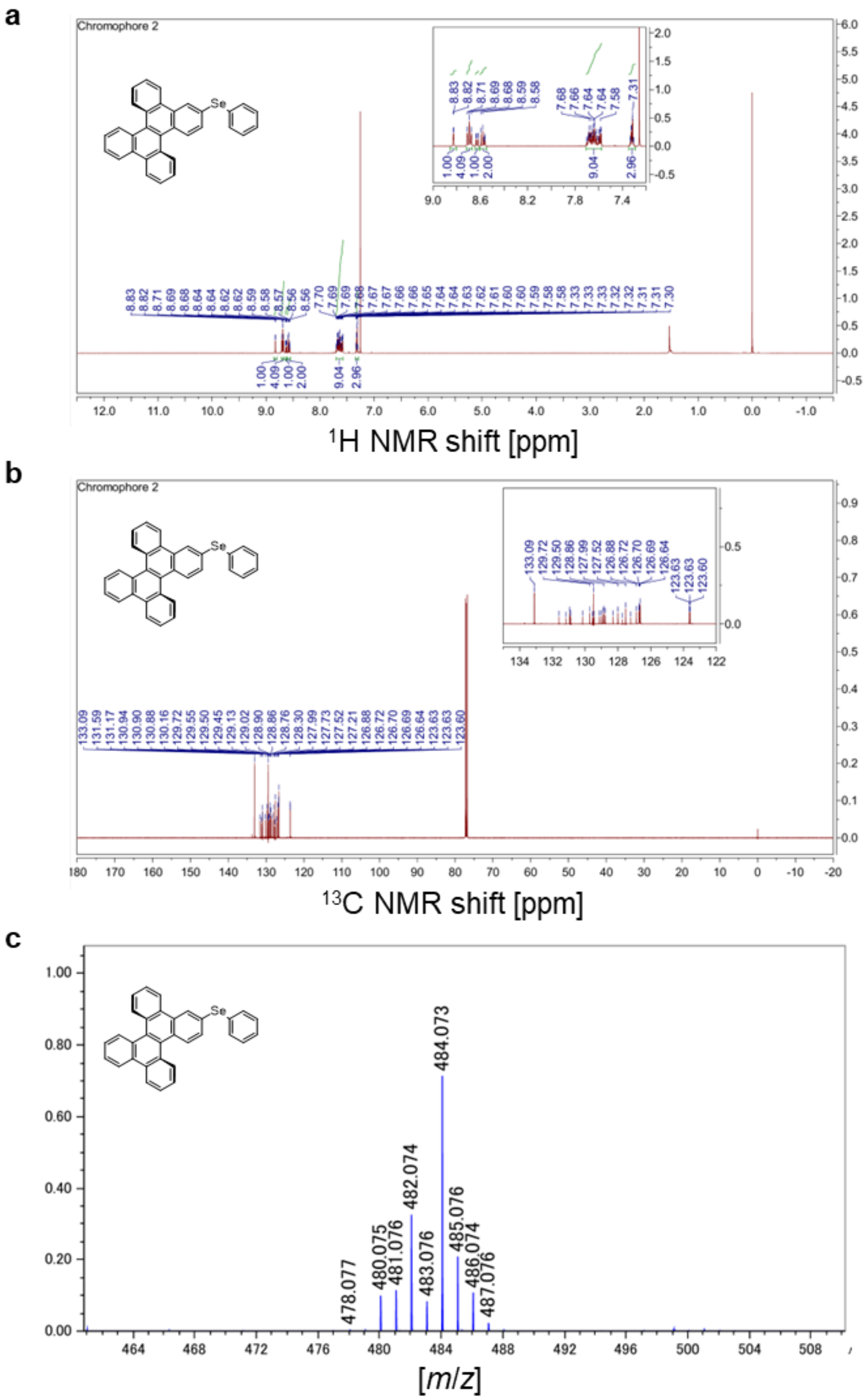

Supplementary Fig. 1. Characterization data of 2. **a**, ^{1}H NMR spectrum in $CDCl_3$. **b**, ^{13}C NMR spectrum in $CDCl_3$. **c**, HRMS-MALDI spectrum.

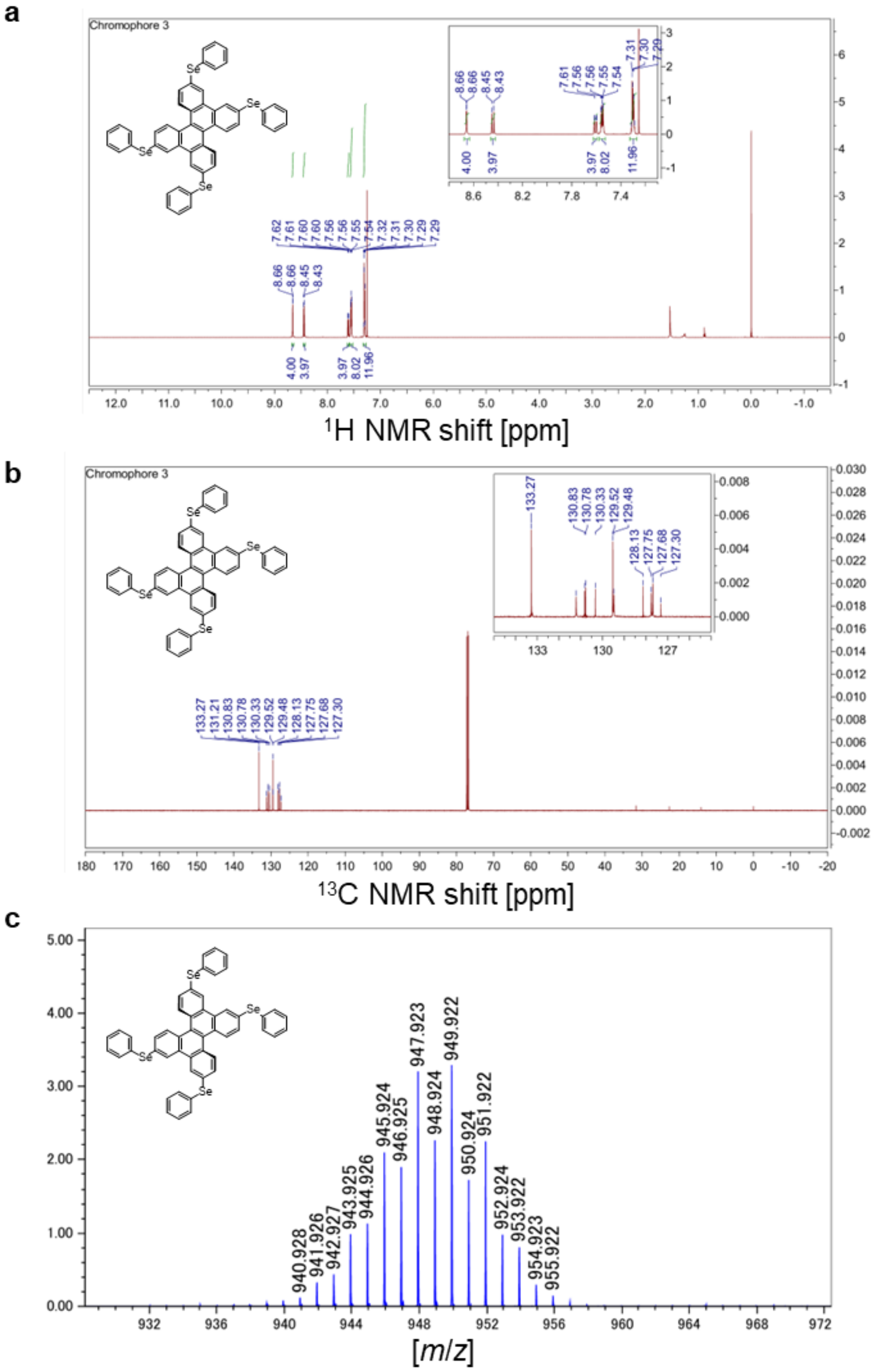

Supplementary Fig. 2. Characterization data of 3. **a**, ^{1}H NMR spectrum in $CDCl_3$. **b**, ^{13}C NMR spectrum in $CDCl_3$. **c**, HRMS-MALDI spectrum.

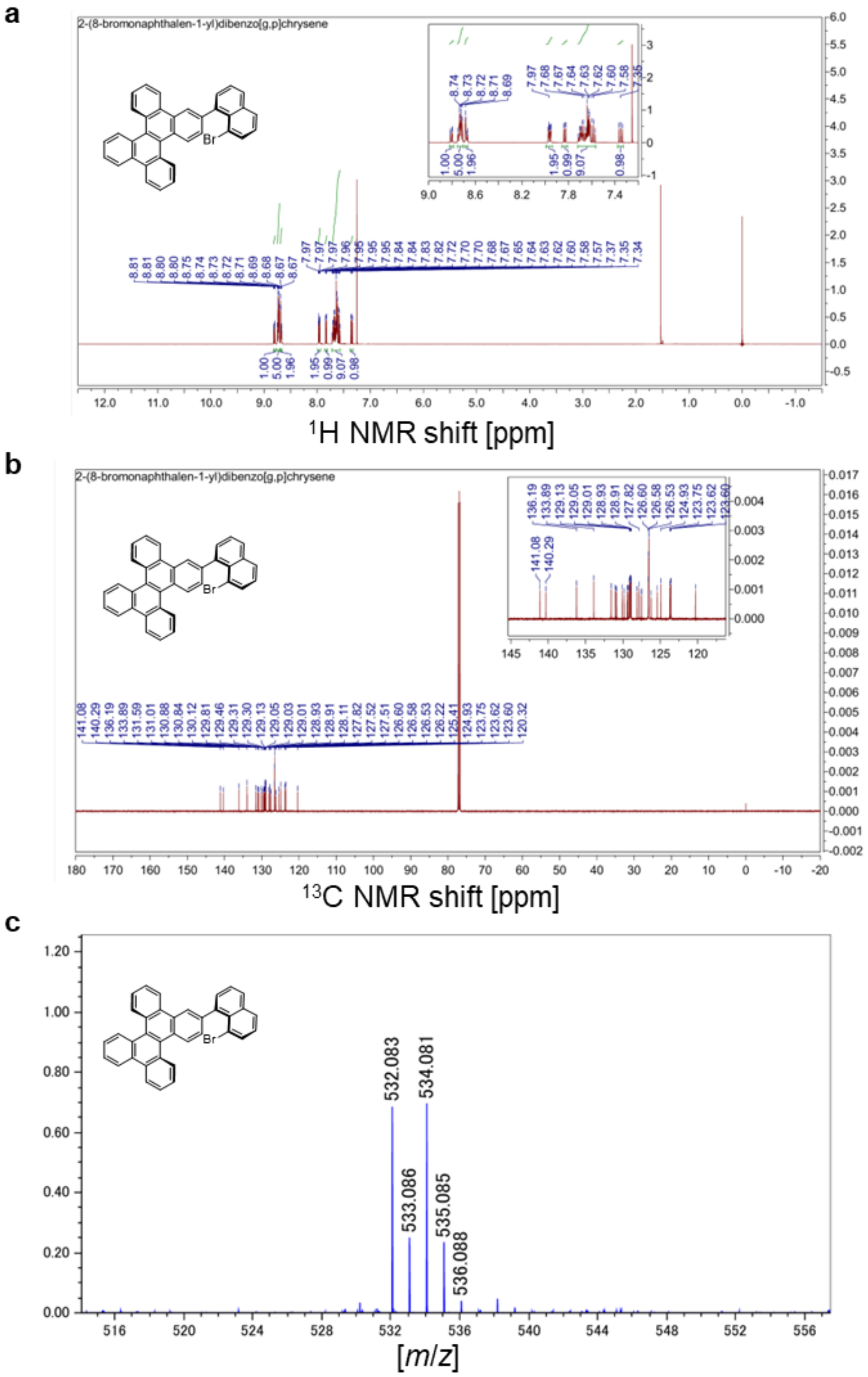

Supplementary Fig. 3. Characterization data of 2-(8-bromonaphthalen-1-yl)dibenzo[*g*,*p*]chrysene. a, ^{1}H NMR spectrum in $CDCl_3$. **b**, ^{13}C NMR spectrum in $CDCl_3$. **c**, HRMS-MALDI spectrum.

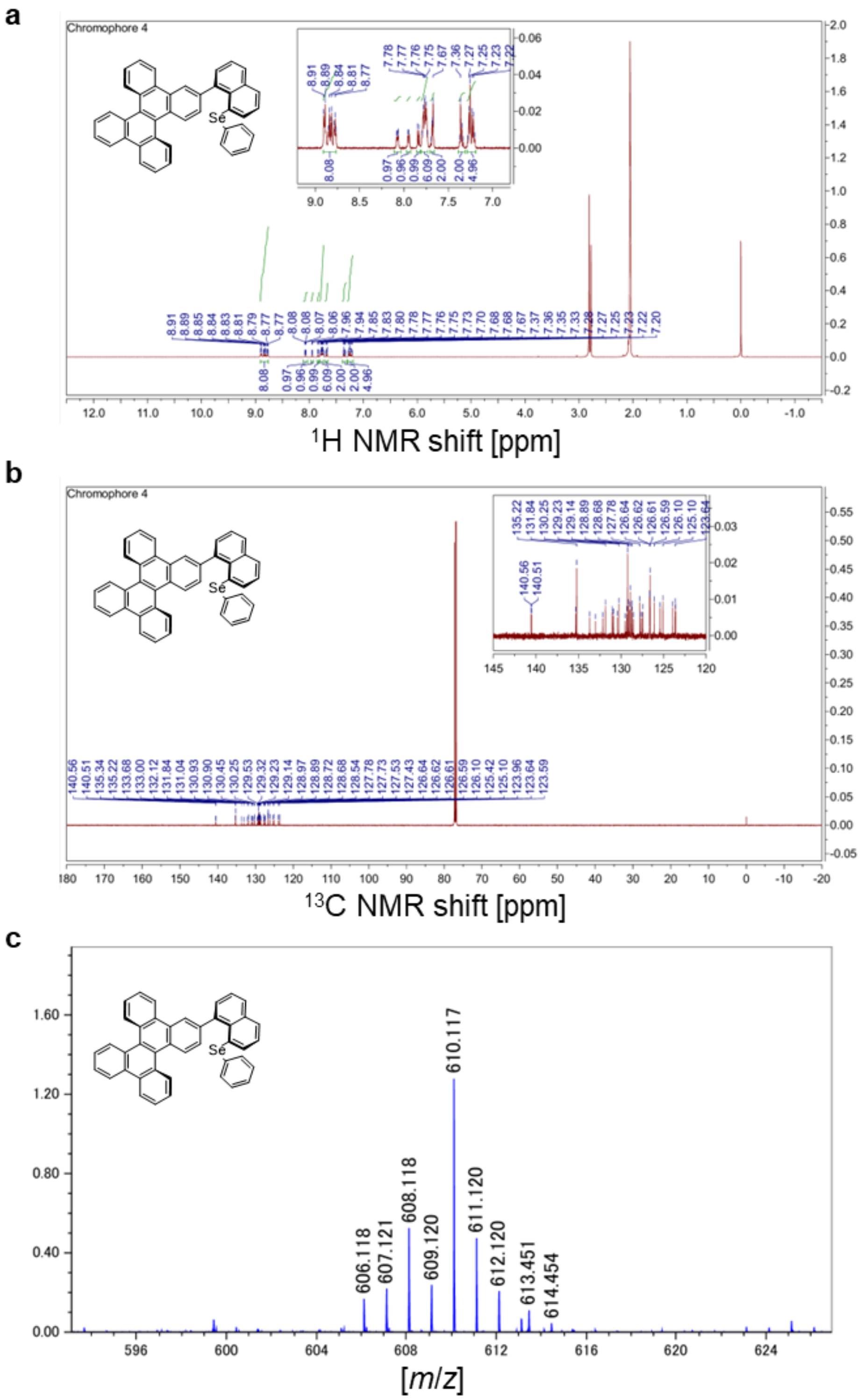

Supplementary Fig. 4. Characterization data of 4. a, ^{1}H NMR spectrum in acetone-d_6. **b**, ^{13}C NMR spectrum in $CDCl_3$. **c**, HRMS-MALDI spectrum.

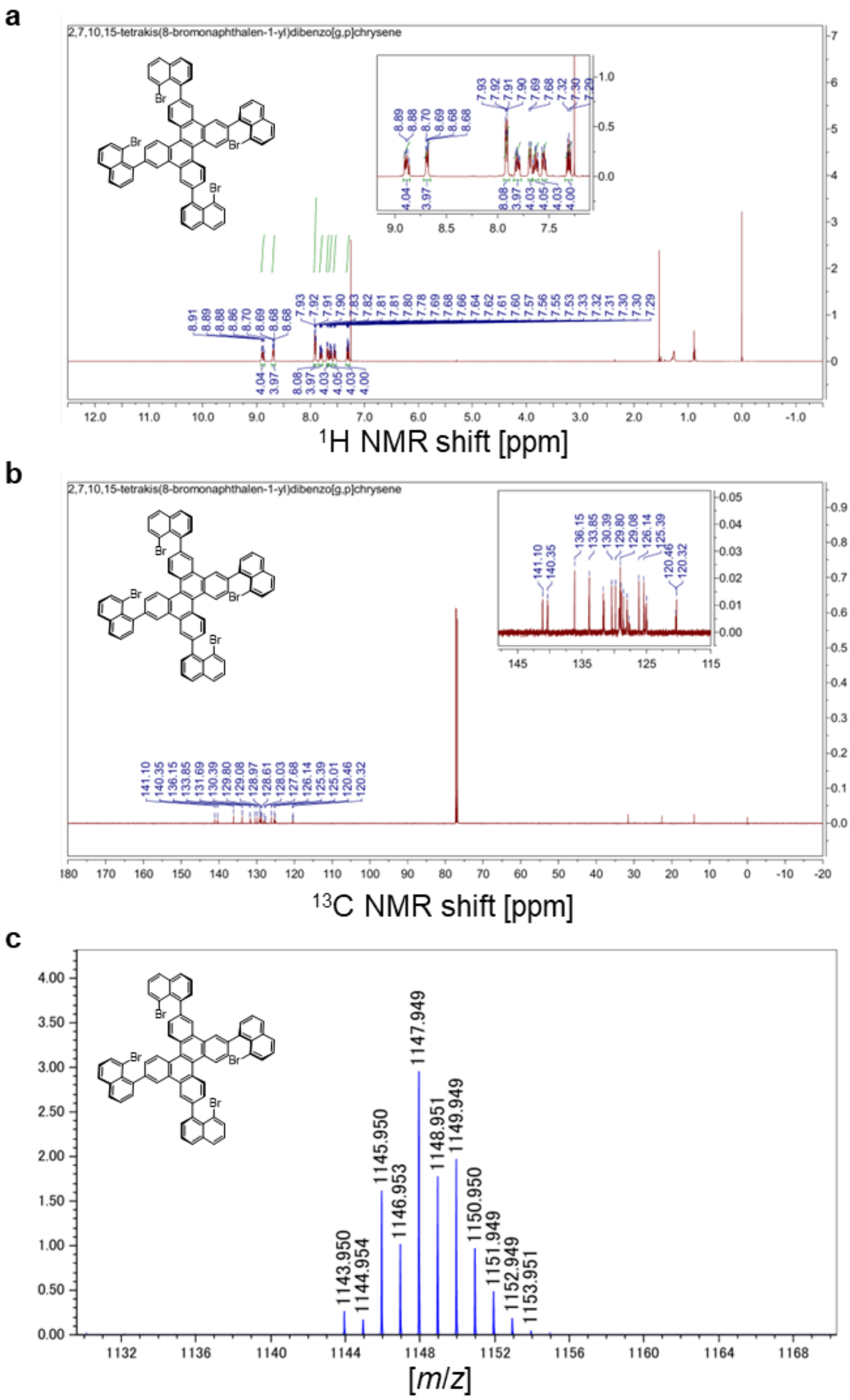

Supplementary Fig. 5. Characterization data of 2,7,10,15-tetrakis(8-bromonaphthalen-1-yl)dibenzo [*g*,*p*]chrysene. a, ^{1}H NMR spectrum in $CDCl_3$. **b**, ^{13}C NMR spectrum in $CDCl_3$. **c**, HRMS-MALDI spectrum.

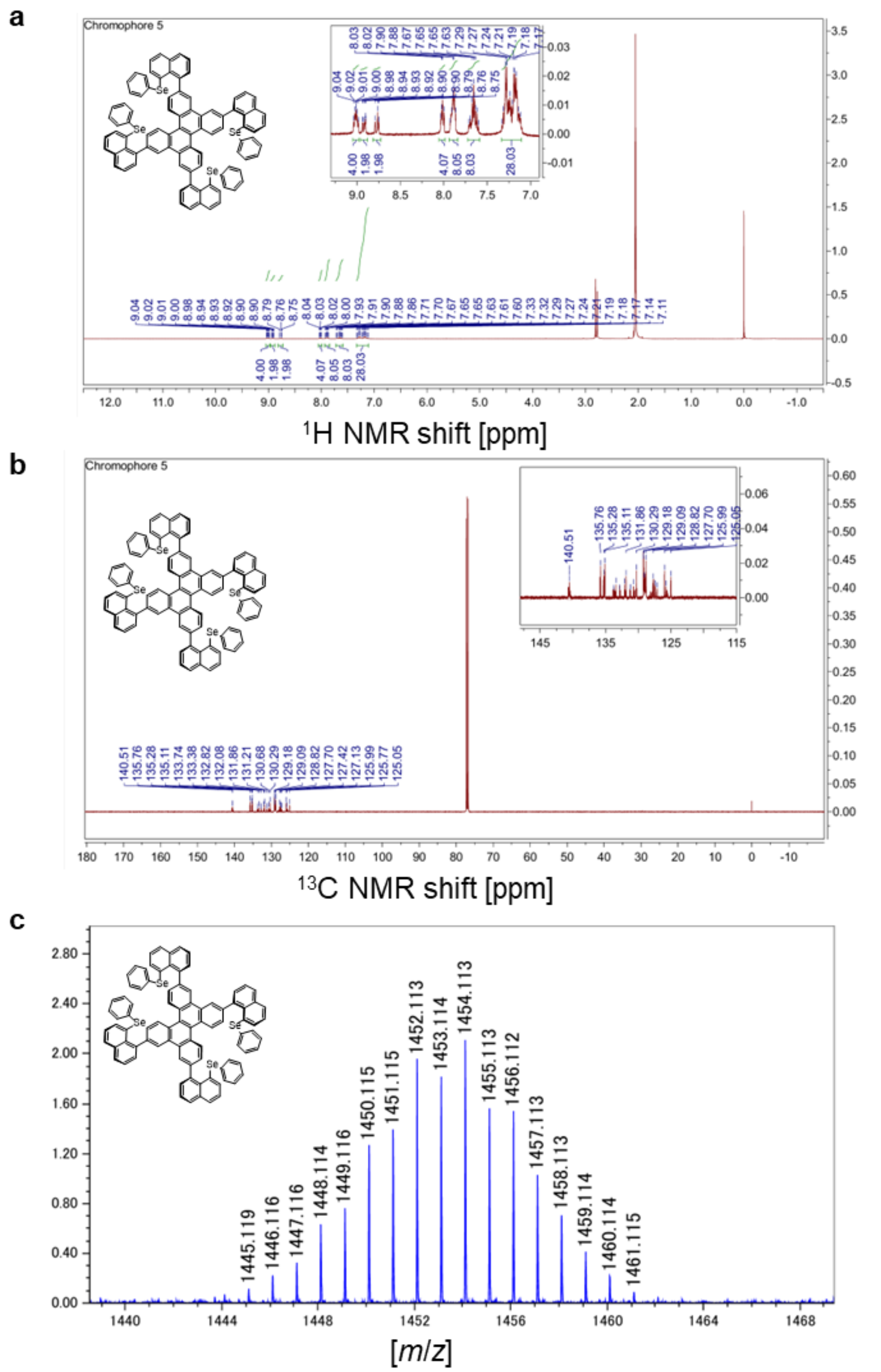

Supplementary Fig. 6. Characterization data of 5. a, ^{1}H NMR spectrum in acetone-d_6. **b**, ^{13}C NMR spectrum in $CDCl_3$. **c**, HRMS-MALDI spectrum.

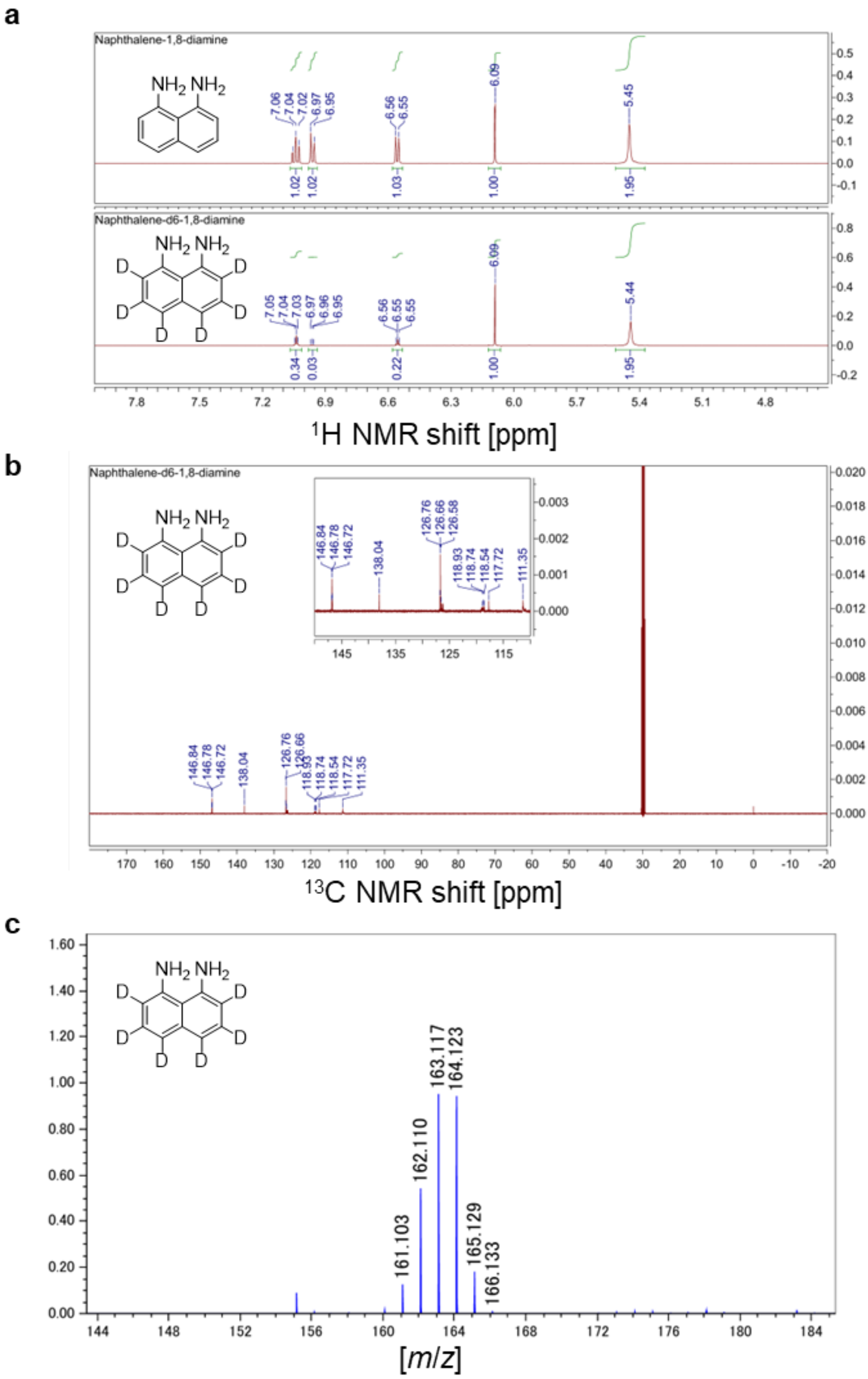

Supplementary Fig. 7. Characterization data of naphthalene-d_6-1,8-diamine. a, ^{1}H NMR spectra of naphthalene-1,8-diamine (top) and naphthalene-d_6-1,8-diamine (bottom) in dimethyl sulfoxide (DMSO)-d_6 containing 1,3,5-trimethoxybenzene. The integral peak intensities were normalized by a signal of 1,3,5-trimethoxybenzene. **b**, ^{13}C NMR spectrum in acetone-d_6. **c**, HRMS-MALDI spectrum.

a

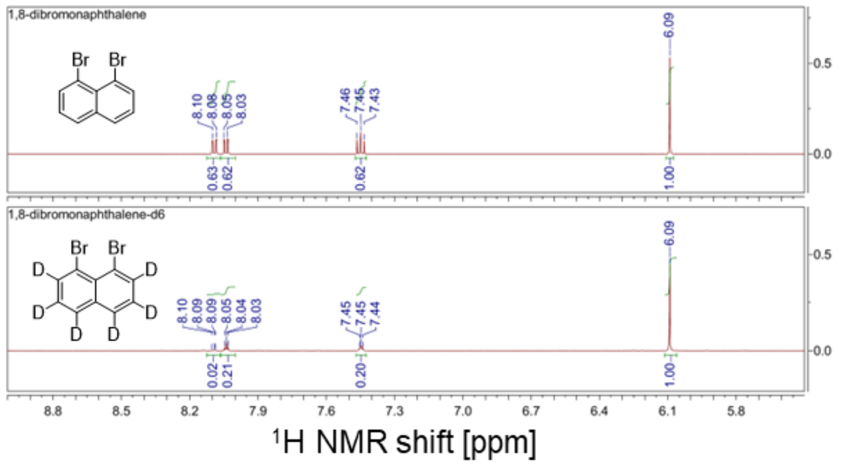

b

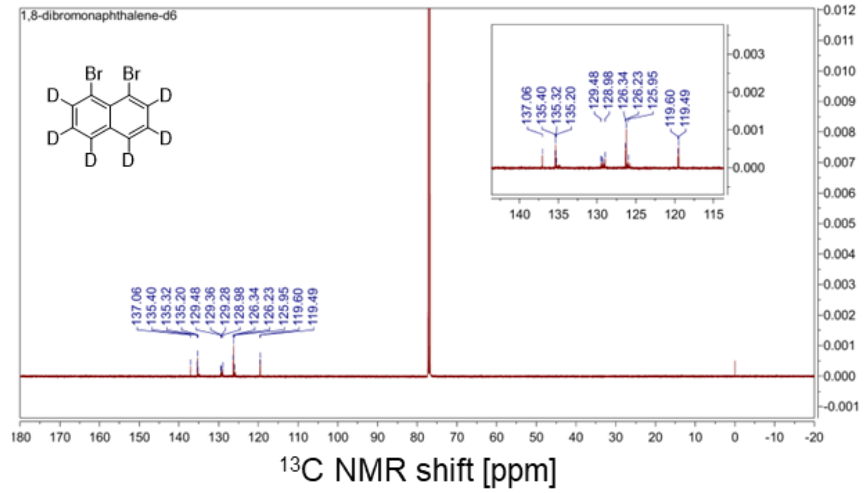

Supplementary Fig. 8. Characterization data of 1,8-dibromonaphthalene-d_6. a, ^{1}H NMR spectra of 1,8-dibromonaphthalene (top) and 1,8-dibromonaphthalene-d_6 (bottom) in DMSO-d_6 containing 1,3,5-trimethoxybenzene. The integral peak intensities were normalized by a signal of 1,3,5-trimethoxybenzene. **b**, ^{13}C NMR spectrum in $CDCl_3$.

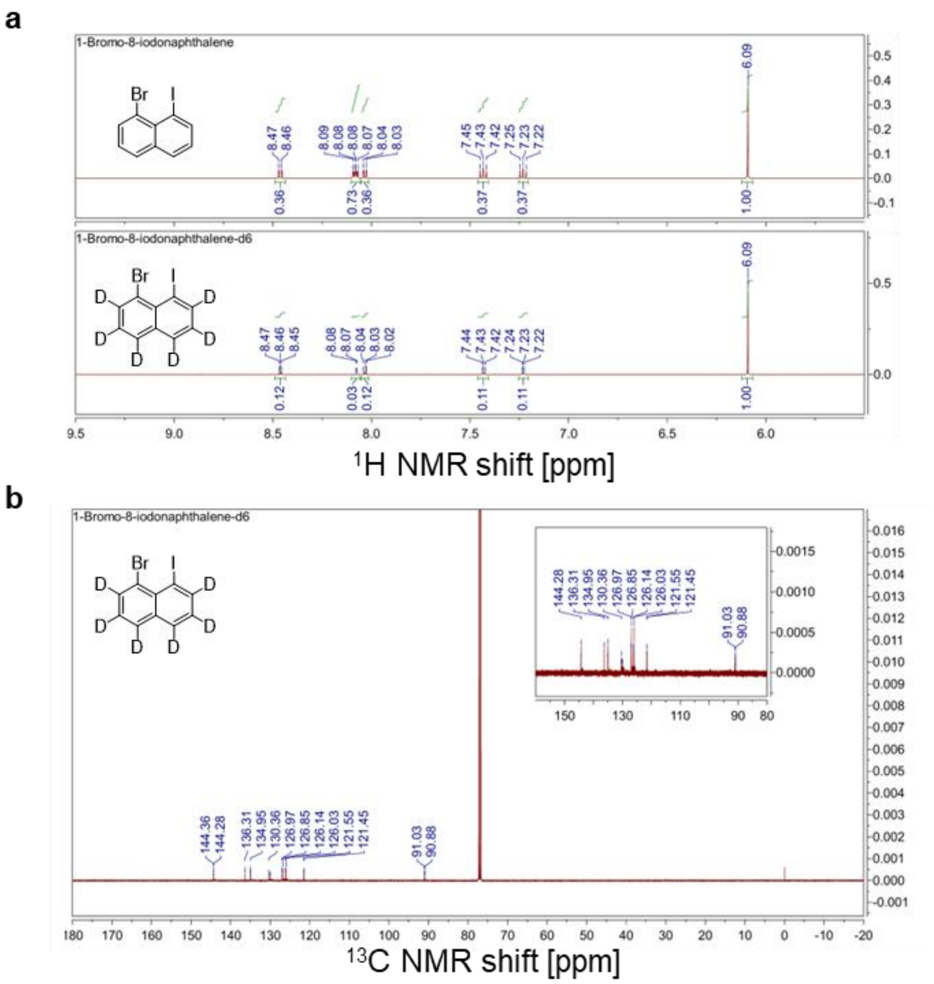

Supplementary Fig. 9. Characterization data of 1-bromo-8-iodonaphthalene-d_6. a, ^{1}H NMR spectra of 1-bromo-8-iodonaphthalene (top) and 1-bromo-8-iodonaphthalene-d_6 (bottom) in DMSO-d_6 containing 1,3,5-trimethoxybenzene. The integral peak intensities were normalized by a signal of 1,3,5-trimethoxybenzene. **b**, ^{13}C NMR spectrum in $CDCl_3$.

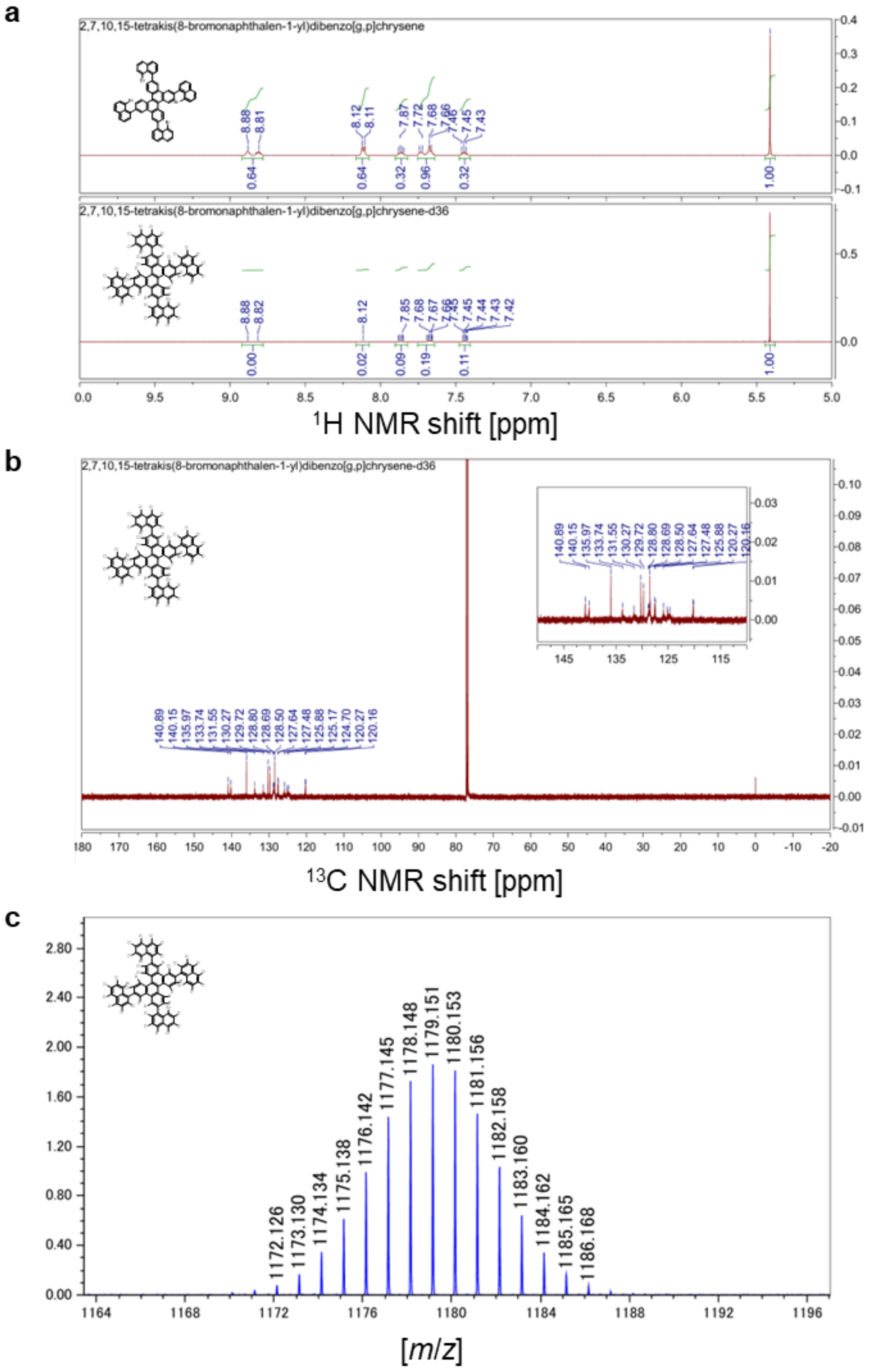

Supplementary Fig. 10. Characterization data of 2,7,10,15-tetrakis(8-bromonaphthalen-1-yl)dibenzo [*g*,*p*]chrysene-*d*$_{36}$. a, ^{1}H NMR spectra of 2,7,10,15-tetrakis(8-bromonaphthalen-1-yl) dibenzo[*g*,*p*]chrysene (top) and 2,7,10,15-tetrakis(8-bromonaphthalen-1-yl)dibenzo [*g*,*p*] chrysene-*d*$_{36}$ (bottom) in DMSO-*d*$_6$ containing dibromomethane. The integral peak intensities were normalized by a signal of dibromomethane. **b**, ^{13}C NMR spectrum in $CDCl_3$. **c**, HRMS-MALDI spectrum.

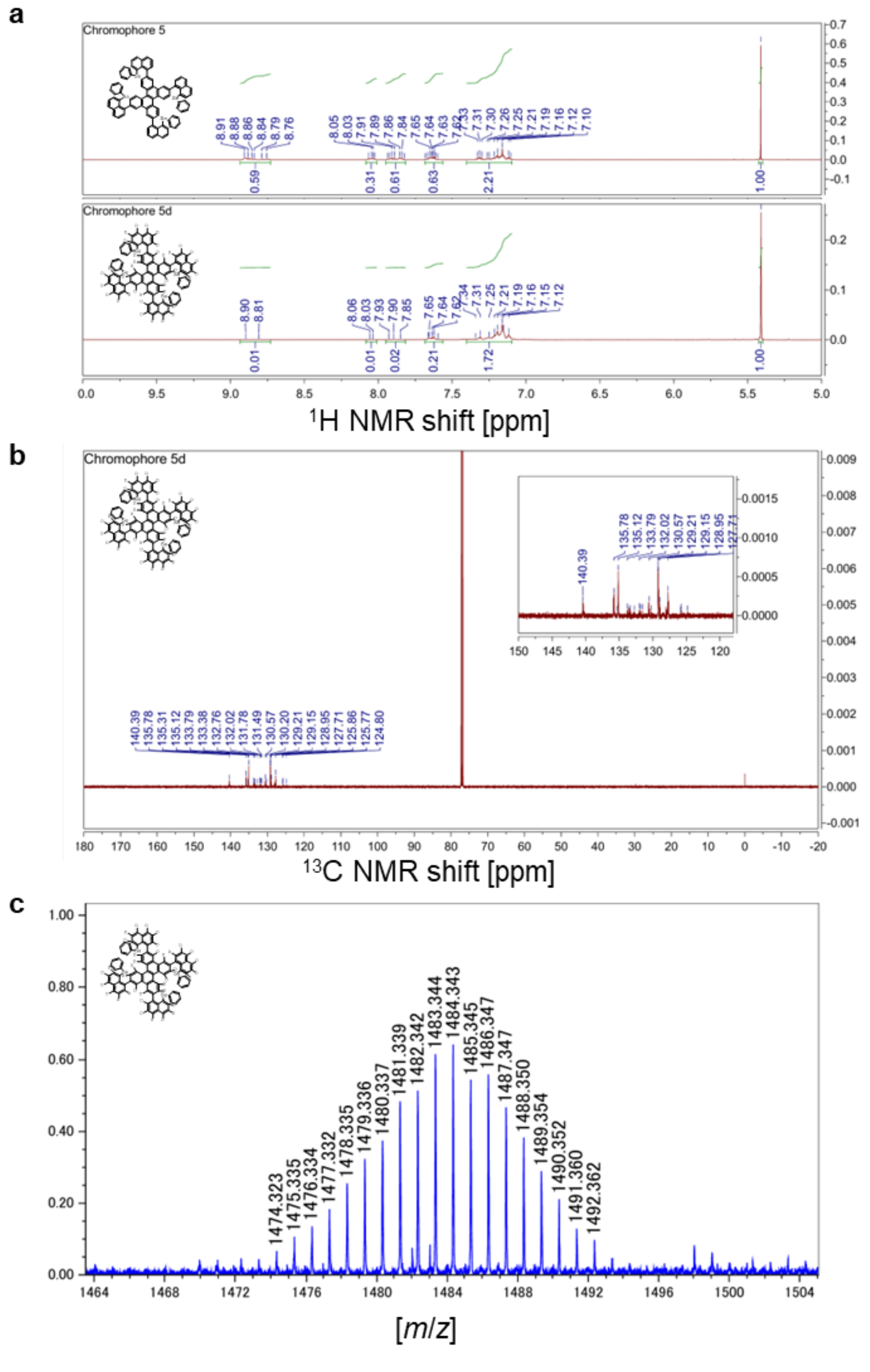

Supplementary Fig. 11. Characterization data of 5d. a, ^{1}H NMR spectra of **5** (top) and **5d** (bottom) in DMSO-d_6 containing dibromomethane. The integral peak intensities were normalized by a signal of dibromomethane. **b**, ^{13}C NMR spectrum in $CDCl_3$. **c**, HRMS-MALDI spectrum.

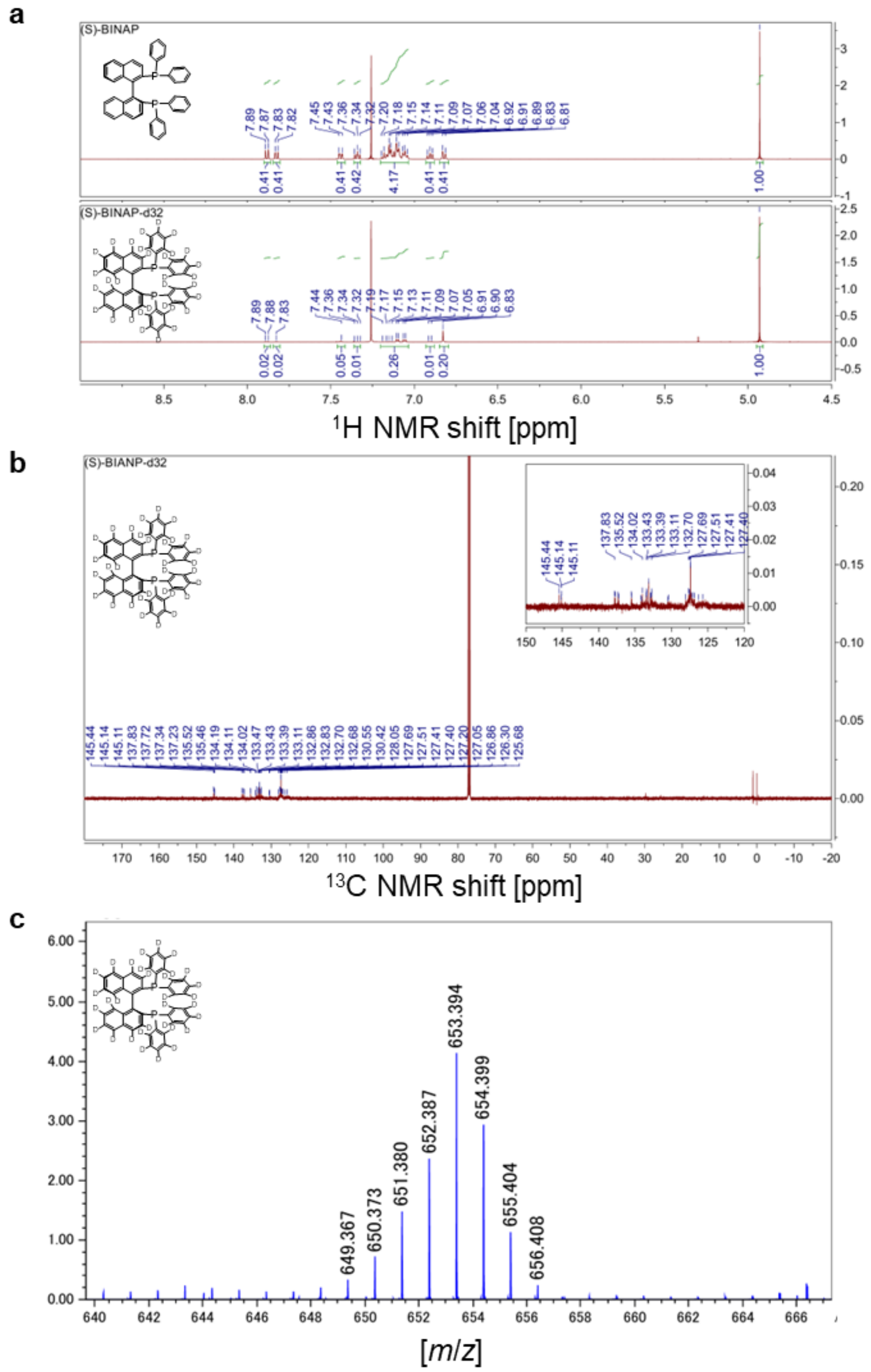

Supplementary Fig. 12. Characterization data of (*S*)-BINAP-d_{32}. a, ^{1}H NMR spectra of (*S*)-BINAP (top) and (*S*)-BINAP-d_{32} (bottom) in $CDCl_3$ containing dibromomethane. The integral peak intensities were normalized by a signal of dibromomethane. **b**, ^{13}C NMR spectrum in $CDCl_3$. **c**, HRMS-MALDI spectrum.

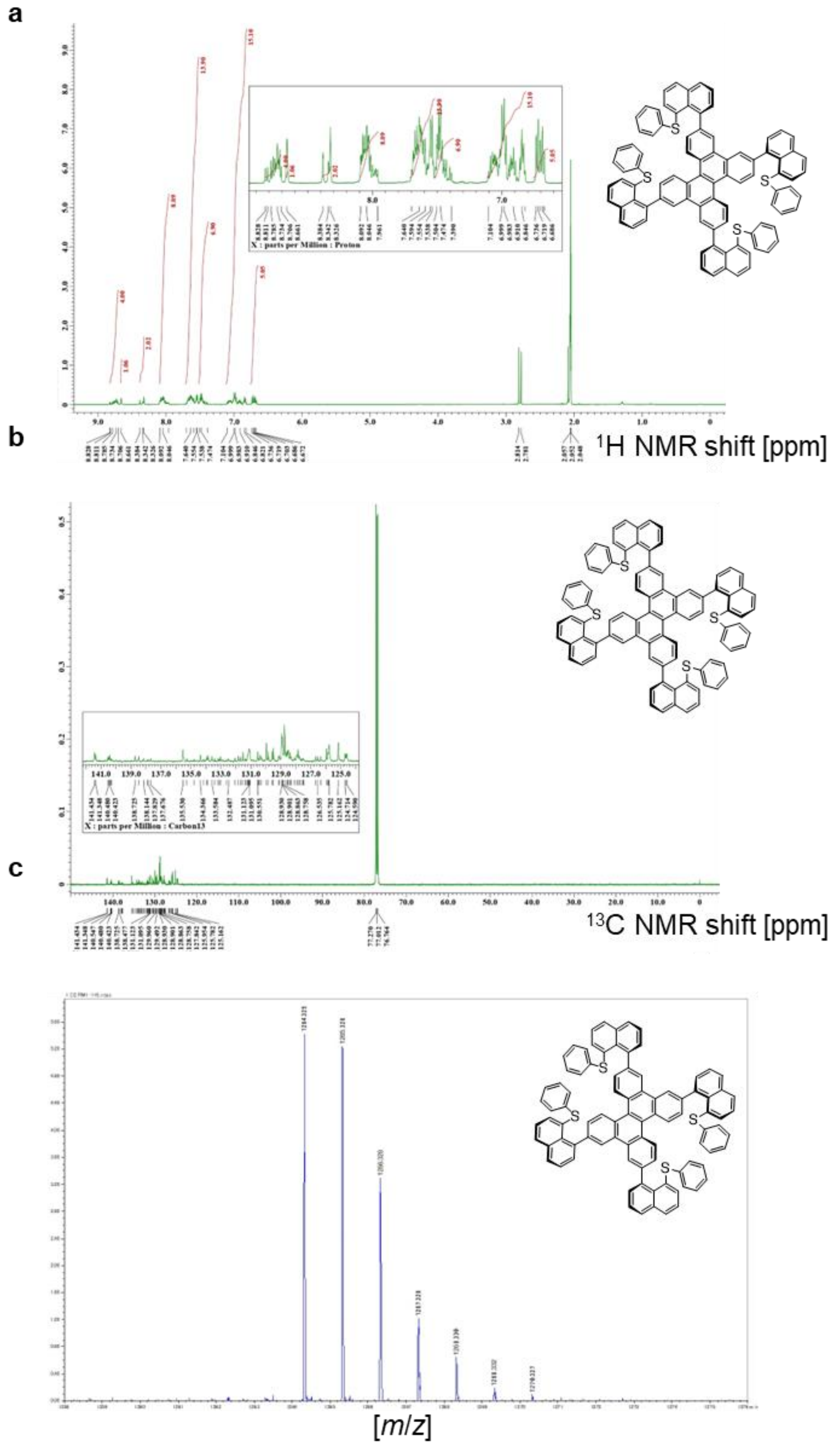

Supplementary Fig. 13. Characterization data of R1. a, ^{1}H NMR spectrum in acetone-d_6. **b**, ^{13}C NMR spectrum in $CDCl_3$. **c**, HRMS-MALDI spectrum.

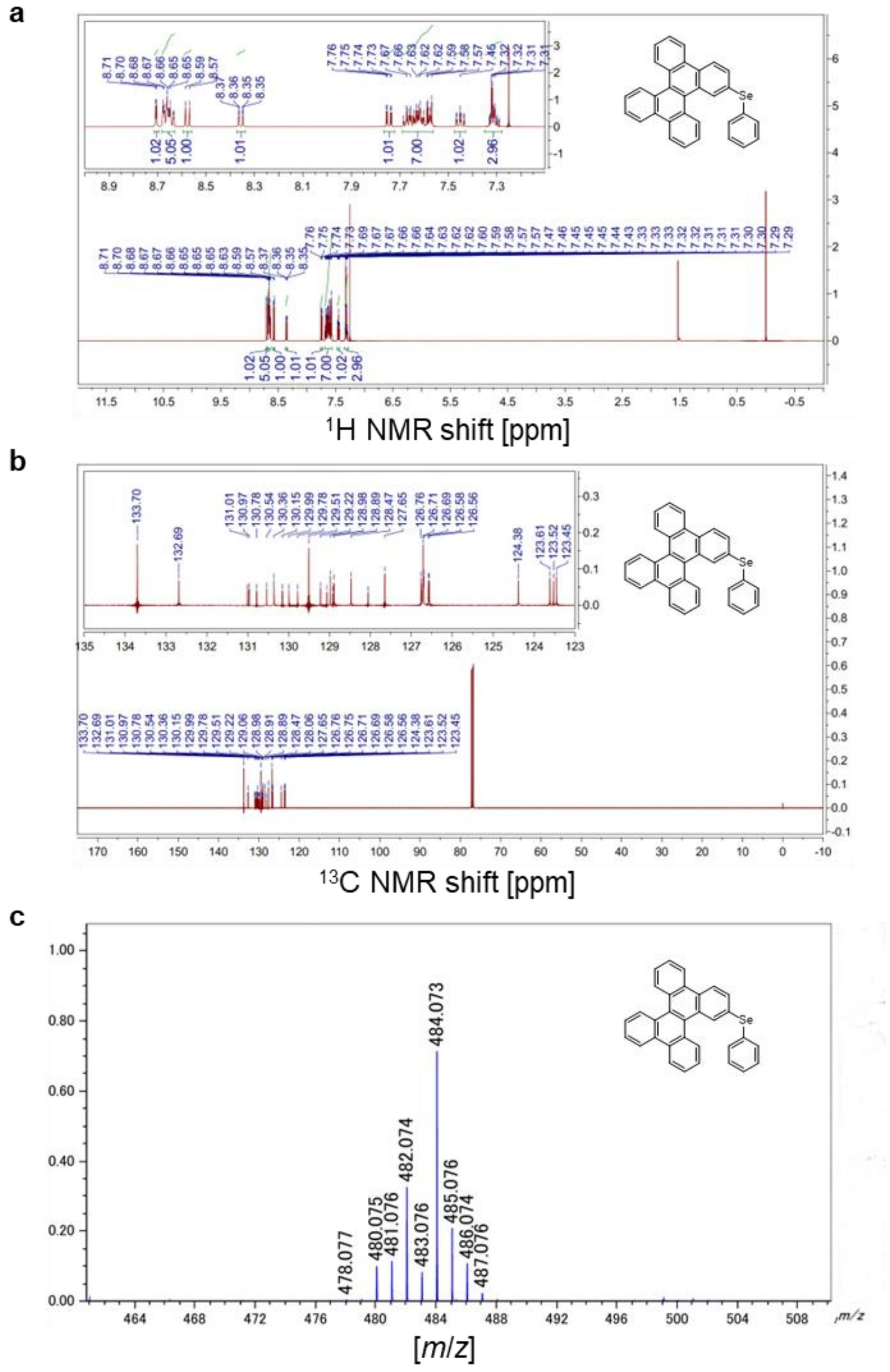

Supplementary Fig. 14. Characterization data of R2. a, ^{1}H NMR spectrum in $CDCl_3$. **b**, ^{13}C NMR spectrum in $CDCl_3$. **c**, HRMS-MALDI spectrum.

Suppl. Note 2. T_1 optimized geometry of chromophores estimated by theoretical calculation (Supplementary Fig. 15)

The optimized structures of the lowest triplet excited state (T_1) of **1**–**5** (Supplementary Fig. 15) were determined by Gaussian09 program based on the density functional theory (DFT). In the calculation, B3LYP and 6-311G+(d,p) were used as a functional and a basis set, respectively. In T_1 optimized structure of **2** and **3**, Se atom is horizontally attached to dibenzo[*g*,*p*]chrysene (DBC) moiety. In **4** and **5**, where naphthalene is introduced as a spacer unit, Se atom is vertically placed on DBC because DBC and naphthalene unit are twisted by the steric hindrance.

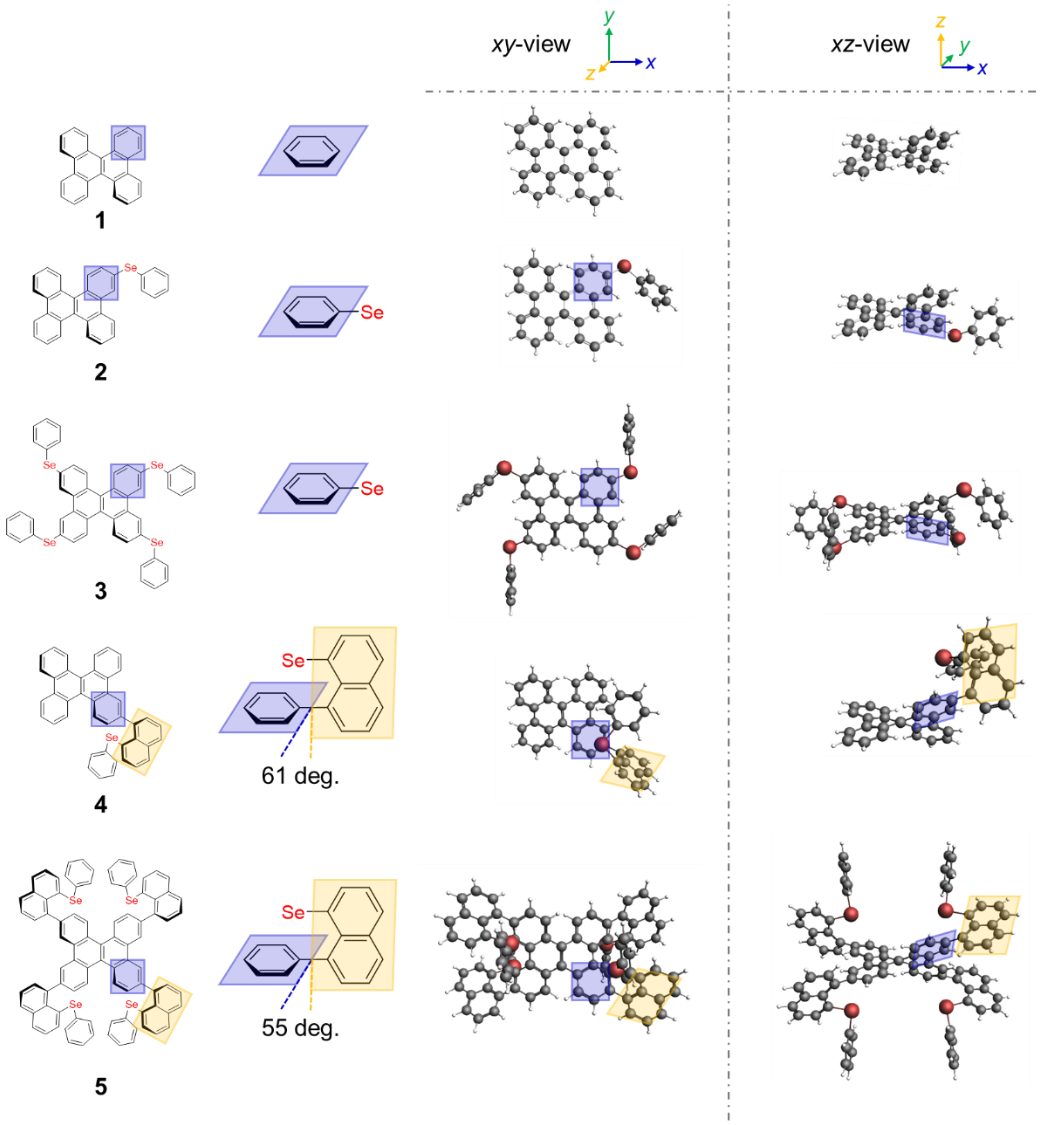

Supplementary Fig. 15. T_1 optimized structures of 1–5.

Suppl. Note 3. Emission behaviour of chromophores in amorphous *β*-estradiol under and soon after UV excitation (Supplementary Fig. 16)

To visually compare the emission behaviour of chromophores **2**–**5**, each film of 0.3 wt% chromophores-doped amorphous *β*-estradiol were prepared (Supplementary Fig. 16a(i)). The photographs of the emission intensities of the films under UV excitation and soon after ceasing ultraviolet (UV) excitation were measured by potable charge-coupled device (CCD) camera. The films showed pink-colour emission under irradiation with UV excitation light (Supplementary Fig. 16a(ii)). At 20–40 ms after ceasing the UV light, similar intensity of afterglow of **2** and **4**, weak afterglow of **3**, and brighter afterglow of **4** were observed (Supplementary Fig. 16a(iii)). Because the absorbance of each film is comparable in the range of UV light spectrum (Supplementary Fig. 16b), the large differences in afterglow intensities are not caused by absorption process but intrinsic emission properties of the excited states. The measurement of emission yield confirmed the relationship **3** < **2** = **4** < **5** for the yield (Φ_p) of room-temperature phosphorescence (RTP). Additionally, the lifetime (τ_p) of RTP of **3** is much shorter (= 6.8 ms) than **2**, **3**, and **5**. When the τ_p is shorter than 10^1 ms, RTP could not well capture after cessation of excitation by using general two-dimensional CCD with frame rate of 50 fps. Due to these relationships of Φ_p and/or τ_p, the different intensities of afterglow emission among **2**–**5** were observed in photographs. The detailed procedure for determining Φ_p is described in Supplementary Note 4-1.

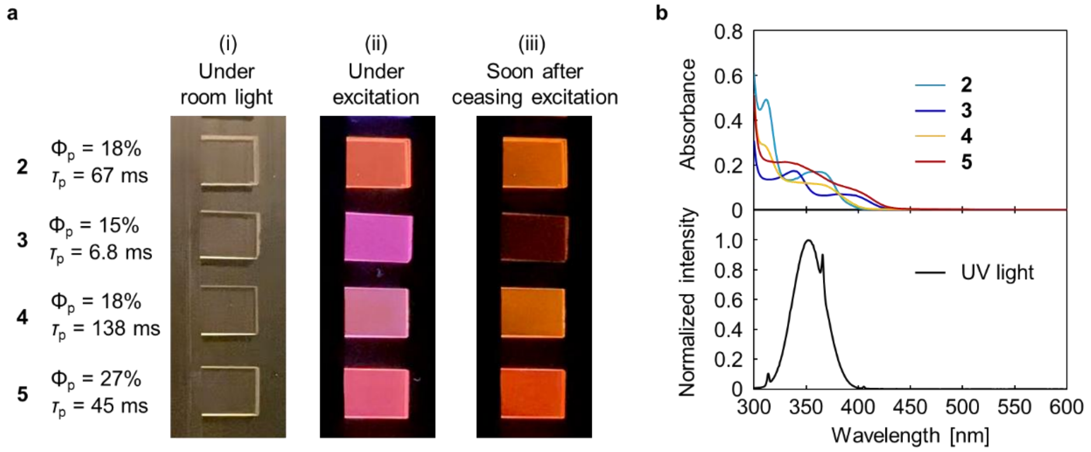

Supplementary Fig. 16. Comparison of the emission behaviour of 2–5 in amorphous *β*-estradiol. **a**, Photographs of 0.3 wt%-chromophore-doped amorphous *β*-estradiol films (i) under room light, (ii) under UV light, and (iii) soon after ceasing UV light. In (iii), the photograph was captured at 20–40 ms after ceasing UV excitation light. **b**, Absorption spectra of films (top) and spectrum of UV light used in the demonstration in Fig. 1b and Supplementary Fig. 16a.

Suppl. Note 4. Photophysical characteristics

Suppl. Note 4-1. Determination procedure for emission yield (Supplementary Figs. 17 and 18)

The emission yield (Φ_e) under excitation at 350 nm was measured by an absolute quantum yield measurement system (C9920-02G, Hamamatsu Photonics, Shizuoka, Japan) (Supplementary Fig. 17). The Φ_e of **2**–**5d** in amorphous *β*-estradiol were measured to be 19.5, 16.9, 18.8, 28.0, 35.9%, respectively. Under irradiation with excitation light, each film of **2**–**5d**-doped amorphous *β*-estradiol showed blue emission as a minor component and red emission as a major component (Supplementary Fig. 18, black line). Because the blue minor emission disappeared and red emission lasted after ceasing excitation light (Supplementary Fig. 18), the blue emission and red emission were attributed to be fluorescence and RTP components, respectively. Since the fluorescence and RTP range are well separated in **2**–**5d**, Φ_p was directly determined using the absolute quantum yield measurement system by setting the wavelength rage to the 540–880 nm for **2**, 550–930 nm for **3**, 520–880 nm for **4**, 550–930 nm for **5**, 550–930 nm for **5d**. The determined value of Φ_p for **2**–**5d** were 18.4, 15.2, 17.6, 27.0, 35.1%, respectively. Using the relationship $\Phi_e = \Phi_f + \Phi_p$, Φ_f of **2**–**5d** in amorphous *β*-estradiol were determined to be 1.1, 1.7, 1.2, 1.0, 0.8%, respectively. Φ_f and Φ_p of 0.3 wt% **1**-doped amorphous *β*-estradiol film were reported to be 20 and 2.9%, respectively[5].

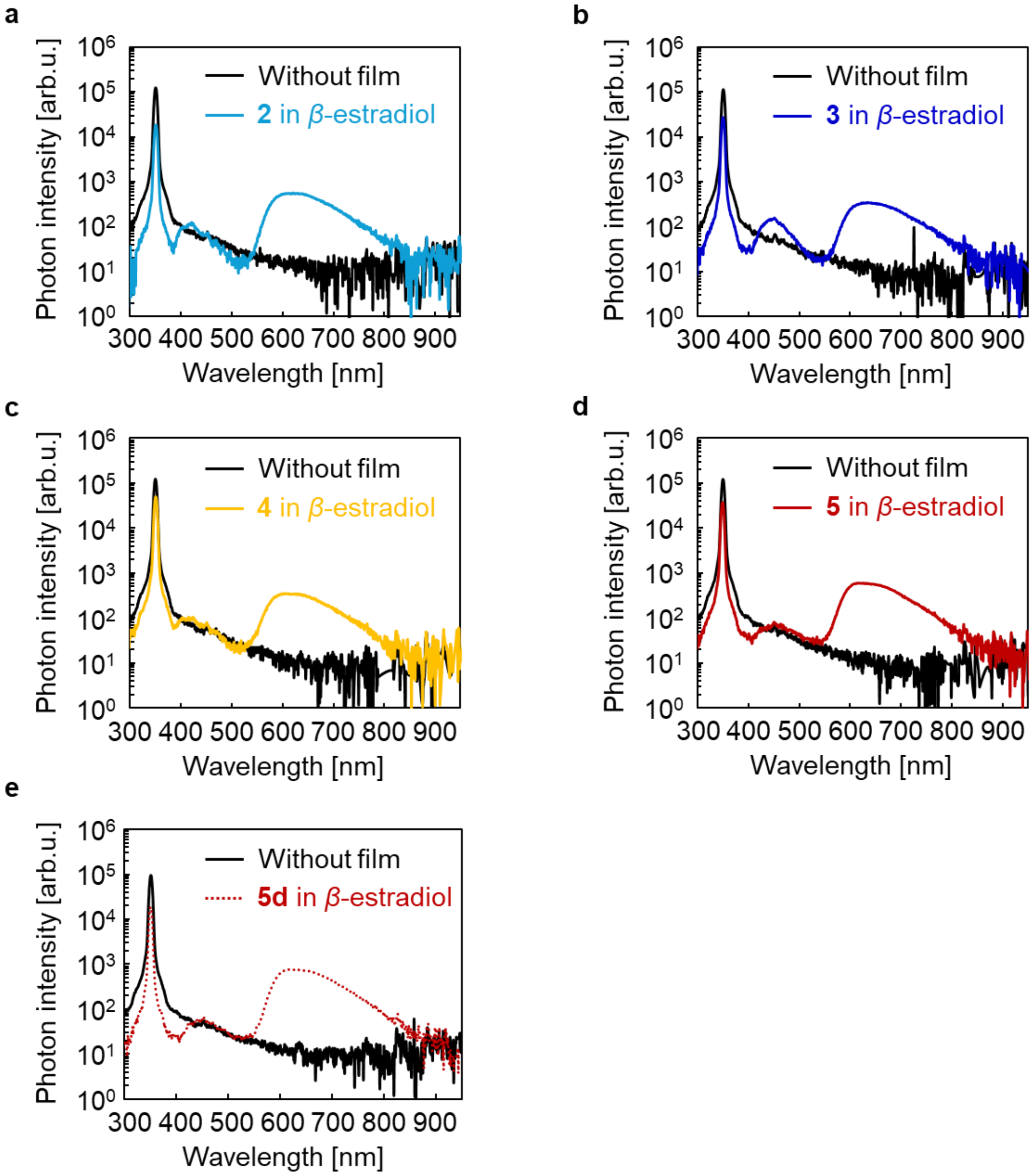

Supplementary Fig. 17. Photon intensity spectra measured in integration sphere equipment with (coloured line) and without (black line) chromophore-doped amorphous *β*-estradiol film. a, **2**-doped amorphous *β*-estradiol film, **b, 3**-doped amorphous *β*-estradiol film, **c**, **4**-doped amorphous *β*-estradiol film, **d**, **5**-doped amorphous *β*-estradiol film, **e**, **5d**-doped amorphous *β*-estradiol film.

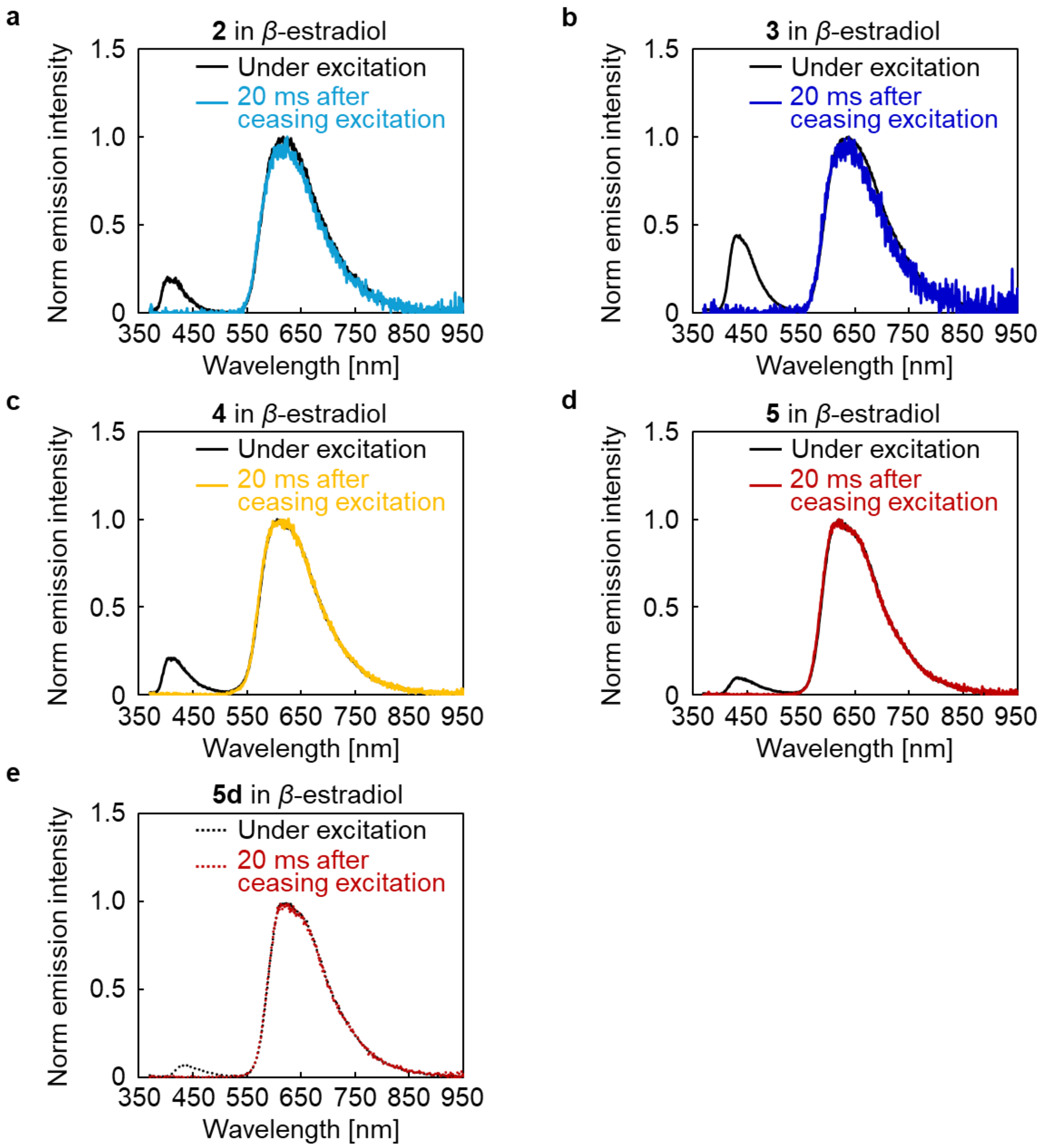

Supplementary Fig. 18. Normalized emission spectra (black line) and normalized afterglow emission spectra at 20 ms after ceasing excitation (coloured line). a, **2**-doped amorphous *β*-estradiol film, **b, 3**-doped amorphous *β*-estradiol film, **c**, **4**-doped amorphous *β*-estradiol film, **d**, **5**-doped amorphous *β*-estradiol film, **e**, **5d**-doped amorphous *β*-estradiol film.

Suppl. Note 4-2. Procedure for making Fig. 2c and reproducibility of Φ_p (Supplementary Fig. 19)

The height of spectral intensity of Fig. 2c was set by taking Φ_p into consideration as follows. The recorded RTP spectra were normalized by the integrated spectral intensity at RTP region to remove the influence of absolute intensity variations such as the distance between sample and photodetector as well as the absorbance of sample (Supplementary Fig. 19a(i)). The normalized spectra were then multiplied by the Φ_p to rescale the height of spectral intensity, providing emission profiles whose integrated areas corresponding to the Φ_p (Supplementary Fig. 19a(ii)). Although the height of spectral intensity in Fig. 2c is normalized by the peak intensity of **5d**, the spectral shape and relative RTP intensity are preserved. To examine the influence of the film inhomogeneity and the doping concentration, the Φ_p of amorphous film of 0.3 wt% **2**-doped *β*-estradiol was remeasured using different sample of 0.3 wt% **2**-doped *β*-estradiol. The value of Φ_p for 0.3 wt% **2**-doped amorphous *β*-estradiol film hardly changed among Lot. 1, 2, and 3 (Supplementary Fig. 19b).

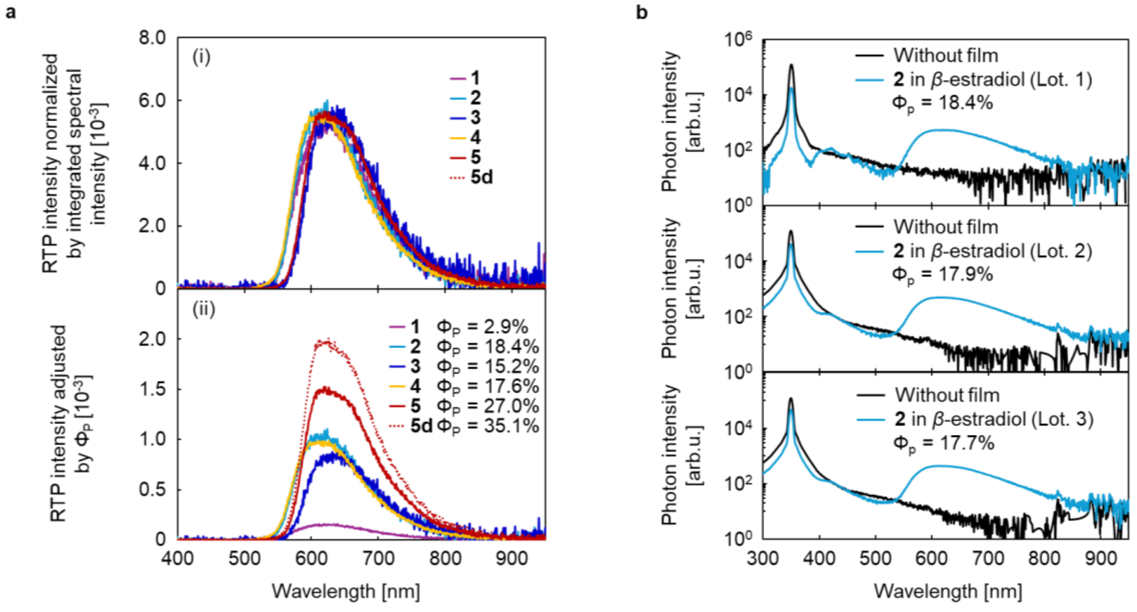

Supplementary Fig. 19. **Procedure for making Fig. 2c and reproducibility of Φ_p. a,** RTP spectra of **1**–**5d** in amorphous *β*-estradiol with a vertical axis of (i) RTP spectral intensity normalized by the integrated spectral intensity and (ii) RTP spectral intensity adjusted by Φ_p. **b**, Photon intensity spectra measured in the integrating sphere equipment with (light blue) and without (black) **2**-doped amorphous *β*-estradiol film.

Suppl. Note 4-3. Transient absorption measurements for determining Φ_{isc} (Supplementary Figs. 20–22 and Supplementary Table 1)

The procedure for measuring intersystem crossing yield from the lowest singlet excited state (S_1) to the triplet excited state of chromophore (Φ_{isc}) that uses the triplet–triplet energy transfer (TTET) from a triplet sensitizer to an acceptor molecule was reported previously in references 38 and 39 in method section of main text. The values of Φ_{isc} for synthesized chromophores were determined via the TTET methods using sample and reference solutions. The sample solution was benzene solution dissolving a synthesized chromophore as the triplet sensitizer and β-carotene (3.0×10^{-3} M) as the acceptor. For the reference solution, benzene solution dissolving benzophenone (BP) as triplet sensitizer and β-carotene (3.0×10^{-3} M) as the acceptor was used. In sample and reference solution, the absorbance at 355 nm, which was caused by the triplet sensitizer, was adjusted to be 1.0 in a 1 mm thick quartz cell. The solutions were degassed three times in freezing condition and sealed immediately before measuring the transient absorption. The transient absorption of the sample and reference solution were measured using a sub-nanosecond transient absorption spectrophotometer (picoTAS, Unisoku, Osaka, Japan) with a 355 nm Q-switched microchip laser (PNV-M02510-1×0, Teem Photonics, Meylan, France).

The time changes of transient absorption at 530 nm were initially rising and gradually decreased in both sample (Supplementary Fig. 20, orange) and reference (Supplementary Fig. 20, black) solution. This initial rise originated from TTET from the triplet sensitizer to β-carotene because time-resolved transient absorption spectra was changed from transient absorption caused by the triplet sensitizer to that caused by β-carotene with a peak around 520–530 nm over the time (Supplementary Fig. 21). The transient absorption decay characteristics were fitted using the following equation,

$$\Delta\mathrm{Abs}(t) = A[1 - \exp(-t/\tau_1)]\exp(-t/\tau_2) + B\exp(-t/\tau_1), \text{ [Supplementary Equation (1)]}$$

where t is the time after irradiation with an excitation pulse laser at 355 nm. The determined values of A, τ_1, τ_2, and B were summarized in Supplementary Table 1.

Next, the triplet lifetime (τ_0) of synthesized chromophores and BP in benzene were measured using the sample solution in the absence of β-carotene and the reference solution in the absence of β-carotene, respectively (Supplementary Fig. 22). By substituting the τ_0 and τ_1 into $\Phi_{TTET} = (\tau_0 - \tau_1)/\tau_0$, the efficiency of TTET from the triplet sensitizer to β-carotene (Φ_{TTET}) was calculated as shown in Supplementary Table 1. Because optics and excitation power are the same in the transient absorption measurement

of sample and reference solution, the following equation can be obtained.

$A^S/A^R = \Phi_{isc}^S\Phi_{TTET}^S/\Phi_{isc}^R\Phi_{TTET}^R$, [Supplementary Equation (2)]

where, A^S and A^R are the A of sample and reference solutions, respectively, Φ_{isc}^S and Φ_{isc}^R are the Φ_{isc} of triplet sensitizers of sample and reference solutions, respectively, and Φ_{TTET}^S and Φ_{TTET}^R are the Φ_{TTET} of sample and reference solutions, respectively. Since Φ_{isc} of BP acting as the triplet sensitizer of reference solution tends to be 1, the Φ_{isc} of synthesized chromophores were obtained by using the Supplementary Equation (2). The accuracy of Φ_{isc} determined in this method has been reported to be limited to within ±25%. Thus, the Φ_{isc} of **2**–**5d** were determined to be 112 ± 28, 113 ± 28, 112 ± 28, 122 ± 30, 126 ± 31% (Supplementary Table 1). The value of Φ_{isc} of **1** was reported as 87 ± 22% using the same procedure[5].

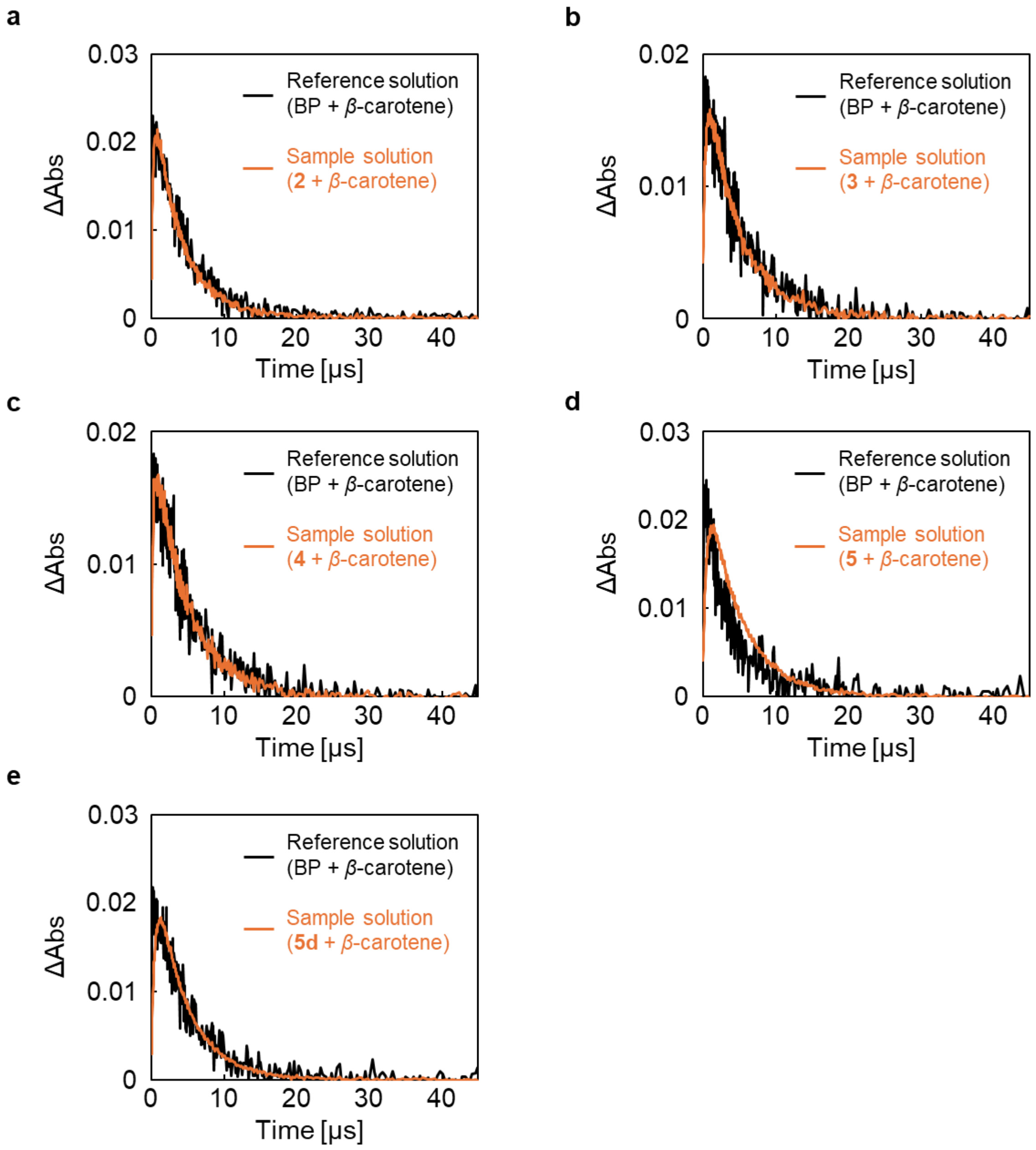

Supplementary Fig. 20. Decay characteristics of transient absorption at 530 nm of sample and reference solutions. The sample solution was benzene solution containing a synthesized chromophore as a triplet sensitizer and *β*-carotene (3.0×10^{-3} M) as an acceptor. The sample solution was benzene solution containing BP as a triplet sensitizer and *β*-carotene (3.0×10^{-3} M) as an acceptor. **a**, Triplet sensitizer of sample solution is **2**. **b**, Triplet sensitizer of sample solution is **3**. **c**, Triplet sensitizer of sample solution is **4**. **d**, Triplet sensitizer of sample solution is **5**. **e**, Triplet sensitizer of sample solution is **5d**.

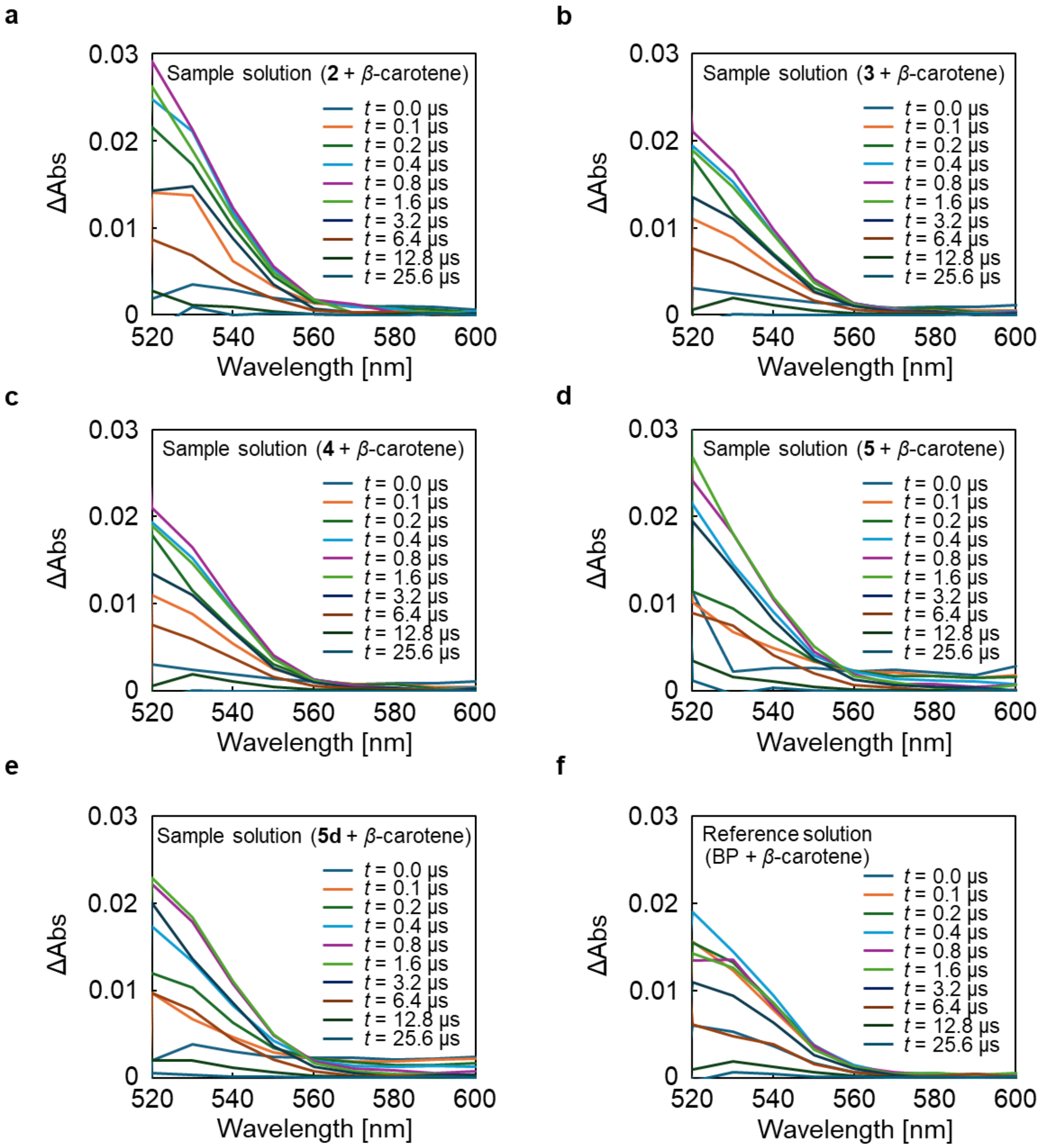

Supplementary Fig. 21. Time-resolved transient absorption spectra of sample and reference solutions. The sample solution was benzene solution containing a synthesized chromophore as a triplet sensitizer and *β*-carotene (3.0×10^{-3} M) as an acceptor. The sample solution was benzene solution containing BP as a triplet sensitizer and *β*-carotene (3.0×10^{-3} M) as an acceptor. **a**, Triplet sensitizer is **2**. **b**, Triplet sensitizer is **3**. **c**, Triplet sensitizer is **4**. **d**, Triplet sensitizer is **5**. **e**, Triplet sensitizer is **5d**. **f**, Triplet sensitizer is BP.

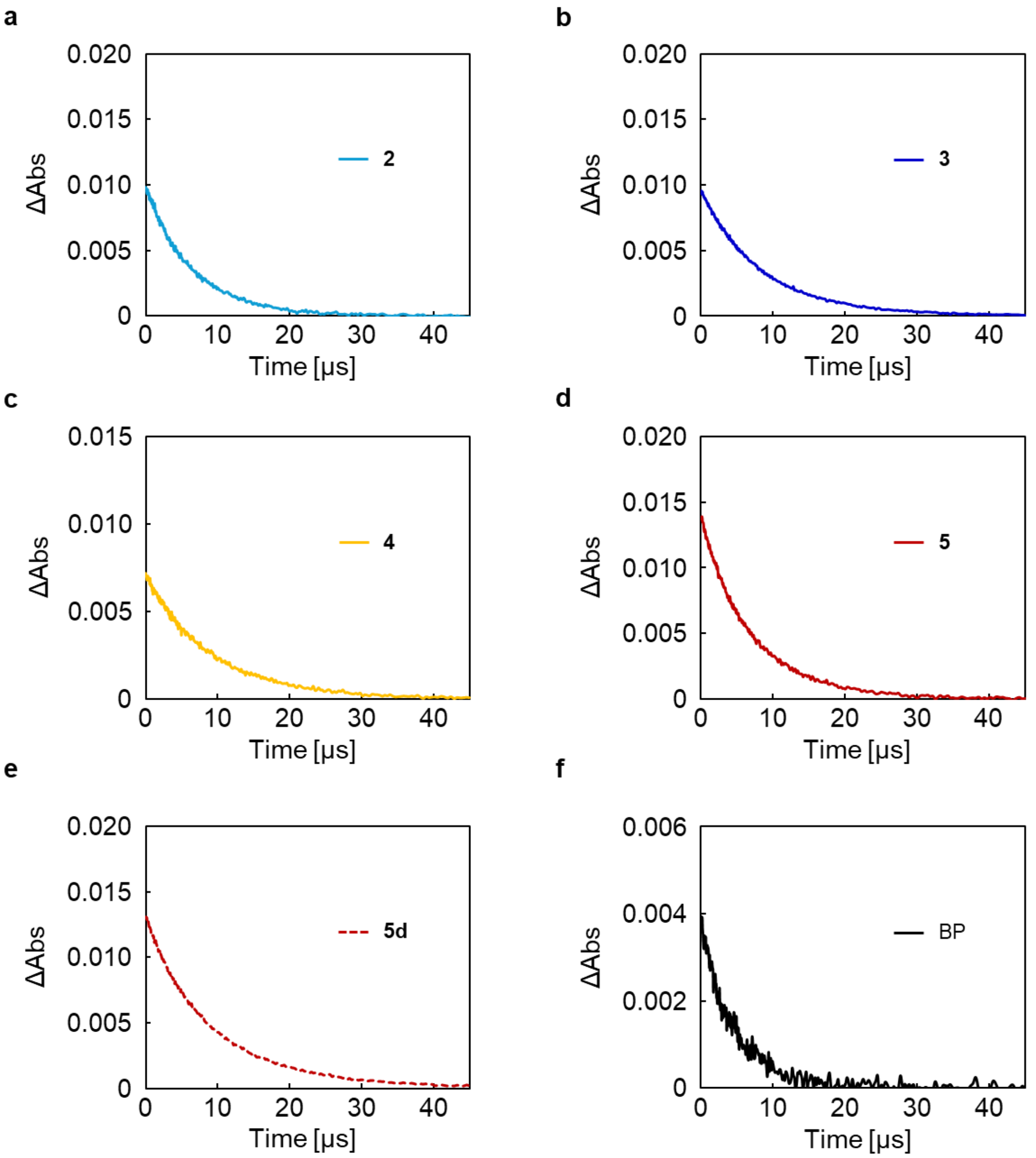

Supplementary Fig. 22. Transient absorption decay characteristics of synthesized chromophores and BP in deoxygenated benzene. a, Chromophore **2**. **b**, Chromophore **3**. **c**, Chromophore **4**. **d**, Chromophore **5**. **e**, Chromophore **5d**. **f**, BP. The optics and excitation power are the same as in Supplementary Figs. 20 and 21.

Supplementary Table 1. Parameters used to determine Φ_{isc} of **2–5d** in benzene.

Role of solution	Triplet sensitizer	Concentration of *β*-carotene [M]	*A*	*B*	τ_1 [μs]	τ_0 [μs]	Φ_{TTET}	Φ_{isc}
Reference	BP	3.0×10^{-3}	0.0236	0.00995	0.112	2.81	0.960	
Sample	**2**	3.0×10^{-3}	0.0264	0.00479	0.261	6.40	0.959	1.12 ± 0.28
Reference	BP	3.0×10^{-3}	0.0183	0.00764	0.122	5.59	0.978	
Sample	**3**	3.0×10^{-3}	0.0202	0.00430	0.399	8.34	0.952	1.13 ± 0.28
Reference	BP	3.0×10^{-3}	0.0183	0.00764	0.122	5.59	0.978	
Sample	**4**	3.0×10^{-3}	0.0203	0.00403	0.278	8.99	0.969	1.12 ± 0.28
Reference	BP	3.0×10^{-3}	0.0236	0.00752	0.0659	5.13	0.987	
Sample	**5**	3.0×10^{-3}	0.0278	0.00343	0.596	13.7	0.956	1.22 ± 0.30
Reference	BP	3.0×10^{-3}	0.0217	0.00716	0.0870	3.80	0.977	
Sample	**5d**	3.0×10^{-3}	0.0269	0.00355	0.596	18.2	0.967	1.26 ± 0.31

Suppl. Note 4-4. Summary of photophysical characteristics relating to RTP (Supplementary Table 2)

Φ_p and τ_p are generally expressed as following equations[6],

$\Phi_p = \Phi_{isc} k_r^T \tau_p$, [Supplementary Equation (3)]

$\tau_p = 1/(k_r^T + k_{nr}^T)$, [Supplementary Equation (4)]

where k_r^T is the rate constant of radiative transition from T_1 and k_{nr}^T is the rate constant of nonradiative transition from T_1. Because the Φ_{isc} determined by the transient absorption measurement described in the previous section have errors of ±25%, values of Φ_{isc} estimated from 1 – Φ_f were used for determining the k_r^T. The photophysical parameters relating to RTP for **1**–**5d** in amorphous *β*-estradiol were summarized in Supplementary Table 2.

Supplementary Table 2. Summary of photophysical parameters relating RTP.

Chromophores[a)]	Φ_f [%]	Φ_{isc} [%]	Φ_p [%]	τ_p [ms]	k_r^T [s^{-1}]	k_{nr}^T [s^{-1}]
**1**[b)]	20	80[c)] (87 ± 22)[d)]	2.9	580	0.063	1.7
**2**	1.1	99[c)] (112 ± 28)[d)]	18.4	66.5	2.8	12
**3**	1.7	98[c)] (113 ± 28)[d)]	15.2	6.84	23	1.2×10^2
**4**	1.2	99[c)] (112 ± 28)[d)]	17.6	138	1.3	5.9
**5**	1.0	99[c)] (122 ± 30)[d)]	27.0	44.8	6.1	16
**5d**	0.8	99[c)] (126 ± 31)[d)]	35.1	56.5	6.3	11

[a)]The concentration of chromophores was 0.3 wt% in amorphous *β*-estradiol. [b)]Previously reported in Supplementary reference 5. [c)]Values determined from 1 – Φ_f. [d)]Values measured by transient absorption techniques.

Suppl. Note 4-5. Optical properties in solution (Supplementary Fig. 23)

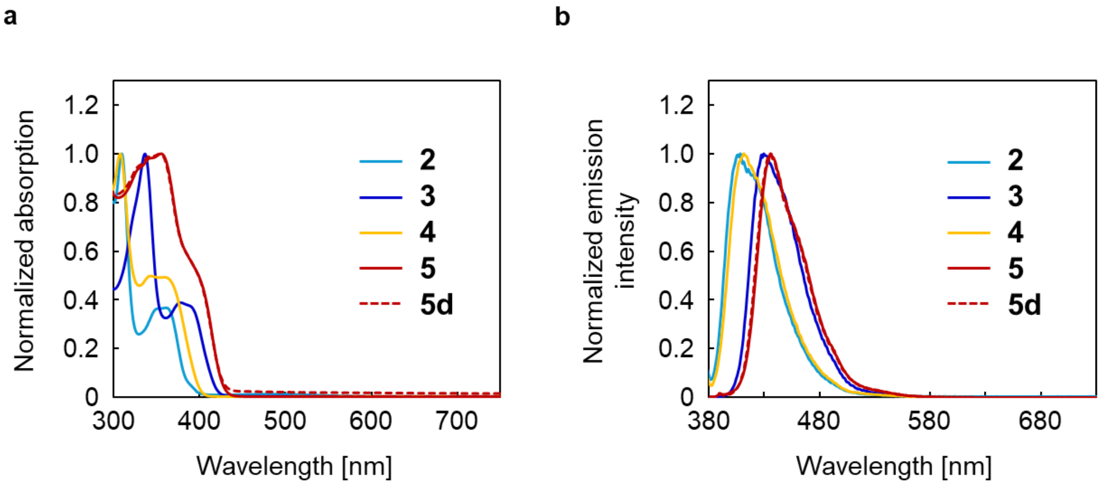

Supplementary Fig. 23. Spectral properties of 2–5d in toluene. a, Normalized absorption spectra, **b**, Normalized fluorescence spectra. The concentration of chromophores is 1.0×10^{-5} M in toluene.

Suppl. Note 5. Theoretical calculations

Suppl. Note 5-1. Calculated values of k_r^T and $<T_1|\mathbf{H}_{SO}|S_0>^2$ (Supplementary Fig. 24)

Calculation procedure of T_1 optimized structure for **1**–**5** was described in Suppl. Note 2. Using the T_1 optimized structure (Supplementary Fig. 15), single-point calculations were performed using the time-dependent (TD) DFT with the Amsterdam Density Functional (ADF) 2025 package to calculate k_r^T and the spin–orbit coupling (SOC) between T_1 and S_0 ($<T_1|\mathbf{H}_{SO}|S_0>$). The parameter $<S_n|\mathbf{H}_{SO}|T_1>$ and $<T_m|\mathbf{H}_{SO}|S_0>$ were treated as a perturbation based on scalar relativistic orbitals using the PBE0 as a functional and TZP as a basis sets. The scalar relativistic-time-dependent DFT calculations included 10 singlet and 10 triplet excitations, which were used as the basis for the perturbative expansions in the calculations.

From the above calculations, the good correlation between calculated $<T_1|\mathbf{H}_{SO}|S_0>^2$ and measured k_{nr}^T (Supplementary Fig. 24a) and between calculated k_r^T and measured k_r^T (Supplementary Fig. 24b) were observed among synthesized chromophores. From **2** to **3**, the magnitude of calculated k_r^T enhancement (Supplementary Fig. 24b, blue arrow) was smaller than that of $<T_1|\mathbf{H}_{SO}|S_0>^2$ enhancement (Supplementary Fig. 24a, blue arrow). In contrast, from **4** to **5**, calculated k_r^T was more enhanced compared with $<T_1|\mathbf{H}_{SO}|S_0>^2$ (Supplementary Figs. 24a and 24b, red arrows). Thus, the more selective enhancement of k_r^T relative to k_{nr}^T of **5** was observed from theoretical calculations as well as optical measurements. Therefore, the discussion on the differences in measured k_r^T and k_{nr}^T based on the quantum chemical calculation is reasonable.

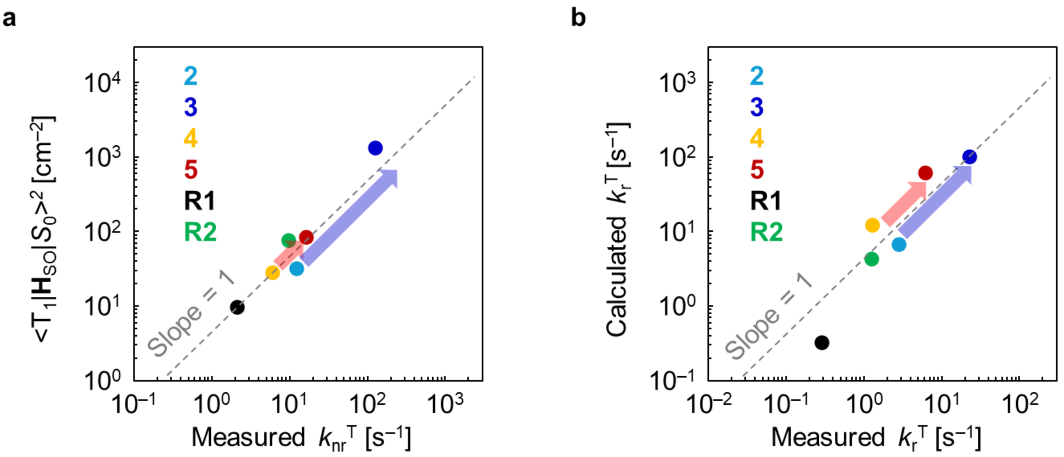

Supplementary Fig. 24. Relationship between measured and calculated parameters relating phosphorescence process. a, Correlation between measured k_{nr}^T and calculated $<T_1|\mathbf{H}_{SO}|S_0>^2$. **b,** Correlation between measured and calculated k_r^T.

Suppl. Note 5-2. Relationship between orbital orientation characteristics and spin flip (Supplementary Fig. 25)

Transitions between singlet and triplet states necessarily involve a change in the spin angular momentum ($\vec{\mathbf{s}}$). According to the conservation of total angular momentum, such a spin conversion must be accompanied by the change ($\Delta\mathbf{L}$) in the orbital angular momentum ($\vec{\mathbf{l}}$). The resulting $\Delta\mathbf{L}$ exserts a torque on the electron, thereby promoting spin flipping. The magnitude of $\Delta\mathbf{L}$ depends on the vector cross product of the respective $\vec{\mathbf{l}}$[7]. Therefore, understanding the direction of the $\vec{\mathbf{l}}$ is essential when discussing the electronic transitions that require spin flipping.

The orbital orientation axis is directly related to orientation of $\vec{\mathbf{l}}$. In case of p_z orbital, $\vec{\mathbf{l}}$ exhibits an effective component within the xy-plane (Supplementary Fig. 25a(i)). When the projection component is considered, $\vec{\mathbf{l}}$ is oriented perpendicular to the orbital orientation axis (Supplementary Fig. 25a(ii)). When two orbitals are oriented along the same axis, their corresponding $\vec{\mathbf{l}}$ are aligned in the same direction (Supplementary Fig. 25b(i)). In this case, $\Delta\mathbf{L}$ becomes negligible, and no torque is generated to induce spin flip (Supplementary Fig. 25b(ii)). Conversely, $\Delta\mathbf{L}$ increases when the two orbitals are oriented in different directions and reaches a maximum when their orbital orientation axes are perpendicular (Supplementary Fig. 25b(iii)). Under these conditions, the electron experiences a large torque, which strongly promotes the spin-flip process (Supplementary Fig. 25b(iv)). Therefore, discussing changes in orbital orientation axis is effectively equivalent to discussing the orientation and magnitude of $\Delta\mathbf{L}$, providing an intuitive approach for visualizing the magnitude of SOC.

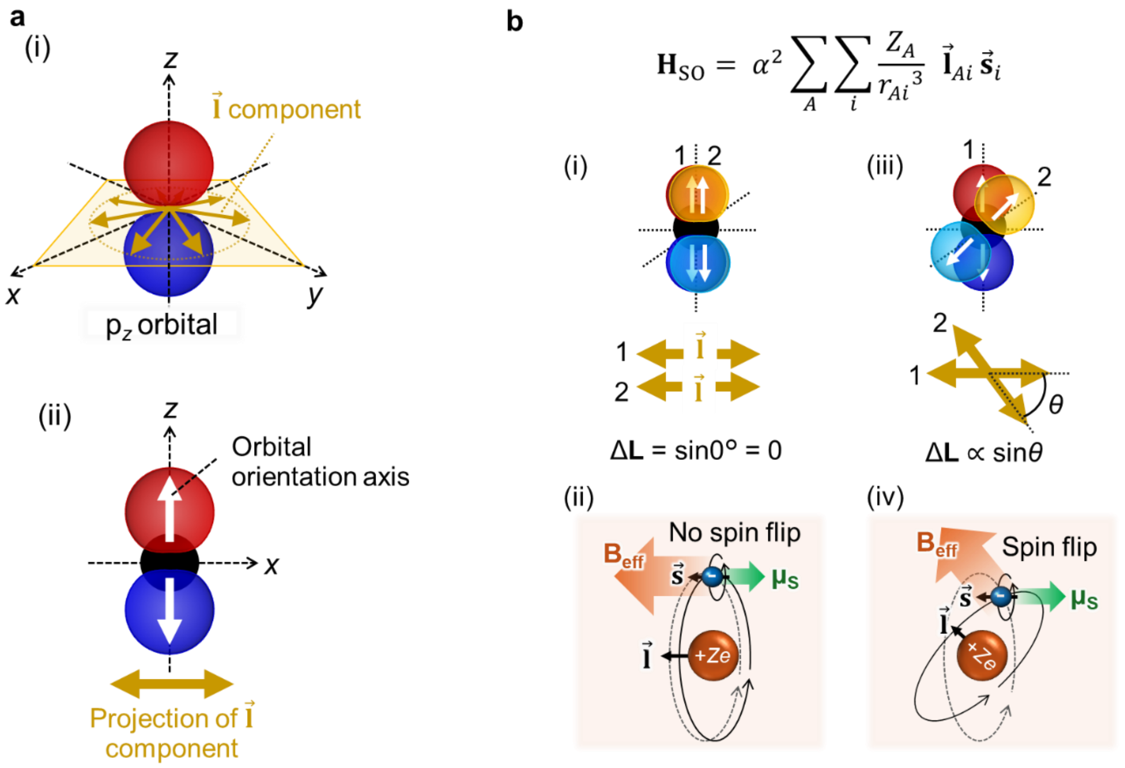

Supplementary Fig. 25. Relationship between orbital orientation characteristics and spin flip. a, Relationship between (i) atomic orbital and effective component of $\vec{l}$ and (ii) orbital orientation axis and projected component of $\vec{l}$. **b,** Schematic illustrations of (i) two orbitals having the same orientation axis, (ii) the absence of torque generation for spin flipping, (iii) two orbitals oriented along different axes, and (iv) the electron experiencing a large torque that promotes spin flipping. In panels (ii) and (iv) of **b**, $\mathbf{B}_{eff}$ and $\boldsymbol{\mu}_S$ represent the effective magnetic field generated by $\vec{l}$ and the electron spin magnetic momentum, respectively.

Suppl. Note 5-3. Molecular orbitals relating to <T_1|H_{SO}|S_0>^{2} (Supplementary Fig. 26)

The T_1–S_0 transition of **3** and **5** are mainly constructed from HOMO and LUMO (Supplementary Fig. 26a, top and 26b, top). Because **3** and **5** have similar nature in both the HOMO and LUMO at DBC unit, the orbital's nature around the Se atom were focused to explain the large difference in <T_1|**H_{SO}**|S_0>^{2} between **3** and **5**. For **3**, HOMO and LUMO delocalize to Se atom (Supplementary Fig. 26a, bottom). Similarly, HOMO of **5** is delocalized on Se atom. However, in LUMO of **5**, a significantly small electron density at Se atom was observed and could not be confirmed using isovalue of more than 0.008 (Supplementary Fig. 26b, bottom).

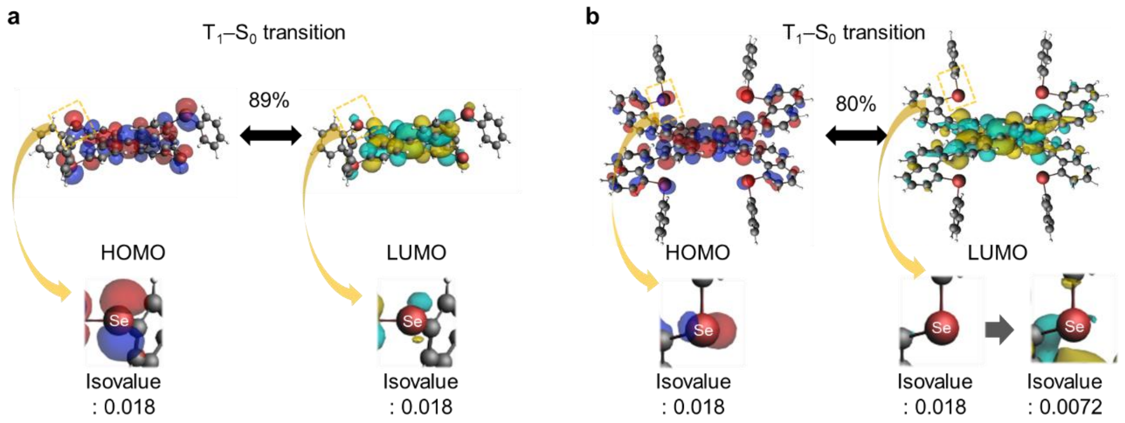

Supplementary Fig. 26. Molecular orbitals relating to <T_1|H_{SO}|S_0>^{2} for 3 and 5. a, HOMO and LUMO of **3**. **b**, HOMO and LUMO of **5**.

Suppl. Note 5-4. Calculated parameters relating to k_r^T (Supplementary Fig. 27)

The calculated values of $\langle S_n|\mathbf{H}_{SO}|T_1\rangle^2 E_{Sn\text{–}T1}^{-2}\mu_{Sn\text{-}S0}^2$ are the largest at $n = 6$, $n = 5$, $n = 7$, and $n = 2$ for **2**, **3**, **4**, and **5**, respectively (Supplementary Fig. 27a). Accordingly, these n mostly contribute to the first term of Equation (4) for chromophores **2**–**5**, respectively. For the second term of Equation (4), the largest value of $\langle T_m|\mathbf{H}_{SO}|S_0\rangle^2 E_{Tm\text{–}S0}^{-2}\mu_{Tm\text{-}T1}^2$ indicate that $m = 9$, $m = 7$, $m = 5$, and $m = 9$ dominantly contribute to k_r^T for **2**, **3**, **4**, and **5**, respectively (Supplementary Fig. 27b).

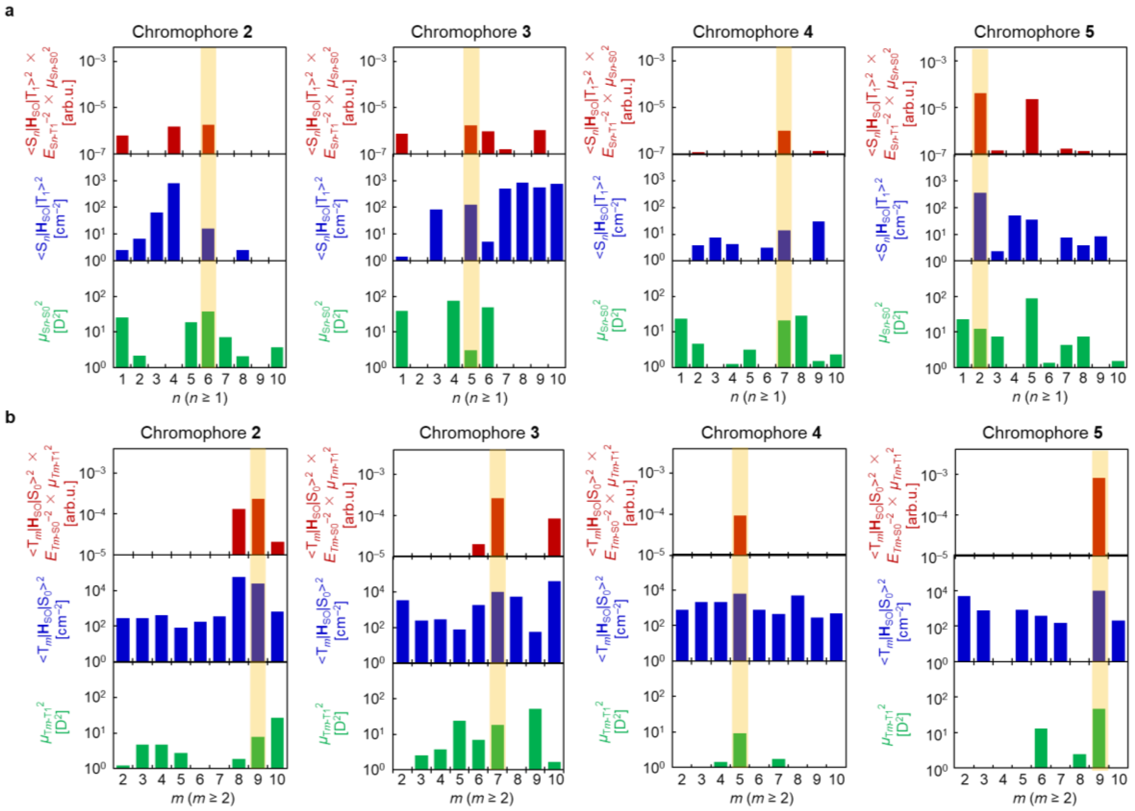

Supplementary Fig. 27. Histograms of calculated parameters relating to k_r^T. a, Histograms of $\langle S_n|\mathbf{H}_{SO}|T_1\rangle^2 E_{Sn\text{–}T1}^{-2}\mu_{Sn\text{-}S0}^2$ (top), $\langle S_n|\mathbf{H}_{SO}|T_1\rangle^2$ (middle), and $\mu_{Sn\text{-}S0}^2$ (bottom) for **2**–**5**. **b,** Histograms of $\langle T_m|\mathbf{H}_{SO}|S_0\rangle^2 E_{Tm\text{–}S0}^{-2}\mu_{Tm\text{-}T1}^2$ (top), $\langle T_m|\mathbf{H}_{SO}|S_0\rangle^2$ (middle), and $\mu_{Tm\text{-}T1}^2$ (bottom) for **2**–**5**.

Suppl. Note 5-5. Contribution of $E_{\mathrm{S}_n\text{-}\mathrm{S}_0}$ and $E_{\mathrm{T}_m\text{-}\mathrm{S}_0}$ to k_r^T (Supplementary Fig. 28)

From Equation (4), first term involves the $E_{\mathrm{S}_n-\mathrm{T}_1}$ contribution and second term involves $E_{\mathrm{T}_m-\mathrm{S}_0}$ (Supplementary Fig. 28a). Therefore, the energy term also relates to k_r^T. In the contribution in first term of Equation (4), the product of $\frac{\langle \mathrm{S}_n|\mathbf{H}_\mathrm{SO}|\mathrm{T}_1\rangle^2}{E_{\mathrm{S}_n-\mathrm{T}_1}{}^2}\mu_{\mathrm{S}_n-\mathrm{S}_0}{}^2$ is the largest at n = 2 for **5** (Supplementary Fig. 28b(i)). However, compared with the differences in the magnitude of $<\mathrm{S}_n|\mathbf{H}_\mathrm{SO}|\mathrm{T}_1>^2$ (Supplementary Fig. 28b(ii)) and $\mu_{\mathrm{S}_n-\mathrm{S}_0}{}^2$ among n = 1 to n = 10 (Supplementary Fig. 28b(iii)), the $E_{\mathrm{S}_n-\mathrm{T}_1}{}^2$ does not drastically change among n = 1 to n = 10 (Supplementary Fig. 28b(iv)). This indicates that contribution of the $E_{\mathrm{S}_n-\mathrm{T}_1}{}^2$ to the k_r^T is relatively minor. Such a trend was observed in the second term of Equation (4) (Supplementary Fig. 28c(i)–(iv)). Therefore, the energy term is not included in the definition of cooperative effect.

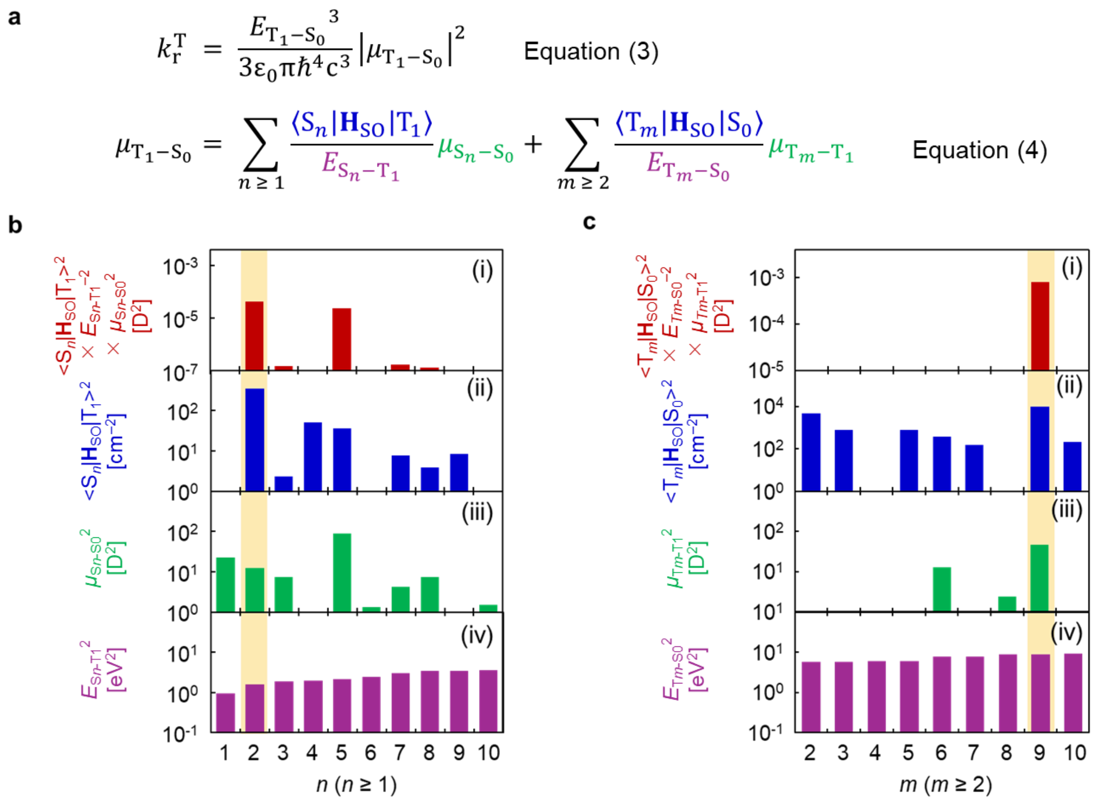

Supplementary Fig. 28. Parameters affecting k_r^T. a, Equations of k_r^T. **b,** Histograms of (i) $\frac{\langle \mathrm{S}_n|\mathbf{H}_\mathrm{SO}|\mathrm{T}_1\rangle^2}{E_{\mathrm{S}_n-\mathrm{T}_1}{}^2}\mu_{\mathrm{S}_n-\mathrm{S}_0}{}^2$, (ii) $\langle \mathrm{S}_n|\mathbf{H}_\mathrm{SO}|\mathrm{T}_1\rangle^2$, (iii) $\mu_{\mathrm{S}_n-\mathrm{S}_0}{}^2$, and (iv) $E_{\mathrm{S}_n-\mathrm{T}_1}{}^2$ for each n. **c,** Histograms of (i) $\frac{\langle \mathrm{T}_m|\mathbf{H}_\mathrm{SO}|\mathrm{S}_0\rangle^2}{E_{\mathrm{T}_m-\mathrm{S}_0}{}^2}\mu_{\mathrm{T}_m-\mathrm{T}_1}{}^2$, (ii) $\langle \mathrm{T}_m|\mathbf{H}_\mathrm{SO}|\mathrm{S}_0\rangle^2$, (iii) $\mu_{\mathrm{T}_m-\mathrm{T}_1}{}^2$, and (iv) $E_{\mathrm{T}_m-\mathrm{S}_0}{}^2$ for each m.

Suppl. Note 5-6. Calculation results and photophysical properties of chromophore R2 (Supplementary Figs. 29–31 and Supplementary Table 3)

To clearly clarify the contribution of the intramolecular external heavy atom effect (HAE) derived from spatial proximity between heavy atom and central π-core, chromophore **R2** in which a phenylseleno substituent is horizontally introduced at the 3-position of the DBC core was synthesized (Supplementary Fig. 29a). In **R2**, Se atom is not directly conjugated with emissive core because of no electron density at 3-position of DBC. Therefore, **R2** has a potential characteristic of the intramolecular external HAE. Additionally, the distance between Se and the 3-position of DBC is 1.94 Å in T_1 optimized geometry (Supplementary Fig. 29b). Because the distance between Se and C of DBC for **4** (3.19 Å) and **5** (3.05 Å) are more separated spatially, the intramolecular external HAE may play significantly in **R2**.

The **R2** was doped in the amorphous *β*-estradiol at a concentration with 0.3 wt%. The amorphous film of **R2-**doped *β*-estradiol showed blue fluorescence under excitation and red RTP after ceasing excitation (Supplementary Fig. 29c and Fig. 29d). The Φ_p of **R2** was measured to be 11.5%, which is lower than that of **4** (= 17.6%) and **5** (= 27.0%). The τ_p of **R2** was determined to be 92.9 ms from RTP decay characteristics (Supplementary Fig. 29e). From the equations $\Phi_p = \Phi_{isc} k_r^T \tau_p$, $\tau_p = 1/(k_r^T + k_{nr}^T)$, and $\Phi_{isc} = 1 - \Phi_f$, the values of k_r^T and k_{nr}^T of **R2** were determined to be 1.2 s^{-1} and 9.5 s^{-1}, respectively (Supplementary Table 3). Compared with **4** and **5**, the small enhancement of k_r^T relative to k_{nr}^T was observed in **R2** from **1** (green arrow in Supplementary Fig. 29f). The small enhancement of k_r^T relative to k_{nr}^T indicates that the contribution of spatial proximity between Se and DBC core to k_r^T enhancement is weak.

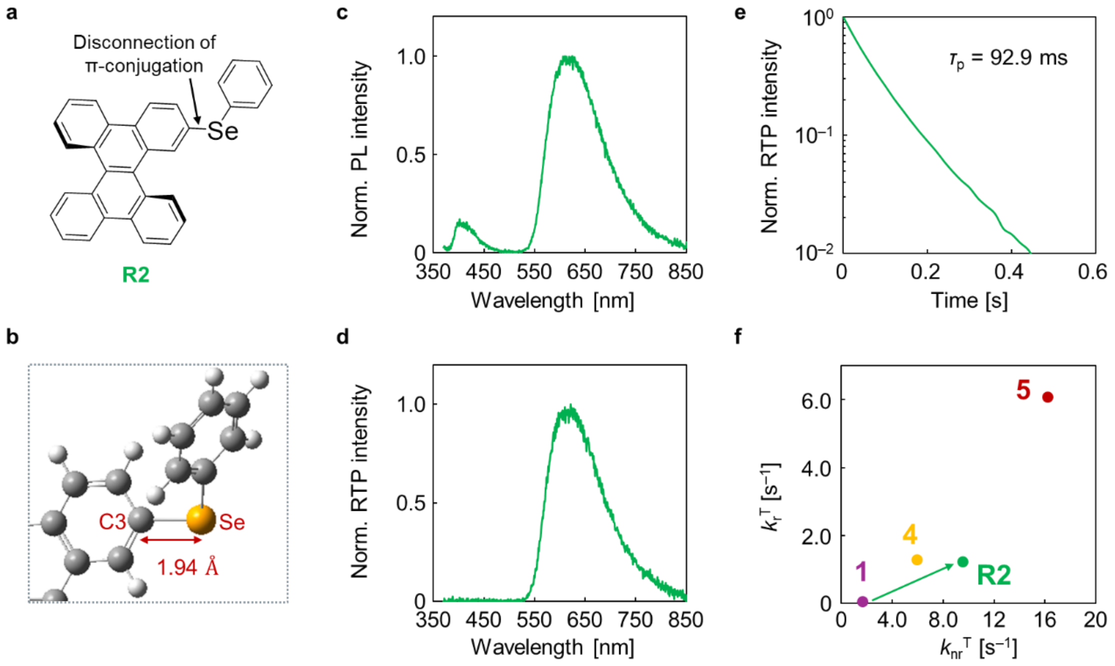

Supplementary Fig. 29. Photophysical characteristics of R2. a, Chemical structure of **R2**. **b,** Distance between Se atom and C atom at 3-position of DBC. **c**, Emission spectrum of **R2** in amorphous *β*-estradiol under excitation. **d,** RTP spectrum of **R2** in amorphous *β*-estradiol soon after ceasing excitation. **e,** RTP decay characteristics of **R2** in amorphous *β*-estradiol. **f,** Relationship between optically determined k_r^T and k_{nr}^T among **1**, **4**, **5**, and **R2**.

Supplementary Table 3. Photophysical parameters of **R2**.

Chromophores[a)]	Φ_f [%]	Φ_{isc}[b)] [%]	Φ_p [%]	τ_p [ms]	k_r^T [s^{-1}]	k_{nr}^T [s^{-1}]
**R2**	0.66	99.3	11.5	92.9	1.2	9.5

[a)]The concentration of chromophores was 0.3 wt% in amorphous *β*-estradiol. [b)] Values determined from 1 – Φ_f.

The relatively small enhancement of k_r^T compared with k_{nr}^T in **R2** could be confirmed from the different contributions of SOC to k_r^T and k_{nr}^T. Quantum chemical calculation confirmed that n = 6 is mostly contribute to k_r^T of **R2** (Supplementary Fig. 30a). In **R2**, the $\langle S_6|\mathbf{H}_{SO}|T_1\rangle^2$ that enhances k_r^T is smaller than $\langle T_1|\mathbf{H}_{SO}|S_0\rangle^2 \propto k_{nr}^T$ (arrow in Supplementary Fig. 30b).

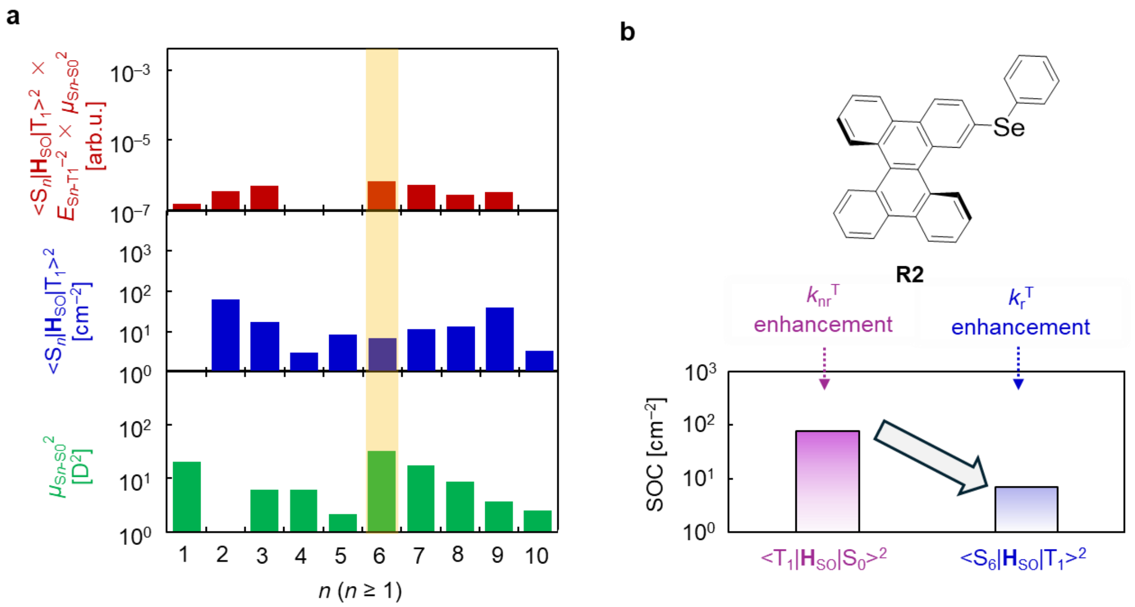

Supplementary Fig. 30. Calculated parameters relating to k_r^T in R2. a, Histograms of calculated parameters of $\frac{\langle S_n|\mathbf{H}_{SO}|T_1\rangle^2}{E_{S_n-T_1}{}^2}\mu_{S_n-S_0}{}^2$ (top), $<S_n|\mathbf{H}_{SO}|T_1>^2$ (middle), and $\mu_{S_n\text{-}S_0}{}^2$ (bottom) for **R2**. **b,** Histogram of the $<T_1|\mathbf{H}_{SO}|S_0>^2$ and $<S_6|\mathbf{H}_{SO}|T_1>^2$ values for **R2**.

The perspective of the orbital overlap factor that changes SOC strength provided a rational reason for the small enhancement of $<S_n|\mathbf{H}_{SO}|T_1>$ of **R2**. The S_6–S_0 transition of **R2** consists primarily of two components with 28.4% and 28.3% contributions, respectively. The former is composed of HOMO-2 and LUMO. The latter is composed of HOMO and LUMO+2. The overlap density between HOMO-2 and LUMO for S_6–S_0 transition [(HOMO-2)×(LUMO)] hardy overlaps with that between HOMO and LUMO for T_1 – S_0 transition [(HOMO)×(LUMO)] (Supplementary Fig. 31, left). Similarly, no spatial overlap was observed between the overlap density of HOMO and LUMO+2 for S_6–S_0 transition [(HOMO)×(LUMO+2)] and the (HOMO)×(LUMO) for T_1–S_0 transition (Supplementary Fig. 31, right). These lack of spatial overlap does not allow enhancement of $<S_6|\mathbf{H}_{SO}|T_1>$ in **R2**. Indeed, $<S_6|\mathbf{H}_{SO}|T_1>^2$ of **R2** is 6.97 cm^{-2}, which is much lower than $<S_7|\mathbf{H}_{SO}|T_1>^2$ of **4** (14.1 cm^{-2}), and $<S_2|\mathbf{H}_{SO}|T_1>^2$ of **5** (361 cm^{-2}). Therefore, simply placing the heavy atom in close proximity to the central π-core is insufficient. The orbital overlap selectively working on $<S_n|\mathbf{H}_{SO}|T_1>$, which is realized for **5**, is a key molecular design strategy for achieving a large k_r^T.

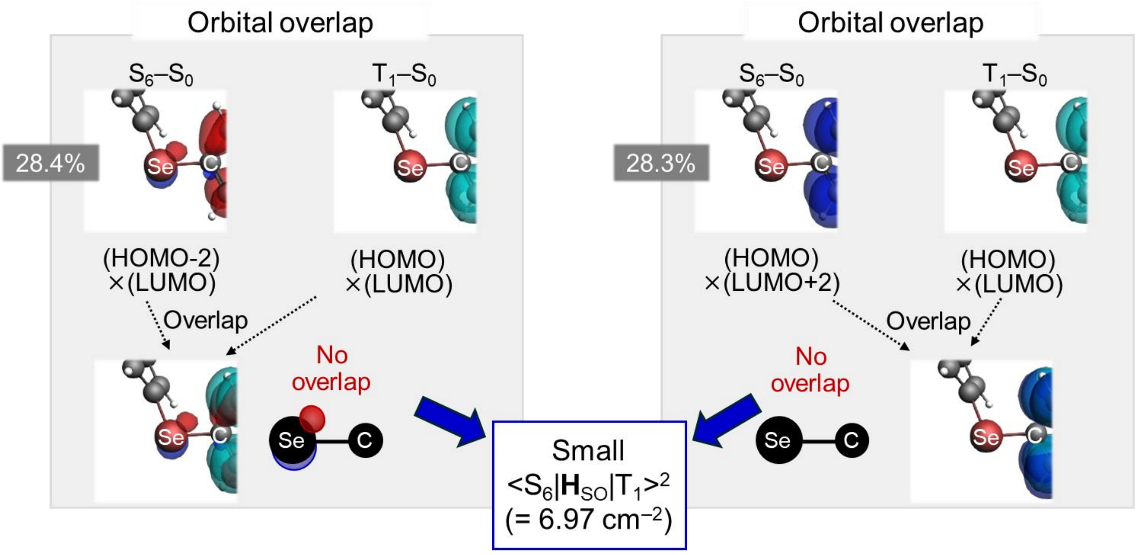

Supplementary Fig. 31. Molecular orbitals and schematic illustrations related to orbital overlap to explain small $<S_6|H_{SO}|T_1>^2$ of R2. The isovalues of the overlap density regarding (HOMO-2)×(LUMO), (HOMO)×(LUMO), and (HOMO)×(LUMO+2) are 0.00016.

Suppl. Note 5-7. Molecular orbitals relating to <S_n|H_{SO}|T_1>^{2} (Supplementary Fig. 32)

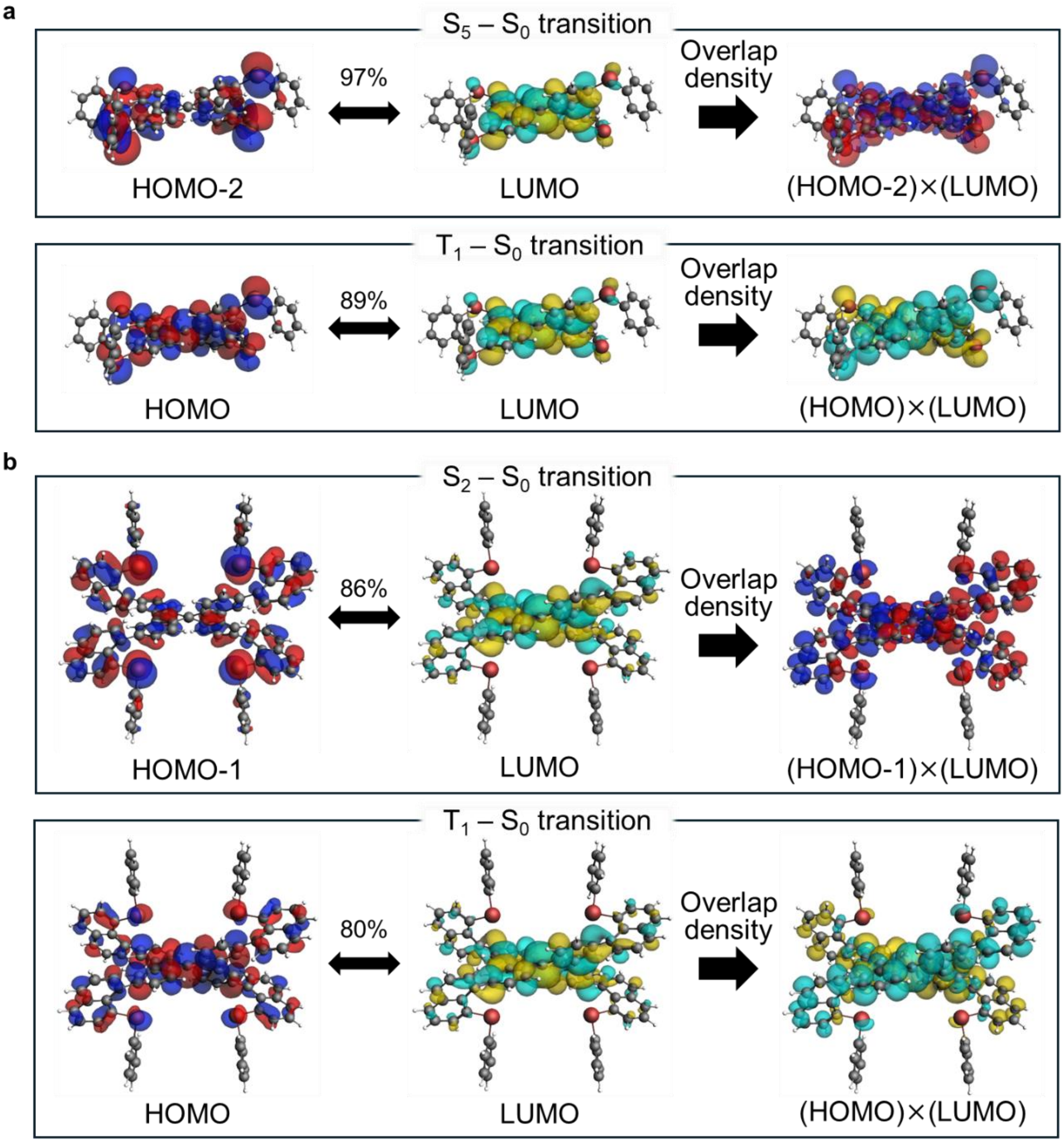

Supplementary Fig. 32. Molecular orbitals relating to <S_n|H_{SO}|T_1>^{2}. a, Molecular orbitals involved in the S_5–S_0 transition (top) and the T_1–S_0 transition (bottom) of **3**. **b,** Molecular orbitals involved in the S_2–S_0 transition (top) and the T_1–S_0 transition (bottom) of **5**. The isovalue of 0.015 is used for HOMO-2, HOMO, and LUMO for **3** and HOMO-1, HOMO, and LUMO for **5**. The isovalues of the overlap density regarding (HOMO-2)×(LUMO) and (HOMO)×(LUMO) for **3** and (HOMO-1)×(LUMO) and (HOMO)×(LUMO) for **5** are 0.00011.

Suppl. Note 5-8. Calculation results and photophysical properties of chromophore R1 (Supplementary Figs. 33–35 and Supplementary Table 4)

To evaluate the HAE contribution, chromophore **R1** in which Se atoms in **5** are replaced with S atoms was synthesized (Supplementary Fig. 33a). The **R1** was doped in the amorphous *β*-estradiol at a concentration with 0.3 wt%. The amorphous film of **R1**-doped *β*-estradiol showed blue fluorescence under excitation and red RTP after ceasing excitation (Supplementary Fig. 33b). The Φ_p was measured to be 10.4%, which is lower than **5** (Φ_p = 27.0%). The τ_p of **R1** was determined to be 416 ms from RTP decay characteristics (Supplementary Fig. 33c). From the equations $\Phi_p = \Phi_{isc}k_r^T\tau_p$, $\tau_p = 1/(k_r^T + k_{nr}^T)$, and $\Phi_{isc} = 1 - \Phi_f$, the values of k_r^T and k_{nr}^T for **R1** were determined to be 0.28 s^{-1} and 2.1 s^{-1}, respectively (Supplementary Table 4). Since k_r^T and k_{nr}^T of **5** are 6.1 s^{-1} and 16 s^{-1}, respectively, k_r^T largely decreases compared with k_{nr}^T depending on the change from **5** to **R1** (Supplementary Table 4).

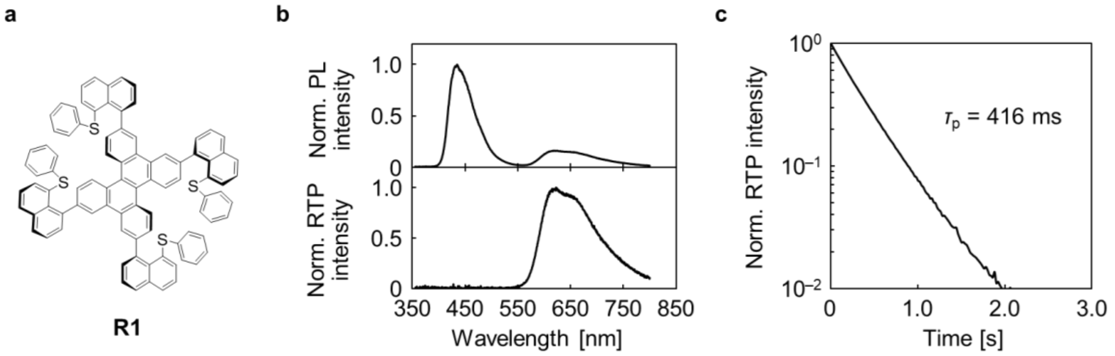

Supplementary Fig. 33. Optical properties of R1 in amorphous *β*-estradiol. a, Chemical structure of **R1**. **b,** Emission spectrum under excitation (top) and RTP spectrum soon after ceasing excitation (bottom) of 0.3 wt% **R1**-doped amorphous *β*-estradiol film. **c,** RTP decay characteristics of **R1** in amorphous *β*-estradiol.

Supplementary Table 4. Comparison of photophysical parameters relating RTP between **5** and **R1**.

Chromophores[a)]	Φ_f [%]	Φ_{isc}[b)] [%]	Φ_p [%]	τ_p [ms]	k_r^T [s^{-1}]	k_{nr}^T [s^{-1}]
**5**	1.0	99.0	27.0	44.8	6.1	16
**R1**	11.8	88.2	10.4	416	0.28	2.1

[a)]The concentration of chromophores was 0.3 wt% in amorphous *β*-estradiol. [b)] Values determined from $1 - \Phi_f$.

The decrease in k_r^T relative to k_{nr}^T of **R1** compared with **5** could be confirmed from the differences in the balance between $<T_1|\mathbf{H}_{SO}|S_0>^2$ and $<S_n|\mathbf{H}_{SO}|T_1>^2$. Quantum chemical calculation confirmed that $<S_3|\mathbf{H}_{SO}|T_1>^2$ mainly contributes to k_r^T of **R1** (Supplementary Fig. 34a). In **R1**, the $<S_3|\mathbf{H}_{SO}|T_1>^2$ is slightly smaller than $<T_1|\mathbf{H}_{SO}|S_0>^2$ (arrow of left graph in Supplementary Fig. 34b), while in **5**, $<S_2|\mathbf{H}_{SO}|T_1>^2$ is much larger than $<T_1|\mathbf{H}_{SO}|S_0>^2$ (arrow of right graph in Supplementary Fig. 34b). This calculation results regarding balance of $<T_1|\mathbf{H}_{SO}|S_0>^2$ and $<S_n|\mathbf{H}_{SO}|T_1>^2$ indicate that some factors are preventing $<S_3|\mathbf{H}_{SO}|T_1>^2$ from increasing in **R1**.

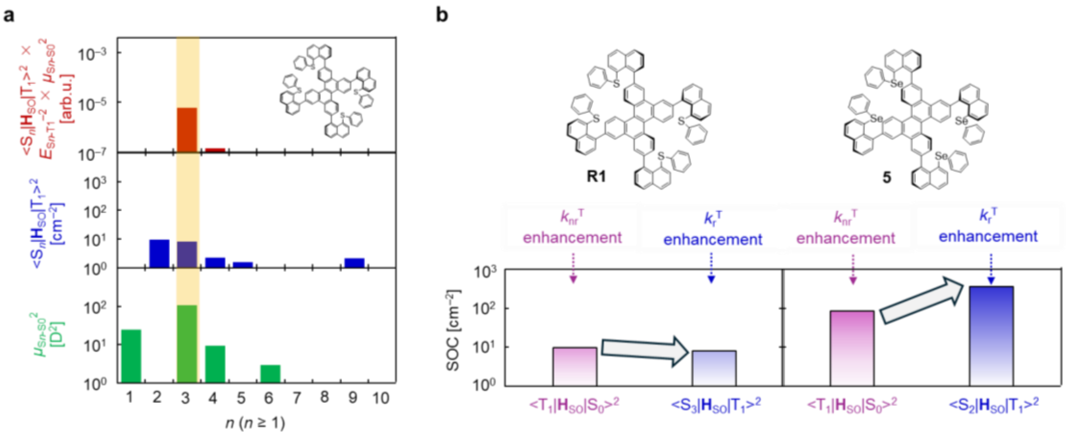

Supplementary Fig. 34. Calculated parameters relating k_r^T for R1. a, Histograms of calculated parameters of $\frac{\langle S_n|\mathbf{H}_{SO}|T_1\rangle^2}{E_{S_n-T_1}{}^2}\mu_{S_n-S_0}{}^2$ (top), $<S_n|\mathbf{H}_{SO}|T_1>^2$ (middle), and $\mu_{S_n-S_0}{}^2$ (bottom) for **R1**. **b,** Histograms of the $<T_1|\mathbf{H}_{SO}|S_0>^2$ and $<S_3|\mathbf{H}_{SO}|T_1>^2$ values for **R1** (left) and $<T_1|\mathbf{H}_{SO}|S_0>^2$ and $<S_2|\mathbf{H}_{SO}|T_1>^2$ values for **5**.

The perspective of the orbital overlap, HAE, orbital orientation factor provided a reason for the small $<S_3|\mathbf{H}_{SO}|T_1>^2$ of **R1**. First, regarding orbital overlap as a factor that changing $<S_n|\mathbf{H}_{SO}|T_1>$, for **R1**, the overlap density of the S_3–S_0 transition [(HOMO)×(LUMO+1)] hardy overlaps with that of T_1–S_0 transition [(HOMO)×(LUMO)] at through-space region between S and DBC (green open circle at the top of Supplementary Fig. 35(i)). On the other hand, for **5**, the overlap density of the S_2–S_0 transition [(HOMO–1)×(LUMO)] well overlaps with that of the T_1–S_0 transition [(HOMO)×(LUMO)] at through-space site between Se and DBC (green open circle at the bottom of Supplementary Fig. 35(i)). Therefore, the contribution of the orbital overlapping term to enhancement of $<S_3|\mathbf{H}_{SO}|T_1>$ for **R1** is significantly smaller than that to enhancement of $<S_2|\mathbf{H}_{SO}|T_1>$ in **5**. Second, the contribution from HAE, the distance between the overlap site and Se atom does not significantly change between **R1** and **5**

(Supplementary Fig. 35(ii)). However, the effect of HAE on **5** is larger than that on **R1** due to the heavier atoms. Third, regarding orbital orientation factor, for **R1**, the orbital axis of (HOMO)×(LUMO+1) and (HOMO)×(LUMO) are in different directions at through-space site (Supplementary Fig. 35(iii), top). Similarly, for **5**, the orbital axis of the through-space type (HOMO-1)×(LUMO) is different from that of the (HOMO)×(LUMO) on DBC (Supplementary Fig. 35(iii), bottom). Therefore, the orbital axis orientation hardly changes $\langle S_n|\mathbf{H}_{SO}|T_1\rangle$ between **R1** and **5**. When considering these three factors, in **5**, a through-space orbital overlap forms to selectively increases $\langle S_n|\mathbf{H}_{SO}|T_1\rangle$. Therefore, k_r^T increases relative to k_{nr}^T, acting as the driving force for the enhancement of the phosphorescence yield. On the other hand, in **R1**, the orbital overlap effect to selectively enhance $\langle S_n|\mathbf{H}_{SO}|T_1\rangle$ is not fully achieved. Therefore, a large $\langle S_3|\mathbf{H}_{SO}|T_1\rangle$ is not obtained, and k_r^T does not increase significantly relative to k_{nr}^T. Therefore, the phosphorescence yield does not improve significantly. Thus, the experimental and computational results regarding the replacement of Se with S indicates that the orbital overlap term is a key to maintenance of large $\langle S_n|\mathbf{H}_{SO}|T_1\rangle$.

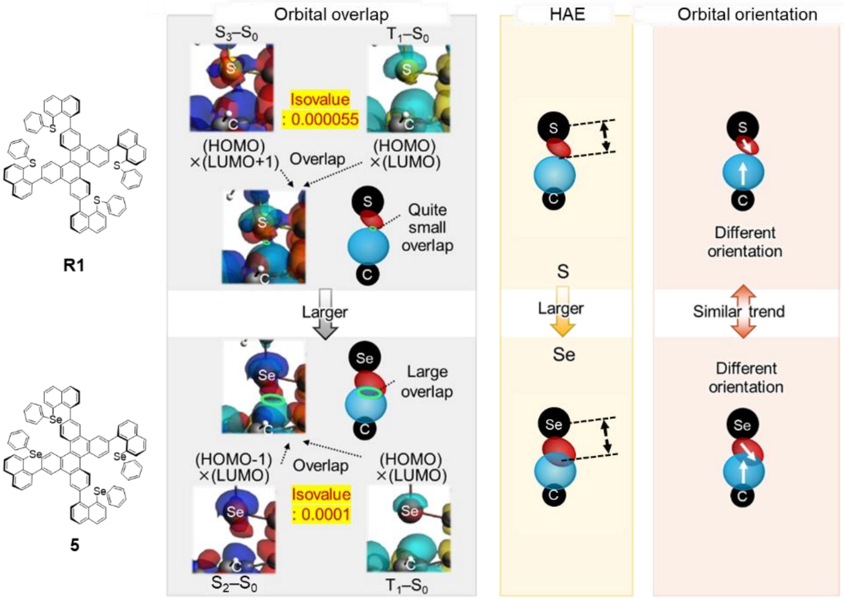

Supplementary Fig. 35. Molecular orbitals and schematic illustrations related to (i) orbital overlap, (ii) HAE, and (iii) orbital orientation to explain the smaller $\langle S_3|\mathbf{H}_{SO}|T_1\rangle^2$ of R1 (top) compared with $\langle S_2|\mathbf{H}_{SO}|T_1\rangle^2$ of 5 (bottom). The white arrows in (iii) represent the orbital axes orientations. The isovalues of the overlap density regarding (HOMO)×(LUMO+1) and (HOMO)×(LUMO) for **R1** is 0.000055. The isovalue of 0.0001 is used for the overlap density regarding (HOMO-1)×(LUMO) and (HOMO)×(LUMO) for **5**. When isovalue = 0.0001 is used for the overlap density regarding (HOMO)×(LUMO+1) and (HOMO)×(LUMO) for **R1**, no overlap region appears between Se and C.

Suppl. Note 5-9. Molecular orbitals relating to $<T_m|H_{SO}|S_0>^2$ (Supplementary Fig. 36)

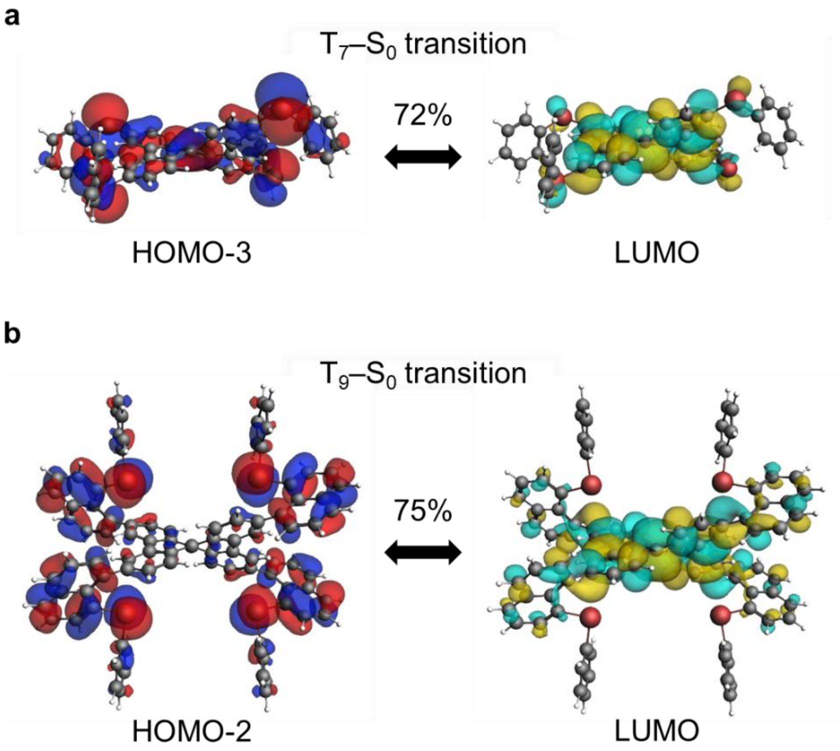

Supplementary Fig. 36. Molecular orbitals relating to $<T_m|H_{SO}|S_0>^2$. a, Molecular orbitals involved in the T_7–S_0 transition of **3**. **b,** Molecular orbitals involved in the T_9–S_0 transition of **5**. Isovalue of 0.013 is used for HOMO-3 and LUMO for **3** and HOMO-2 and LUMO for **5**.

Suppl. Note 6. Microscopic demonstrations

Suppl. Note 6-1. Procedure for preparing water-dispersible nanocrystals

The preparation procedure of crystalline particle A was described in the methods section in the main text. The water-dispersible crystalline particles B, C, and D were prepared as follows.

In particle B, (*S*)-BINAP-d_{32} and (±)-2,2'bis(diphenylphosphino)-5,5',6,6',7,7'8,8'-octahydro-1,1'-binaphthyl (H_8-BINAP) were used as a guest chromophore and a host molecule, respectively (Fig. 7b). Because the high-resolution afterglow imaging has been previously demonstrated using the (±)-2,2'-bis(diphenylphosphino)-1,1'-binaphthyl (BINAP)-doped H_8-BINAP crystalline particles[3], (*S*)-BINAP-d_{32}-doped H_8-BINAP particle have a potential to observe afterglow in a high-resolutions over a long time range due to the isotopic effect, which is suitable for our multi-label high-resolution imaging using the delay time. (*S*)-BINAP-d_{32} was dissolved in melted (*S*)-H_8-BINAP (Sigma-Aldrich, St Louis, MO, USA) at a concentration of 2.0 wt% at 240 °C, then cooled down to room temperature. The 2.0 wt%-(*S*)-BINAP-d_{32}-doped (*S*)-H_8-BINAP solid and (*R*)-(–)-2,2'bis(diphenylphosphino)-5,5',6,6',7,7'8,8'-octahydro-1,1'-binaphthyl ((*R*)-H_8-BINAP) (Sigma-Aldrich) at the weight ratio of 1 : 1 were mixed at the 240 °C to produce the H_8-BINAP crystalline solid doped with 1.0 wt% of (*S*)-BINAP-d_{32}. The crystalline solid of 1.0 wt%-(*S*)-BINAP-d_{32}-doped H_8-BINAP (1.0 mg) was added into the aqueous solution dissolved F127 (Sigma-Aldrich) (3.3 g L^{-1}, 1.0 ml). The mixture was stirred by using ultrasonic homogenizer (UR-20P, TOMY SEIKO CO., LTD., Tokyo, Japan) for 10 min in an ice bath. The mixture solution was filtrated through a hydrophilic membrane filter (Millex-AP, glass fibre, 2.0 μm, Merck Millipore Ltd., Tullagreen, Carrigtwohill, Ireland) with a specific pore size to remove the large particles. After centrifuging, the supernatant solution was removed, then pure water was added to the precipitation to prepare the water-dispersible crystalline particle B.

In particle C, **5d** and benzophenone were used as a guest chromophore and a host molecule, respectively (Fig. 7b). **5d** was added to the melted benzophenone at a concentration of 0.3 wt% at 90 °C, and mixture was quenched to room temperature to prepare the crystalline solid of benzophenone doped with **5d**. The **5d**-doped benzophenone crystals (5.0 mg) were added to the F127 aqueous solution (3.3 g L^{-1}, 1.0 ml) and dispersed by using ultrasonic homogenizer for 10 min in an ice bath. The mixture solution was filtered through a hydrophilic membrane filter with a pore size of 2.0 μm to remove the executively large crystals. After concentration by centrifugation, the supernatant solution was replaced with pure water to produce the particle C dispersed in water.

In particle D, 2,10-bis(phenylthio)dibenzo[*g*,*p*]chrysene-d_{16} (**S1**) and benzophenone were used as a guest chromophore and a host molecule, respectively (Fig. 7b)[3]. **S1** was added to the melted benzophenone at a concentration of 0.3 wt% at at 90 °C and mixture was quenched to room temperature to prepare the 0.3 wt%-**S1**-doped benzophenone crystalline solid. The crystalline solid of **5d**-doped benzophenone (5.0 mg) was added to the F127 aqueous solution (3.3 g L^{-1}, 1.0 ml). The mixture was stirred by using ultrasonic homogenizer for 10 min in an ice bath. The mixture solution was filtered through a hydrophilic membrane filter with a pore size of 2.0 μm to remove the executively large crystals. After concentration by centrifugation, the supernatant solution was removed, and pure water was added to the precipitation to produce particle D dispersed in water.

Suppl. Note 6-2. Biocompatibility of the water-dispersible nanocrystal (Supplementary Fig. 37)

To evaluate the biocompatibility of the water-dispersible crystalline particle, changes in cellular morphology were monitored by transmittance imaging after the injection of 0.3 wt% **5d**-doped crystalline benzophenone particles. Compared to the control condition in the absence of the particles (Supplementary Fig. 37(i)), the morphology of the cell membrane remained essentially unchanged throughout the observation period of at least 90 min in the presence of the particles (Supplementary Fig. 37(ii)). Meanwhile, the afterglow images confirmed the presence of the particles (Supplementary Fig. 37(iii)), indicating that the particles did not induce any noticeable cytotoxic effects under the experimental conditions.

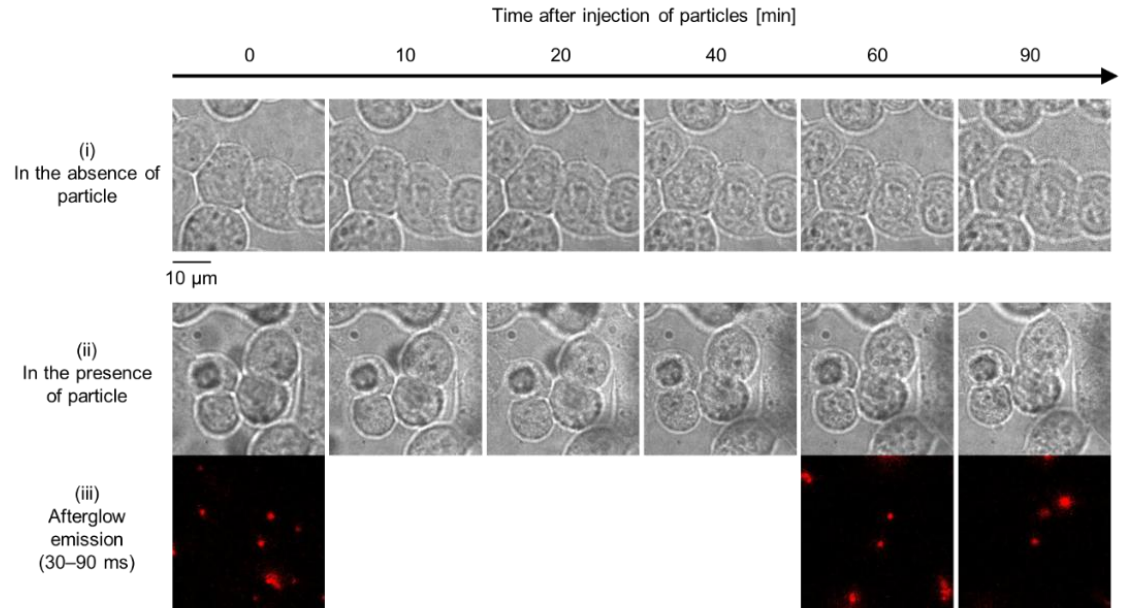

Supplementary Fig. 37. Biocompatibility of the water-dispersible nanocrystalline particles. (i,ii) Transmittance images of HEK293 cell culture sample in the absence (i) and the presence (ii) of 0.3 wt% **5d**-doped crystalline benzophenone particles, and (iii) afterglow images captured after ceasing excitation in the presence of the crystalline particles.

Suppl. Note 6-3. High-resolution multi-label afterglow imaging (Supplementary Figs. 38 and 39)

To demonstrate high-resolution multi-label afterglow imaging using the prepared particles, each of the aqueous solution (0.5 μL) dispersed the crystalline particle was injected into the cultured cells placed on polyethyleneimine-coated glass substrate. The cell cultured sample was mounted on the optical microscopy with an objective lens (×100) to capture the two-dimensional emission images in a high spatial resolution. Under excitation at 360 nm, significant autofluorescence was observed from the cell cultured sample when the long-pass filter above 405 nm was used (Supplementary Fig. 38a). After ceasing the excitation light, the high-resolution afterglow image was obtained without autofluorescence (Supplementary Fig. 38b). Although the brightness of the signal intensity after ceasing excitation was lower than that of under irradiation with excitation, afterglow intensity profile from the particles could be obtained clearly (Supplementary Figs. 38c and 38d). This is because the autofluorescence disappeared after the excitation was stopped. The signal count around 430 in afterglow intensity profile was not caused by autofluorescence but a dark count of the CCD camera. Therefore, if the afterglow intensity is too weak, the high-resolution afterglow image was hardly obtained due to the interference of dark count signals. However, particles A–D have bright afterglow capability that Φ_p of 1 wt% (*S*)-BINAP-doped (*S*)-H_8-BINAP, 1 wt% (*S*)-BINAP-d_{32}-doped H_8-BINAP, 0.3 wt% **5d**-doped benzophenone, 0.3 wt% **S1**-doped benzophenone are 9.1, 15, 46, 18%, respectively.

In addition to the mono-label afterglow imaging described in above, the use of an optical bandpass filter at 520 nm identified the location of particles A and B without autofluorescence at 20–60 ms after ceasing excitation (Supplementary Fig. 39c) because the afterglow emission from particles C and D was cut by the bandpass filter (Supplementary Fig. 39a). When the afterglow image was captured at 1000–1500 ms after ceasing excitation, the afterglow emission from only particle B could be detected (Supplementary Fig. 39d). This is because RTP from particle A was negligibly weak at the time range due to the short τ_p (Supplementary Fig. 39b, top). Furthermore, by using a long-pass filter at wavelength more than 685 nm (Supplementary Fig. 39a, bottom), the presence of particles C and D with red afterglow capability were observed at 20–60 ms after ceasing excitation (Supplementary Fig. 39e). Because the RTP of particle C rapidly decreased within 100 ms after ceasing excitation (Supplementary Fig. 39b, bottom), the afterglow information from only particle D was obtained by detecting the emission image at 120–400 ms after ceasing excitation (Supplementary Fig. 39f). Thus, by comparing the four afterglow images with different colour and time range, the locations of four particles

A–D were identified individually.

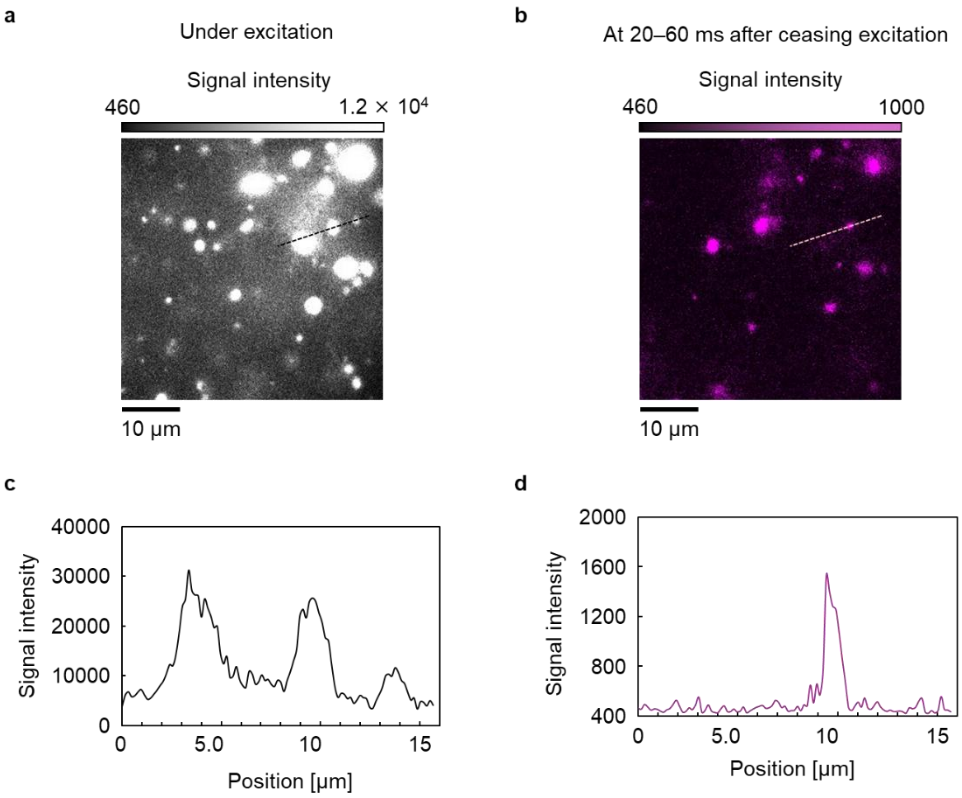

Supplementary Fig. 38. Two-dimensional emission images and emission profiles of HEK293 cell culture sample in the presence of particles A–D. a, Emission image under excitation. **b**, Afterglow emission image captured at 20–60 ms after ceasing excitation. **c**, Emission intensity profile at dashed line in Supplementary Fig. 38a. **d**, Afterglow emission profile at dashed line in Supplementary Fig. 38b. The excitation wavelength and intensity were 360 nm and 200 mW cm^{-2}, respectively. An optical long-pass filter at wavelength more than 405 nm was used to detect the emission signal from the sample.

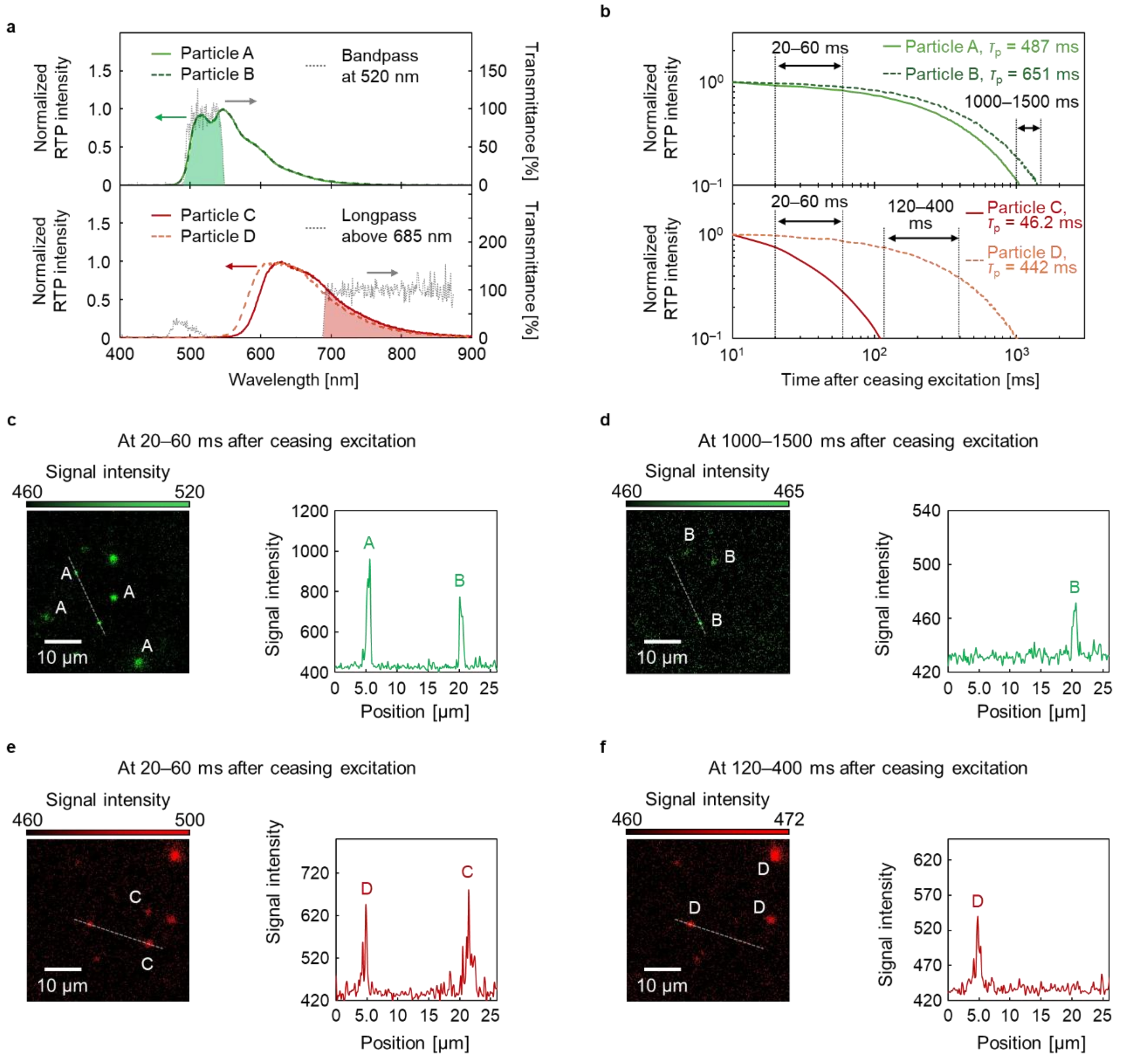

Supplementary Fig. 39. High-resolution multi-label afterglow imaging. a, RTP spectra of particles A–D after ceasing excitation and transmittance spectra of a bandpass filter at 520 nm and a long-pass filter above 685 nm. **b**, RTP decay characteristics of particles A–D. **c**, Afterglow image and intensity profile at 20–60 ms after ceasing excitation when the bandpass filter at 520 nm was used. **d**, Afterglow image and intensity profile at 1000–1500 ms after ceasing excitation when the bandpass filter at 520 nm was used. **e**, Afterglow image and intensity profile at 20–60 ms after ceasing excitation when the long-pass filter above 685 nm was used. **f**, Afterglow image and intensity profile at 120–400 ms after ceasing excitation when the long-pass filter above 685 nm was used. In **c**–**f**, the excitation wavelength and intensity were 360 nm and 200 mW cm^{-2}, respectively. The intensity profiles in **c**–**f** were corresponding to the dashed line in the afterglow images.

Supplementary References

1. Ueda, S., Fujita, K., Sk, B. & Hirata, S. Deciphering carbon–sulfur rotational distribution in a crystalline host for enhanced red persistent organic phosphorescence. *J. Mater. Chem. C* **13**, 2654–2660 (2025).
2. Yoshida, N. et al. Regio-defined multi-hydroxylation of dibenzo[*g*,*p*]chrysene. *Tetrahedron Lett.* **61**, 152033 (2020).
3. Mulimani, R. K. et al. Selective lower-occupied through-bond interactions for efficient organic phosphorescence enabling high-resolution long-wavelength afterglow. *Adv. Mater.* **37**, 2502611 (2025).
4. Suzuki, Y., Yamaguchi, M., Oketani, R. & Hisaki, I. Isomeric effect of naphthyl spacers on structures and properties of isostructural porous crystalline frameworks. *Mater. Chem. Front.* **7**, 106–116 (2023).
5. Fukasawa, K., Sugawara, Y., Tsuru, R., Yamashita, T. & Hirata, S. Enhanced red persistent room-temperature phosphorescence induced by orthogonal structure disruption during electronic relaxation. *J. Phys. Chem. Lett.* **13**, 7788–7796 (2022).
6. Hirata, S. Molecular physics of persistent room temperature phosphorescence and long-lived triplet excitons. *Appl. Phys. Rev.* **9**, 011304 (2022).
7. Shao, W. & Kim, J. Metal-free organic phosphors toward fast and efficient room-temperature phosphorescence. *Acc. Chem. Res.* **55**, 1573–1585 (2022).
