# Supplementary material for: Vertical substitution strategy to enable cooperation between spin–orbit coupling and transition dipoles for organic phosphorescence
Source: Nat Commun. 2026 Mar 17;17:4098. doi: 10.1038/s41467-026-70371-w (PMC13144389; doi:10.1038/s41467-026-70371-w)
Supplement: Supplementary file 2 — Description of Additional Supplementary File [file 41467_2026_70371_MOESM2_ESM.pdf]

Description of Additional Supplementary Files

File Name: Supplementary Data 1

Description: Atomic coordinates of **2** in the optimized geometry of the lowest triplet excited state calculated using the density functional theory (DFT).

File Name: Supplementary Data 2

Description: Atomic coordinates of **3** in the optimized geometry of the lowest triplet excited state calculated using the density functional theory (DFT).

File Name: Supplementary Data 3

Description: Atomic coordinates of **4** in the optimized geometry of the lowest triplet excited state calculated using the density functional theory (DFT).

File Name: Supplementary Data 4

Description: Atomic coordinates of **5** in the optimized geometry of the lowest triplet excited state calculated using the density functional theory (DFT).

File Name: Supplementary Data 5

Description: Atomic coordinates of **R1** in the optimized geometry of the lowest triplet excited state calculated using the density functional theory (DFT).

File Name: Supplementary Data 6

Description: Atomic coordinates of **R2** in the optimized geometry of the lowest triplet excited state calculated using the density functional theory (DFT).
